# Hydroxy Group-Assisted Cu-Catalyzed Asymmetric Conjugate Addition for the Creation of an All-Carbon Quaternary Center

**DOI:** 10.1021/acs.joc.6c00144

**Published:** 2026-05-18

**Authors:** Taiyo Yamamoto, Yuma Shiratori, Shogo Yamaguchi, Ken Yamanomoto, Kohei Endo

**Affiliations:** Department of Chemistry, Faculty of Science, 26413 Tokyo University of Science, Shinjuku, Tokyo 162-8601, Japan

## Abstract

Multinuclear Cu-catalyzed conjugate addition of Me_3_Al to α’-hydroxy enones was achieved in high to excellent enantioselectivity. In control experiments, we revealed that conjugate addition of Me_3_Al to β,β-disubstituted α’-hydroxy enones was an important contributor, even without ligands, which implies that the hydroxy moiety helps to facilitate the reaction. The corresponding products can be converted to various carbonyl compounds through oxidative cleavage of the C–C bond.

## Graphical abstract

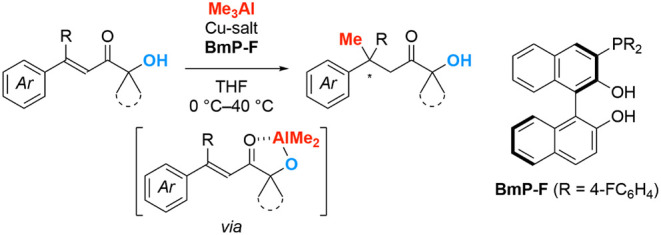

## Introduction

All-carbon quaternary stereogenic centers are ubiquitous motifs in natural products, pharmaceuticals and agrochemicals.^1^ However, their enantioselective construction remains challenging, especially for acyclic compounds due to their flexibility.^2^ Cu-catalyzed asymmetric conjugate addition is a robust synthetic method for constructing tertiary or quaternary stereogenic carbon centers. Hoveyda and Fillion independently reported the Cu-catalyzed conjugate addition of organozinc reagents for the construction of chiral quaternary center.^3^ Hoveyda developed chiral NHC ligands in the Cu-catalyzed conjugate addition of organoaluminum reagents and organoboron compouds.^4,5^ The organozirconium intermediates and Grignard reagents can participate in the enantioselective conjugate addition reactions.^6,7^ Based on this background, we have been developing multinuclear Cu-catalyzed asymmetric conjugate addition of organoaluminum reagents to β,β-disubstituted α,β-unsaturated ketones and α,β-unsaturated ketoesters, which furnish all-carbon quaternary stereogenic centers in high to excellent enantioselectivity.^8^ The products bearing methyl-containing chiral carbons are useful building blocks for biologically active compounds; hence, we further examine the synthetic utility of 1,4-adducts using Me_3_Al.^9a^ Among various enones, α’-hydroxy enones have the potential to offer numerous synthetic routes, as they can be converted to carbonyl compounds through oxidative cleavage of the C–C bond.^10^ Additionally, the hydroxy group on α’-hydroxy enones promotes the simultaneous activation and interaction with catalysts that increase the reaction rate and enantioselectivity in the Diels–Alder reaction,^11^ Friedel–Crafts reaction,^12^ and (radical)­conjugate addition^13^ in the presence of Lewis acid or Brønsted acid. Palomo and coworkers reported the asymmetric conjugate addition of organozinc reagents to α’-oxy enones (Scheme 1a).^14^ In this report, the protecting group on hydroxy moiety plays an essential role in achieving high enantioselectivity. This result can be explained by considering that the uncatalyzed pathway could be promoted by strong Lewis acidic activation in the absence of a protecting group on the hydroxy moiety. Based on this idea, we envisioned that a strongly chelating hydroxy group contributed to the intramolecular activation of the substrates. Herein, we report a multinuclear Cu-catalyzed asymmetric conjugate addition of Me_3_Al to β,β-disubstituted α’-hydroxy enones (Scheme 1b).

**Scheme 1:**
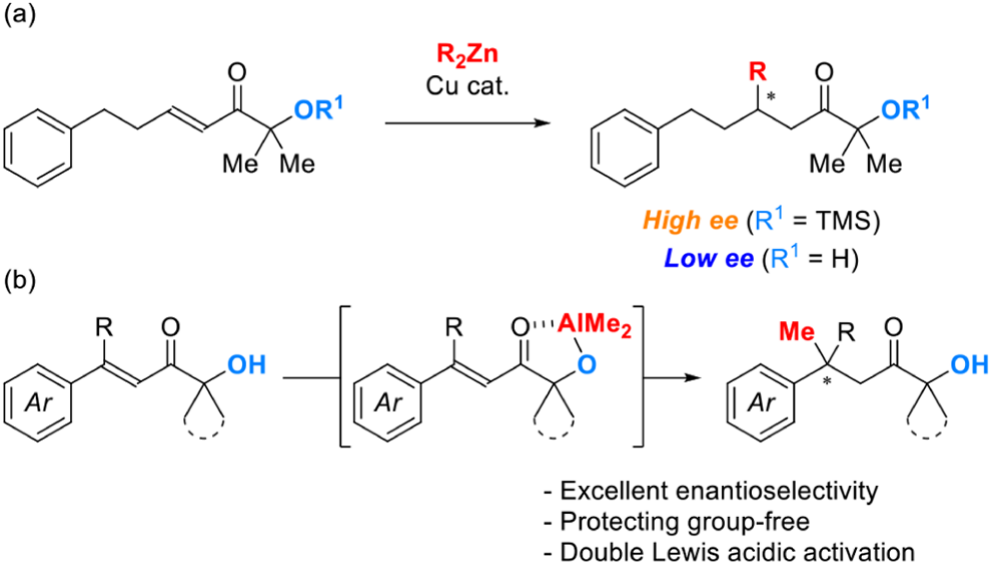
Previous Work and Present Work. (a) Previous Work (Palomo et al.). (b) This Work

## Results and Discussion

We initially examined a series of originally developed ligands; **BP**, **BmP**, and **SP**, for the asymmetric conjugate addition of Me_3_Al to β,β-disubstituted α’-hydroxy enones **1a** (Figure 1). The use of **BmP** (10 mol %) with CuI (5 mol %) for the conjugate addition of Me_3_Al (4 equiv) to **1a** in THF (0.1 M) at 0 °C to rt gave the desired product **2a** in good yield and good enantioselectivity, while **BP** and **SP** gave poor results. The smaller amount of Me_3_Al was not effective. We further examined the steric and electronic effects on the phosphine ligands. In each case, the desired compound **2a** was obtained in good to excellent yield though sterically bulky and electron-rich phosphine ligands tended to give **2a** in low enantioselectivity. Based on this finding and reports in the literature,^15^ we developed sterically less bulky and electron-deficient **BmP** derivative **BmP-F**, which gave **2a** in 83% yield and 90% ee. We further tuned the reaction conditions and found that the use of **BmP-F** (10 mol %) as a ligand and CuI (5 mol %) as a Cu-salt for the conjugate addition of Me_3_Al (4 equiv) at 0 to 40 °C in THF and hexane for 24 h gave **2a** in 81% yield and 96% ee (see, the Supporting Information).

**Figure 1:**
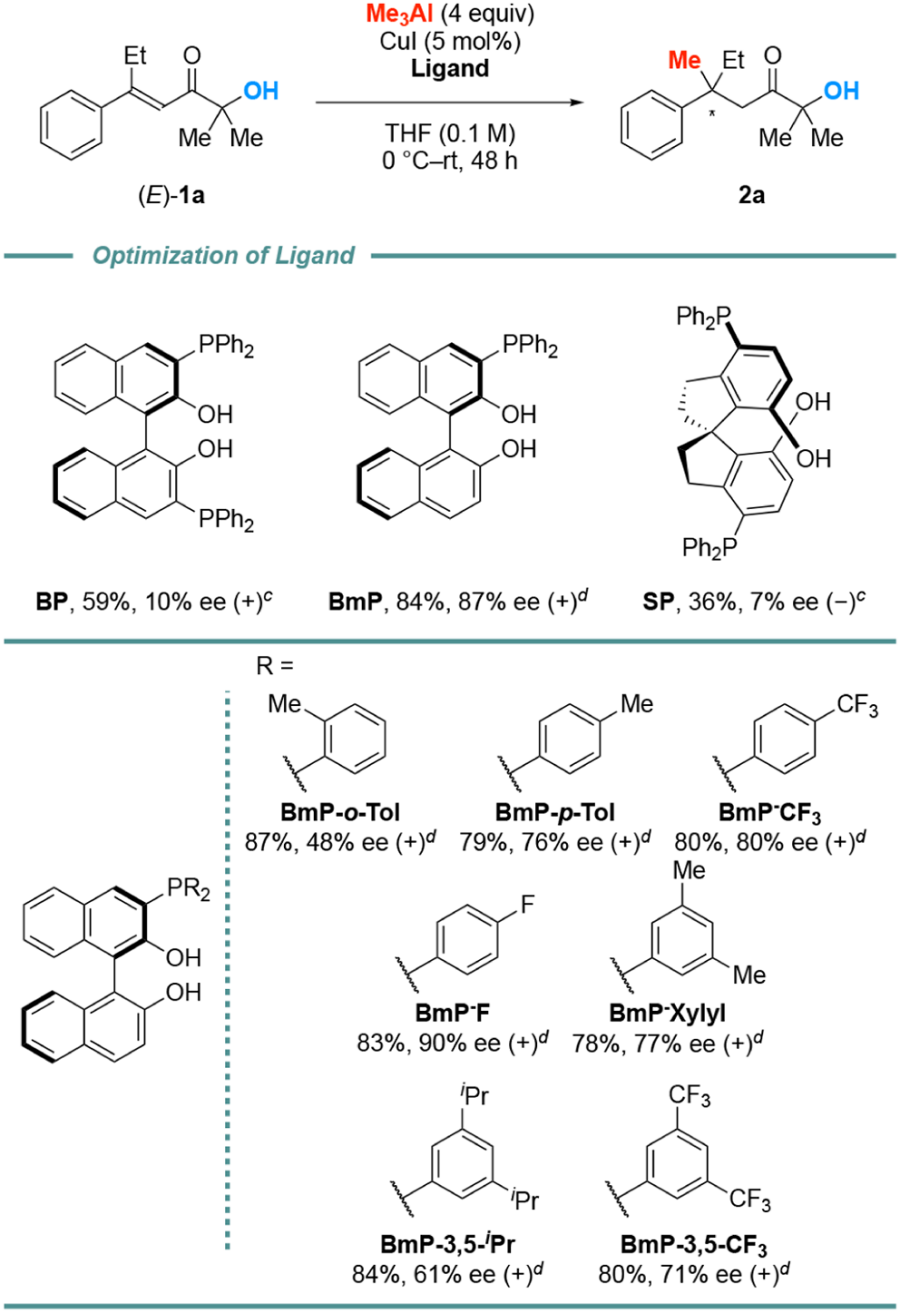
Optimization of Ligand*^a,b^*. *^a^* Me_3_Al (1.4 M in hexane) was used. *^b^* The ee was determined by chiral HPLC analysis. *^c^* Ligand (5 mol %) was used. *^d^* Ligand (10 mol %) was used.

With the optimal reaction conditions in hand, we investigated the substrate scope (Figure 2, top). The reaction gave the *para*-substituted products in high yield with excellent enantioselectivity; electron-neutral (−Me in **2b**, −Ph in **2e**), electron-donating (−OMe in **2c**) and electron-withdrawing (−F in **2d**) groups were compatible with the reaction conditions. The sterically hindered products were also obtained with a slight decreased in enantioselectivity (**2f**). The π-extended derivatives (**2g**, **2h**) and the heteroaromatic furanyl (**2i**) and thiophenyl (**2j**) derivatives were obtained in excellent enantioselectivity, and product **2h** was isolated in moderate yield due to the purification problem.

**Figure 2:**
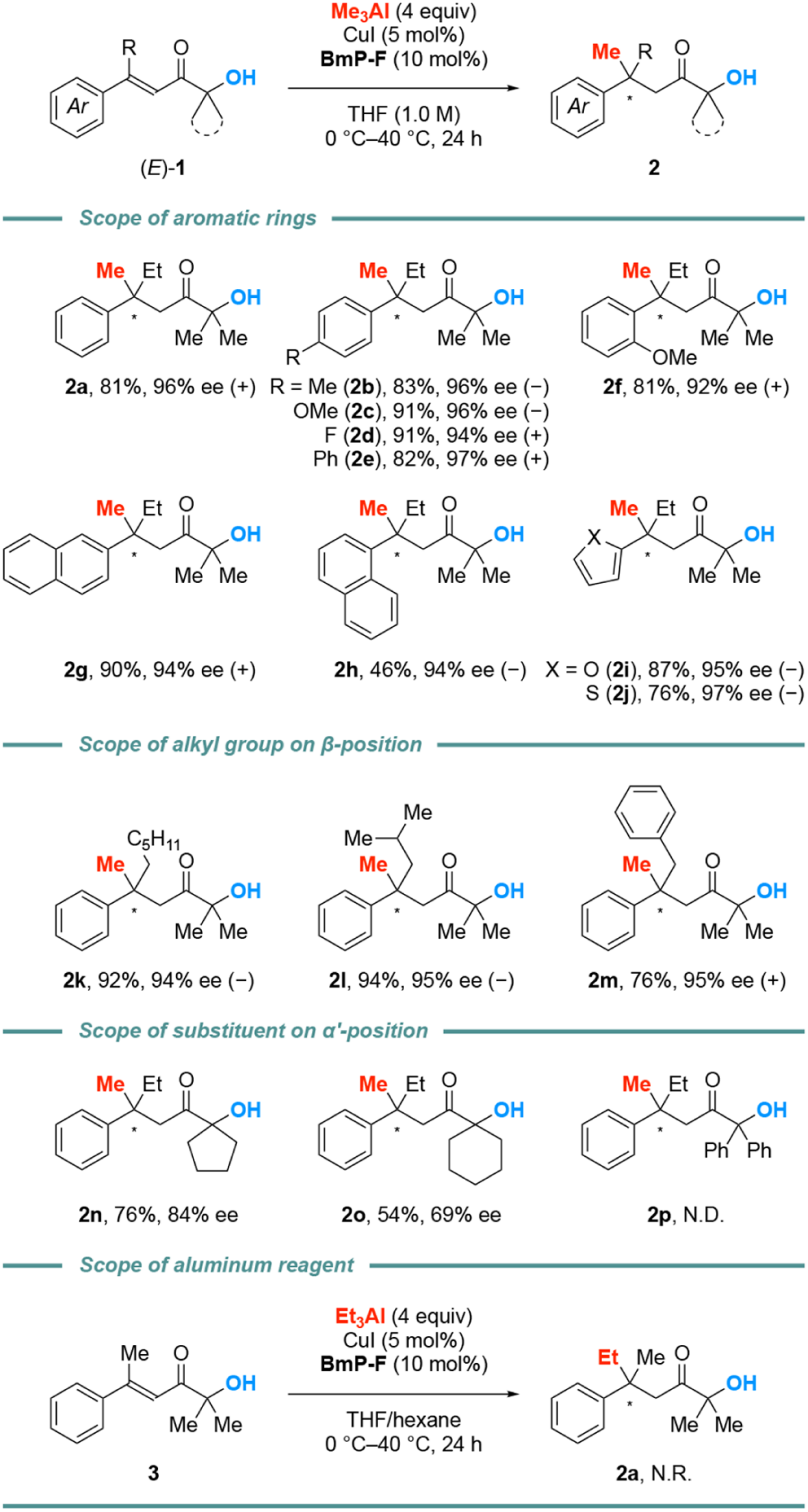
Scope of substrates*^a^*. *^a^* The reaction of α’-hydroxy enones (0.20 mmol) and Me_3_Al (4 equiv) was carried out in the presence of CuI (5 mol %) and **BmP-F** (10 mol %) in THF/hexane at 0–40 °C. Me_3_Al (1.4 M in hexane) was used.

We next investigated the scope of the alkyl group at the β-position, substituents at the α’-position and alkylaluminum reagents (Figure 2, middle and bottom). The alkyl group at the β-position does not affect the reaction outcome (**2k**–**2m**). The steric bulkiness at the α’-position is correlated with enantioselectivity and the reaction with substrate **1p** did not afford the desired product **2p**. In contrast, the asymmetric conjugate addition of Et_3_Al to α’-hydroxy enones (**3**) did not occur. This could be explained by both the lower Lewis acidity of Et_3_Al and steric hindrance around the carbonyl oxygen because of coordination to an aluminum atom. The present catalyst system is not suitable for enones bearing a bulky carbonyl group; thus, more sterically hindered Et_3_Al would not work efficiently. We tentatively carried out the deprotonation reaction of (*E*)-**1a** using catalytic amount of Me_3_Al and the subsequent addition of Et_3_Al (4 equiv) under the standard conditions. However, the reaction did not proceed.

We next investigated the effect of the olefinic geometry. The optical sign of the product **2a** obtained from (*Z*)-**1a** was opposite that of **2a** from (*E*)-**1a** and the enantiomer excess (ee) was dramatically decreased (Scheme 2, top). To gain deeper insight into the reaction, we conducted several control experiments (Scheme 2, middle). We revealed that isomerization from (*Z*)-isomer to (*E*)-isomer occurred under Lewis acidic conditions. Interestingly, Cu-catalyzed conjugate addition to β,β-disubstituted α’-hydroxy enones occurred even without **BmP-F**. In contrast, the substrate **4** with a Me group instead of a OH group did not react under the same condition. Furthermore, protection of the OH moiety with a trimethylsilyl (TMS) group inhibits the reaction (Scheme 2, bottom). These observations imply that OH group on the α’-position facilitates the reaction. We also examined the suitability of a Weinreb amide. (*E*)-*N*-methoxy-*N*-methyl-3-phenylpent-2-enamide was subjected to the optimal reaction conditions; however, the desired product was not obtained, and the starting material was recovered.

**Scheme 2:**
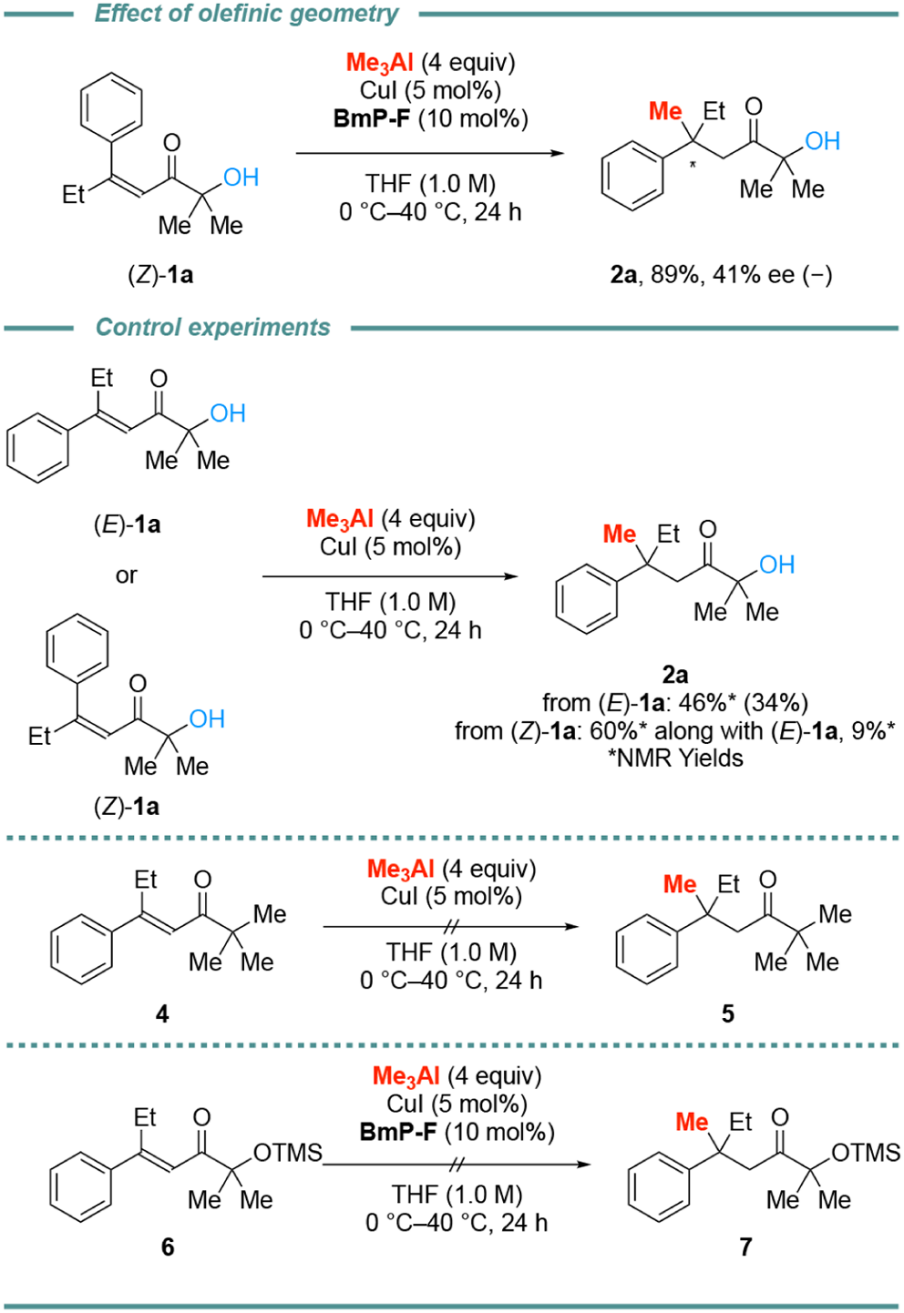
Effect of Olefinic Geometry and Control Experiments^a^

a Me_3_Al (1.4 M in hexane) was used.

To demonstrate the synthetic utility of this scheme, we conducted 1 mmol scale synthesis (Scheme 3). The product **2a** was successfully obtained with negligible changes in yield and enantioselectivity. The resulting product **2a** was further converted to carboxylic acid and aldehyde through oxidative cleavage of the C–C bond. The carboxylic acid **8** was obtained quantitatively. The optical sign of the carboxylic acid **8** matched that in previous reports,^16^ which confirmed that the absolute configuration was *R*. The stereochemistry of the carboxylic acid **8** indicates that the present stereoselectivity is the same as that in a previous catalyst system.^8^ The enantio purity of the carboxylic acid **8** was determined by measuring one of the corresponding aldehyde **9**, which was obtained by methylation, followed by DIBAL-H reduction. We found that the carboxylic acid **8** was successfully obtained with negligible changes in enantiomer excess (96% ee). The aldehyde **9** derived from **2a** was obtained in synthetically useful yield without a loss of enantiopurity.

**Scheme 3:**
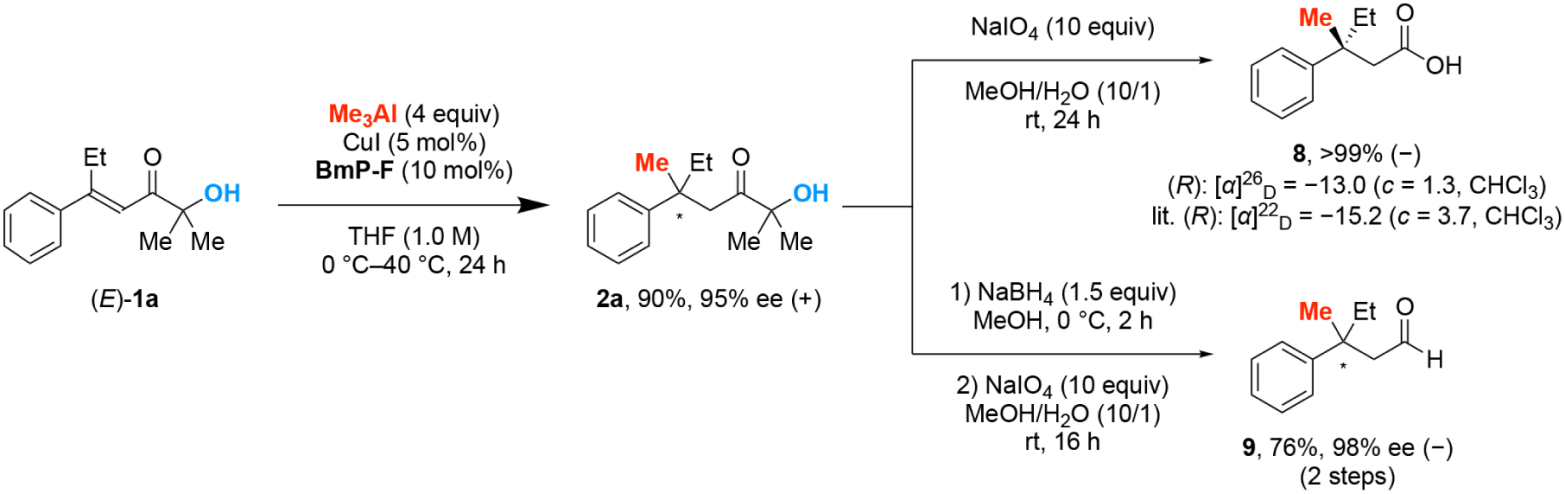
1 mmol Scale Synthesis and Application of the Product

The proposed reaction mechanism based on previous reports^8,17^ is shown in Scheme 4. The reaction of CuI, Me_3_Al and **BmP-F** gives a CuMe-complex I and Me_2_Al–I. The resulting Me_2_Al–I coordinates to the deprotonated α’-hydroxy enones and assists the construction of π-complex II (see, the Supporting Information). In a control experiment, the OH group plays a crucial role in activating substrates, which indicates that a carbonyl group could coordinate with two different aluminum atoms and double Lewis acidic activation could contribute. Oxidative addition and reductive elimination of the copper catalyst complete the catalytic cycle. Protonation of the resulting enolate gives the desired product.

**Scheme 4:**
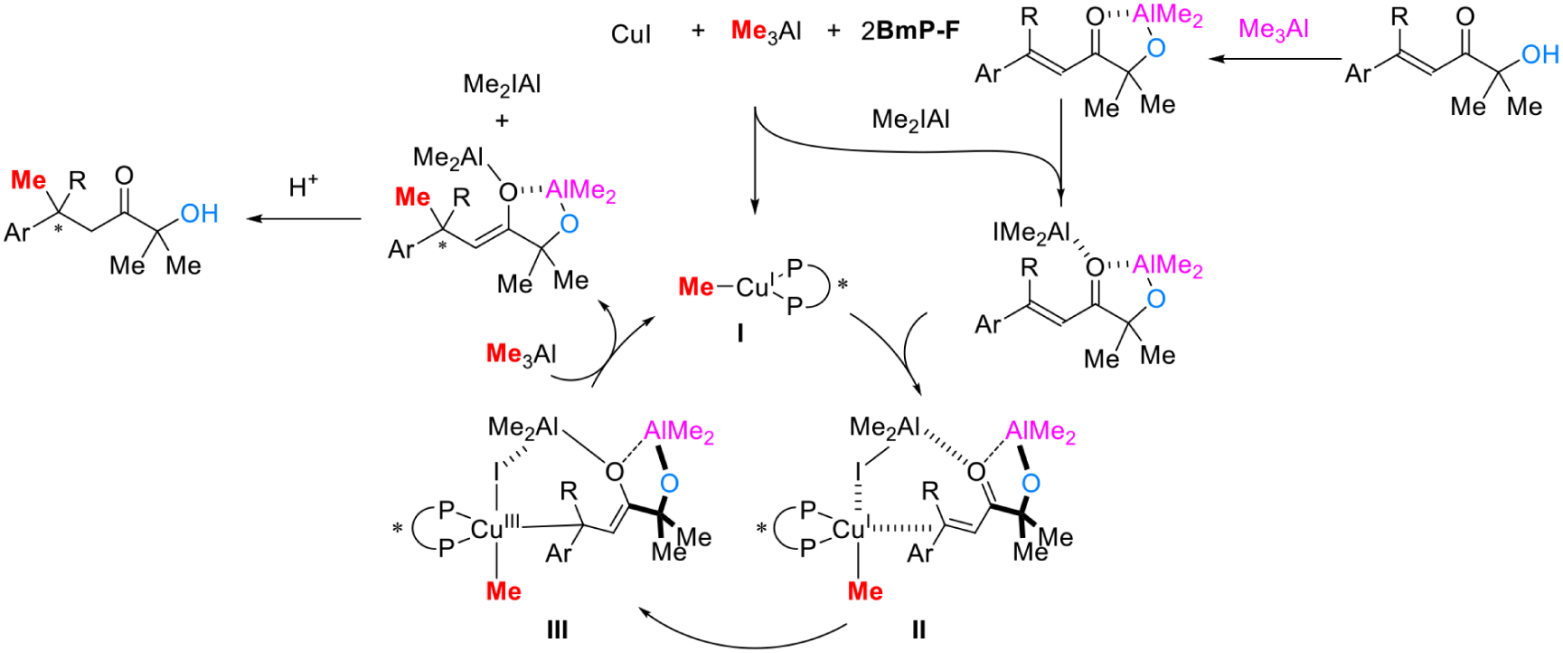
Plausible Reaction Mechanism

We postulate a plausible mechanism for stereoselectivity (Scheme 5). In the optimization study, yield and enantioselectivity are dramatically affected by the counteranion of Cu salt. This indicates that the anion is included in the enantio-determining step. On the basis of the result of ESI-TOF-MS analyses of Cu complexes derived from **BmP**, an Al-linked **BmP**-Cu complex is one of the possible structures for effective stereoselectivity of conjugate addition.^8a^ Hence, a Me_2_Al fills the sterically less hindered upper left or lower right space shown in the proposed transition-state model in Scheme 5 and fixes the enone. The addition of a Me group occurs from the *re*-face and furnishes an all-carbon stereogenic center.

**Scheme 5:**
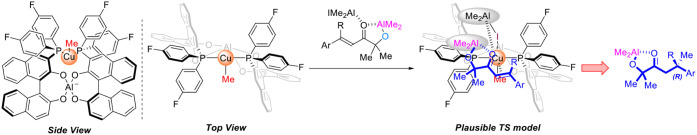
Proposed Structure of Multinuclear Cu/Al/BmP-F-Complex

## Conclusion

In summary, we achieved the construction of all-carbon quaternary stereogenic centers through multinuclear-Cu/Al-complex catalyzed asymmetric conjugate addition of Me_3_Al to β,β-disubstituted α’-hydroxy enones in excellent enantioselectivity. We also demonstrated the synthetic utility of this scheme by converting the product into carboxylic acid and aldehyde without a loss of enantiopurity. The neighboring hydroxy group in bulky enones enhances the Cu-catalyzed conjugate addition of Me_3_Al in the absence of a **BmP** ligand, in which the hydroxy group-mediated intramolecular activation approach seems to be effective for substrates that resist reactions due to their steric bulkiness. The development of intra- and intermolecular double activation would contribute to the effective conversion of unreactive molecules.

## Experimental Section

### General Method

All the reactions dealing with air or moisture sensitive compounds were carried out in a dry reaction vessel under positive pressure of argon. Air- and moisture-sensitive liquids and solutions were transferred via a syringe. Analytical thin layer chromatography was performed with aluminum TLC plate (Merck, #1.05554.0001). Thin layer chromatography plates were visualized by exposure to ultraviolet light (254 nm) and/or by immersion in an acidic staining solution of p-anisaldehyde, molybdatophosphoric acid, and potassium permanganate alkaline, followed by heating. Organic solutions were concentrated by rotary evaporation. Flash column chromatography was performed on Kanto Silica gel 60 (spherical, neutral, particle size 40–50 μm). NMR spectra were recorded on JEOL ECZ400S or Bruker-Biospin AVANCE NEO 400 spectrometer and measured for^1^H, ^13^C, ^19^F, and ^31^P NMR using tetramethylsilane as an internal reference and trifluoroacetic acid as external references and CDCl_3_ as a solvent. Chemical shift values for protons are reported in parts per million (ppm, δ scale) downfield from tetramethylsilane and are referenced to residual proton of CDCl_3_ (δ 7.26). Carbon nuclear magnetic resonance spectra (^13^C NMR) were recorded at 100 MHz: chemical shifts for carbons are reported in parts per million (ppm, δ scale) downfield from tetramethylsilane and are referenced to the carbon resonance of CDCl_3_ (δ 77.0). Fluorine nuclear magnetic resonance spectra (^19^F NMR) were recorded at 375 MHz: chemical shifts for fluorine are reported in parts per million (ppm, δ scale) referenced to the fluorine resonance of trifluoroacetic acid (δ – 76.5). Phosphorus nuclear magnetic resonance spectra (^31^P NMR) were recorded at 162 MHz: chemical shifts for phosphorus are reported in parts per million (ppm, δ scale) referenced to the phosphoric acid (δ 0). Data are presented as following space: chemical shift, multiplicity (s = singlet, d = doublet, t = triplet, q = quartet, quint = quintet, sext = sextet, sept = septet, m = multiplet and/or multiplet resonances), coupling constant in hertz (Hz), and signal area integration in natural numbers, assignment (italic). Mass spectra were measured using ESI-MS or APCI-MS. HRMS were collected using TOF. All reagents were purchased as commercially available source unless otherwise noted. (*R*)-BINOL (>99.0% ee) was purchased from Fuji Molecular Planning Co., Ltd. BP, BmP, BmP-*o*-Tol, BmP-*p*-Tol, BmP-Xylyl, BmP-3,5-CF_3_, and SP were synthesized according to previous reports.^8,18^

**BmP-CF_3_:** To a solution of MOM-protected (*R*)-BINOL (2.5 mmol, 0.94 g) in THF (18 mL), a solution of ^*t*^BuLi in pentane (3 mmol, 2.0 mL, 1.55 M) was added dropwise at −78 °C. After stirring 5 h at this temperature, chlorodi­(4-trifluoromethylphenyl)­phosphine (3 mmol, 1.07 g) was added. The mixture was allowed to warm to rt and stirred for 30 min. The reaction mixture was quenched with sat. NaHCO_3_ aq., filtered through a pad of silica gel and concentrated to give a residue which was treated with cat. HCl aq. in MeOH (18 mL) under reflux condition for 16 h. The reaction mixture was neutralized with sat. NaHCO_3_ aq., dried over anh. Na_2_SO_4_, filtered and concentrated to give a crude product which was purified by silica gel chromatography (hexane/EtOAc = 5/1) to afford BmP-CF_3_ (0.11 g, 7%): white solid; mp 123.8–124.5 °C; ^1^H NMR (400 MHz, CDCl_3_) δ 7.95 (d, *J* = 9.0 Hz, 1H), 7.87 (d, *J* = 7.8 Hz, 1H), 7.77–7.60 (m, 5H), 7.59–7.50 (m, 4H), 7.44–7.28 (m, 6H), 7.20–7.08 (m, 2H), 5.33–5.29 (m, 1H), 5.00 (s, 1H); ^13^C­{^1^H} NMR (100 MHz, CDCl_3_, peaks are very complicated due to the coupling between C and F) δ 153.3 (d, *J* = 13.8 Hz), 152.7, 140.2 (dd, *J* = 7.3, 13.1 Hz), 136.0 (d, *J* = 4.4 Hz), 134.4, 134.2, 134.1, 134.0, 133.1, 131.7, 131.4 (dd, *J* = 8.7, 32.7 Hz), 129.5, 129.3 (d, *J* = 2.2 Hz), 128.7, 128.6, 128.4, 127.6, 125.7–125.2 (multiplet), 125.0, 124.8, 124.6, 124.2, 124.2, 123.8, 122.6, 117.8, 111.5, 110.2; ^19^F NMR (375 MHz, CDCl_3_) δ – 62.8; ^31^P NMR (162 MHz, CDCl_3_) δ – 14.7; HRMS (APCI, positive) *m*/*z*: [M + H]^+^ calcd. for C_34_H_22_F_6_O_2_P^+^ 607.1256; found 607.1256.

**BmP-F:** To a solution of MOM-protected (*R*)-BINOL (3.5 mmol, 1.31 g) in THF (25 mL),was added a solution of ^*t*^BuLi in pentane (3.5 mmol, 2.3 mL, 1.55 M) was added dropwise at −78 °C. After stirring 5 h at this temperature, chlorodi­(4-fluorophenyl)­phosphine (4 mmol, 1.03 g) was added. The mixture was allowed to warm to rt and stirred for 30 min. The reaction mixture was quenched with sat. NaHCO_3_ aq., filtered through a pad of silica gel and concentrated to give a residue which was treated with cat. HCl aq. in MeOH (25 mL) under reflux condition for 16 h. The reaction mixture was neutralized with sat. NaHCO_3_ aq., dried over anh. Na_2_SO_4_, filtered and concentrated to give a crude product which was purified by silica gel chromatography (hexane/EtOAc = 2/1) to afford BmP-F (0.43 g, 24%): white solid; mp 115.6–116.0 °C; ^1^H NMR (400 MHz, CDCl_3_) δ 7.90 (d, *J* = 8.8 Hz, 1H), 7.84 (d, *J* = 7.5 Hz, 1H), 7.69–7.63 (m, 1H), 7.45–7.25 (m, 10H), 7.17–7.04 (m, 6H), 5.33 (s, 1H), 5.01 (s, 1H); ^13^C­{^1^H} NMR (100 MHz, CDCl_3_, peaks are very complicated due to the coupling between C and F) δ 164.8 (d, *J* = 6.5 Hz), 162.4 (d, *J* = 5.8 Hz), 153.3 (d, *J* = 14.5 Hz), 152.6, 135.9 (ddd, *J* = 8.2, 22.3, 26.0 Hz), 135.4 (d, *J* = 2.9 Hz), 133.8, 133.1, 131.5, 131.1 (td, *J* = 9.9, 3.2 Hz), 129.4, 129.3, 128.5, 128.5, 128.0, 127.5, 127.1, 127.0, 124.3, 124.1, 124.0, 123.9, 117.7, 116.0 (ddd, *J* = 8.0, 13.4, 21.1 Hz), 111.1, 110.5; ^19^F NMR (375 MHz, CDCl_3_) δ – 111.34, – 111.56; ^31^P NMR (162 MHz, CDCl_3_) δ – 17.5; HRMS (APCI, positive) *m*/*z*: [M + H]^+^ calcd for C_32_H_22_F_2_O_2_P^+^ 507.1320; found 507.1320.

**BmP-3,5-*^i^* Pr:** To a solution of MOM-protected (*R*)-BINOL (2 mmol, 0.75 g) in THF (14 mL), a solution of ^*t*^BuLi in pentane (2 mmol, 1.3 mL, 1.55 M) was added dropwise at −78 °C. After stirring 5 h at this temperature, chlorodi­(3,5-diisopropylphenyl)­phosphine (4 mmol, 1.56 g) was added. The mixture was allowed to warm to rt and stirred for 30 min. The reaction mixture was quenched with sat. NaHCO_3_ aq., filtered through a pad of silica gel and concentrated to give a residue, which was treated with cat. HCl aq. in MeOH (14 mL) under reflux condition for 16 h. The reaction mixture was neutralized with sat. NaHCO_3_ aq., dried over anh. Na_2_SO_4_, filtered, and concentrated to give a crude product, which was purified by silica gel chromatography (hexane/EtOAc = 2/1) to afford BmP-3,5-^*i*^Pr (0.15 g, 12%): white solid; mp 78.7–79.0 °C; ^1^H NMR (400 MHz, CDCl_3_) δ 7.93 (d, *J* = 9.0 Hz, 1H), 7.85 (d, *J* = 8.0 Hz, 1H), 7.69–7.63 (m, 1H), 7.47 (d, *J* = 5.3 Hz, 1H), 7.37–7.21 (m, 5H), 7.19–7.03 (m, 8H), 5.44 (s, 1H), 5.02 (s, 1H), 2.86 (octet, *J* = 7.0 Hz, 4H), 1.23–1.15 (m, 24H); ^13^C­{^1^H} NMR (100 MHz, CDCl_3_, peaks are very complicated due to the coupling between C and P and/or diastereotopic carbons) δ 154.0 (d, *J* = 15 Hz), 152.6, 149.0 (dd, *J* = 7.3, 11.6 Hz), 136.1 (d, *J* = 5.1 Hz), 135.1 (dd, *J* = 4.4, 8.4 Hz), 134.0, 133.4, 131.2, 129.7, 129.5, 129.4, 129.3, 129.1, 129.1, 128.6, 128.4, 128.3. 127.9, 127.8, 127.7, 127.3, 125.8, 125.6, 124.2, 124.2, 124.1, 123.9, 117.7, 111.4, 110.8, 34.23, and 34.19 (diastereotopic benzylic C), 24.11, 24.08, and 24.04 (diastereotopic terminal −CH_3_); ^31^P NMR (162 MHz, CDCl_3_) δ – 15.2; HRMS (APCI, positive) *m*/*z*: [M + H]^+^ calcd. for C_44_H_48_O_2_P^+^ 639.3386; found 639.3383.

**2-((3-Phenylprop-2-yn-1-yl)­oxy)­tetrahydro-2*H*-pyran (S1)** ^19^

**2-((3-(*p*-Tolyl)­prop-2-yn-1-yl)­oxy)­tetrahydro-2*H*-pyran (S2)** ^19^

**2-((3-(4-Methoxyphenyl)­prop-2-yn-1-yl)­oxy)­tetrahydro-2*H*-pyran (S3)** ^20^

**2-((3-(4-Fluorophenyl)­prop-2-yn-1-yl)­oxy)­tetrahydro-2H-pyran (S4)**: 1-Fluoro-4-iodobenzene (15 mmol, 1.7 mL, *d* = 1.92) and Et_3_N (30 mL) were added to a round bottomed flask and the flask was evacuated and backfilled with argon three times. CuI (0.30 mmol, 57 mg, 2 mol %) and Pd­(PPh_3_)­Cl_2_ (0.15 mmol, 105 mg, 1 mol %) were added, then THP-protected propargyl alcohol (15 mmol, 2.1 g, 2.1 mL, *d* = 1.00) was added dropwise. The reaction mixture was stirred at rt. After 24 h, the resulting mixture was filtered through a pad of Celite, washed with EtOAc and concentrated. The crude product was purified by silica gel chromatography (hexane/EtOAc = 10/1) to afford **S4** (10 mmol, 2.42 g, 69%) as a colorless oil: ^1^H NMR (400 MHz, CDCl_3_) δ 7.46–7.37 (m, 2H), 6.98 (tt, *J* = 8.8, 2.3 Hz, 2H), 4.87 (t, *J* = 3.4 Hz, 1H), 4.49 (d, *J* = 15.8 Hz, 1H), 4.42 (d, *J* = 15.8 Hz, 1H), 3.92–3.82 (m, 1H), 3.59–3.51 (m, 1H), 1.91–1.47 (m, 6H); ^13^C­{^1^H} NMR (100 MHz, CDCl_3_) δ 163.7, 161.2, 133.7 (d, *J* = 8.0 Hz), 118.8 (d, *J* = 3.6 Hz), 115.4 (d, *J* = 21.8 Hz), 96.8, 84.7 (d, *J* = 15.3 Hz), 61.9, 54.6, 30.2, 25.3, 19.0; ^19^F NMR (375 MHz, CDCl_3_) δ – 110.7; HRMS (APCI, positive) *m*/*z*: [M + H]^+^ calcd for C_14_H_16_FO_2_^+^ 235.1129; found 235.1122.

**2-((3-([1,1’-Biphenyl]-4-yl)­prop-2-yn-1-yl)­oxy)­tetrahydro-2H-pyran (S5)**: 4-Bromobiphenyl (15 mmol, 3.50 g) and Et_3_N (30 mL) were added to a round bottomed flask and the flask was evacuated and backfilled with argon three times. CuI (0.30 mmol, 57 mg, 2 mol %) and Pd­(PPh_3_)­Cl_2_ (0.15 mmol, 105 mg, 1 mol %) were added, then THP-protected propargyl alcohol (15 mmol, 2.1 g, 2.1 mL, *d* = 1.00) was added dropwise. The reaction mixture was stirred under reflux condition. After 24 h, the resulting mixture was filtered through a pad of Celite, washed with EtOAc and concentrated. The crude product was purified by silica gel chromatography (hexane/EtOAc = 10/1) to afford **S5** (5.7 mmol, 1.66 g, 38%) as a white solid: mp 53.9–54.2 °C; ^1^H NMR (400 MHz, CDCl_3_) δ 7.64–7.50 (m, 6H), 7.49–7.41 (m, 2H), 7.36 (tt, *J* = 7.4, 1.5 Hz, 1H), 4.98–4.90 (m, 1H), 4.60–4.45 (m, 2H), 3.97–3.86 (m, 1H), 3.64–3.54 (m, 1H), 1.97–1.50 (m, 6H); ^13^C­{^1^H} NMR (100 MHz, CDCl_3_) δ 141.1, 140.3, 132.2, 128.8, 127.6, 127.0, 126.9, 121.6, 96.8, 85.8, 85.6, 62.0, 54.8, 30.3, 25.4, 19.0; HRMS (APCI, positive) *m*/*z*: [M + H]^+^ calcd for C_20_H_21_O_2_^+^ 293.1536; found 293.1533.

**2-((3-(2-Methoxyphenyl)­prop-2-yn-1-yl)­oxy)­tetrahydro-2*H*-pyran (S6)** ^19^

**2-((3-(Naphthalen-2-yl)­prop-2-yn-1-yl)­oxy)­tetrahydro-2H-pyran (S7)**: 2-Bromonaphthalene (15 mmol, 3.11 g) and Et_3_N (30 mL) were added to a round bottomed flask and the flask was evacuated and backfilled with argon three times. CuI (0.30 mmol, 57 mg, 2 mol %) and Pd­(PPh_3_)­Cl_2_ (0.15 mmol, 105 mg, 1 mol %) were added, then THP-protected propargyl alcohol (15 mmol, 2.1 g, 2.1 mL, *d* = 1.00) was added dropwise. The reaction mixture was stirred under reflux condition. After 24 h, the resulting mixture was filtered through a pad of Celite, washed with EtOAc and concentrated. The crude product was purified by silica gel chromatography (hexane/EtOAc = 10/1) to afford **S6** (6.2 mmol, 1.65 g, 41%) as a colorless oil: ^1^H NMR (400 MHz, CDCl_3_) δ 7.96 (s, 1H), 7.79–7.68 (m, 3H), 7.52–7.38 (m, 3H), 4.93 (t, *J* = 3.3 Hz, 1H), 4.57 (d, *J* = 15.8 Hz, 1H), 4.49 (d, *J* = 15.8 Hz, 1H), 3.94–3.84 (m, 1H), 3.60–3.51 (m, 1H), 1.94–1.43 (m, 6H); ^13^C­{^1^H} NMR (100 MHz, CDCl_3_) δ 132.73, 132.68, 131.6, 128.3, 127.8, 127.6, 126.5, 126.3, 119.9, 96.–786.0, 85.4, 61.8, 54.7, 30.2, 25.3, 18.9 (One carbon atom was not found probably due to overlapping); HRMS (APCI, positive) *m*/*z*: [M + H]^+^ calcd for C_18_H_19_O_2_^+^ 267.1380; found 267.1370.

**2-((3-(Naphthalen-1-yl)­prop-2-yn-1-yl)­oxy)­tetrahydro-2*H*-pyran (S8)** ^21^

**2-((3-(Furan-2-yl)­prop-2-yn-1-yl)­oxy)­tetrahydro-2H-pyran (S9)**: 2-Iodofuran (8.0 mmol, 1.55 g) and Et_3_N (16 mL) were added to a round bottomed flask and the flask was evacuated and backfilled with argon three times. CuI (0.16 mmol, 30 mg, 2 mol %) and Pd­(PPh_3_)­Cl_2_ (0.08 mmol, 56 mg, 1 mol %) were added, then THP-protected propargyl alcohol (8.0 mmol, 1.12 g, 1.12 mL, *d* = 1.00) was added dropwise. The reaction mixture was stirred at rt. After 24 h, the resulting mixture was filtered through a pad of Celite, washed with EtOAc and concentrated. The crude product was purified by silica gel chromatography (hexane/EtOAc = 10/1) to afford **S9** (5.88 mmol, 1.21 g, 73%) as a yellow oil: ^1^H NMR (400 MHz, CDCl_3_) δ 7.39–7.36 (m, 1H), 6.59 (d, *J* = 3.5 Hz, 1H), 6.39–6.35 (m, 1H), 4.87 (t, *J* = 3.3 Hz, 1H), 4.49 (dd, *J* = 22.9, 16.1 Hz, 2H), 3.91–3.82 (m, 1H), 3.60–3.52 (m, 1H), 1.95–1.45 (m, 6H); ^13^C­{^1^H} NMR (100 MHz, CDCl_3_) δ 143.5, 136.5, 115.6, 110.7, 96.8, 89.7, 76.0, 61.9, 54.5, 30.1, 25.3, 18.9; HRMS (APCI, positive) *m*/*z*: [M + H]^+^ calcd for C_12_H_15_O_3_^+^ 207.1016; found 207.1016.

**2-((3-(Thiophen-2-yl)­prop-2-yn-1-yl)­oxy)­tetrahydro-2*H*-pyran (S10)** ^22^

**6-Methyl-3-phenyl-6-((tetrahydro-2H-pyran-2-yl)­oxy)­hept-4-yn-3-ol (S11)**: To a solution of THP-protected 2-methyl-3-butyn-2-ol (10 mmol, 1.68 g) in THF (30 mL) at – 8 °C, ^*n*^BuLi in hexane (12 mmol, 7.7 mL, 1.55 M) was added dropwise, then warmed to 0 °C and stirred for 30 min. A solution of propiophenone (15 mmol, 2.02 g, 2.0 mL, *d* = 1.01) in THF (15 mL) was added, then warmed to rt and stirred for 16 h. The reaction mixture was quenched with sat. NH_4_Cl aq. at 0 °C. The aqueous layer was extracted with EtOAc (20 mL × 2) and the combined organic layers were dried over anh. Na_2_SO_4_, filtered, and concentrated. The crude product was purified by silica gel chromatography (hexane/EtOAc = 5/1) to afford the diastereomeric mixture of **S11** (7.2 mmol, 2.19 g, 72%) as a yellow oil: ^1^H NMR (400 MHz, CDCl_3_) δ 7.64–7.55 (m, 2H), 7.38–7.30 (m, 2H), 7.30–7.23 (m, 1H), 5.12–5.04 (m, 1H), 3.99–3.89 (m, 1H), 3.50–3.41 (m, 1H), 2.89 (d, *J* = 18 Hz, 1H), 2.06–1.76 (m, 3H), 1.76–1.42 (m, 11H), 0.94 (t, *J* = 7.4 Hz, 3H); ^13^C­{^1^H} NMR (100 MHz, CDCl_3_) δ 144.7, 144.6, 128.0, 127.5, 125.5, 96.2, 96.1, 88.6, 88.5, 86.2, 86.1, 73.7, 71.1, 63.3, 63.3, 38.4, 32.0, 31.9, 30.5, 30.5, 30.0, 25.3, 20.5, 20.4, 9.1; HRMS (APCI, positive) *m*/*z*: [M–OH]^+^ calcd for C_19_H_25_O_2_^+^ 285.1849; found 285.1849.

**6-Methyl-3-phenylhepta-4,5-dien-3-ol (S12)**: To a suspension of LiAlH_4_ (9.0 mmol, 0.34 g) in Et_2_O (18 mL); a solution of **S11** (7.2 mmol, 2.19 g) in Et_2_O (7 mL) was slowly added. The reaction mixture was stirred for 4 h under reflux condition, quenched with minimal amount of H_2_O at 0 °C, filtered through a pad of Celite and concentrated. The crude product was purified by silica gel chromatography (hexane/EtOAc = 20/1) to afford **S12** (4.4 mmol, 0.89 g, 61%) as a colorless oil: ^1^H NMR (400 MHz, CDCl_3_) δ 7.48–7.43 (m, 2H), 7.36–7.29 (m, 2H), 7.25–7.19 (m, 1H), 5.36 (hept, *J* = 2.8 Hz, 1H), 2.10 (s, 1H), 1.97–1.82 (m, 2H), 1.76 (d, *J* = 2.9 Hz, 3H), 1.72 (d, *J* = 3.9 Hz, 3H), 0.84 (t, *J* = 7.4 Hz, 3H); ^13^C­{^1^H} NMR (100 MHz, CDCl_3_) δ 198.4, 146.5, 127.9, 126.5, 125.3, 100.7, 98.3, 75.5, 35.4, 20.7, 20.5, 8.1; HRMS (APCI, positive) *m*/*z*: [M–OH]^+^ calcd for C_14_H_17_^+^ 185.1325; found 185.1317.

**(3-Methoxy-6-methylhepta-4,5-dien-3-yl)­benzene (S13)**: To a suspension of NaH (3.0 mmol, 0.12 g, 60% oil) in THF (15 mL), **S12** (1.5 mmol, 0.30 g) was added dropwise at 0 °C; then resulting mixture was stirred at rt for 1 h. To the resulting mixture was added MeI (6.0 mmol, 0.84 g, 0.37 mL, *d* = 2.28). After 1 h, the reaction mixture was quenched with sat. NH_4_Cl aq. at 0 °C. The aqueous layer was extracted with EtOAc (10 mL x 2). The combined organic layers were dried over anh. Na_2_SO_4_, filtered, and concentrated. The crude product was purified by silica gel chromatography (hexane) to afford **S13** (1.4 mmol, 0.31 g, 95%) as a colorless oil: ^1^H NMR (400 MHz, CDCl_3_) δ 7.45–7.39 (m, 2H), 7.32 (tt, *J* = 7.5, 1.7 Hz, 2H), 7.22 (tt, *J* = 7.5, 1.7 Hz, 1H), 5.14 (hept, *J* = 3.0 Hz, 1H), 3.25 (s, 3H), 1.93 (dq, *J* = 14.3, 7.4 Hz, 1H), 1.83–1.71 (m, 7H), 0.66 (t, *J* = 7.4 Hz, 3H); ^13^C­{^1^H} NMR (100 MHz, CDCl_3_) δ 202.4, 144.2, 127.7, 126.6, 126.5, 97.5, 93.5, 81.8, 50.4, 31.2, 20.4, 20.3, 7.9; HRMS (APCI, positive) *m*/*z*: [M + H]^+^ calcd for C_15_H_21_O^+^ 217.1587; found 217.1585.

**(Z)-2-Hydroxy-2-methyl-5-phenylhept-4-en-3-one ((Z)-1a)**: To a solution of **S1** (20 mmol, 4.33 g) in THF (40 mL) at −78 °C, the solution of ^*n*^BuLi in hexane (24 mmol, 15.5 mL, 1.55 M) was added dropwise. After stirring at −78 °C for 30 min, bromoethane (24 mmol, 2.63 g, 1.8 mL, *d* = 1.46) was slowly added, then stirred at rt for 1 h. To the resulting mixture at −78 °C, ^*n*^BuLi in hexane (24 mmol, 15.5 mL, 1.55 M) was farther added dropwise and stirred for 30 min, then a solution of acetone (24 mmol, 1.42 g, 1.8 mL, *d* = 0.79) in THF (40 mL) was slowly added. After stirring for 1 h, the reaction mixture was allowed to warm to rt and stirred for 1 h, then quenched with sat. NH_4_Cl aq. at 0 °C. The aquous layer was extracted with EtOAc (20 mL × 3) and the combined organic layers were dried over anh. Na_2_SO_4_, filtered, and concentrated. The crude product was dissolved in a solution of THF (20 mL), H_2_O (20 mL) and AcOH (40 mmol, 2.3 mL, *d* = 1.05) and stirred under reflux condition for 16 h. The aquous layer was extracted with EtOAc (20 mL × 2) and the combined organic layers were washed with sat. NaHCO_3_ aq., dried over anh. Na_2_SO_4_, filtered, and concentrated. The crude product was purified by silica gel chromatography (hexane/EtOAc = 5/1) to afford (*Z*)-**1a** (10 mmol, 2.21 g, 51%): orange oil; ^1^H NMR (400 MHz, CDCl_3_) δ 7.39–7.31 (m, 3H), 7.13–7.08 (m, 2H), 6.40 (t, *J* = 1.4 Hz, 1H), 3.93 (s, 1H), 2.51 (qd, *J* = 7.4, 1.4 Hz, 2H), 1.41 (s, 6H), 1.07 (t, *J* = 7.4 Hz, 3H); ^13^C­{^1^H} NMR (100 MHz, CDCl_3_) δ 202.5, 163.9, 140.2, 128.0, 127.7, 126.6, 117.0, 75.4, 34.1, 26.6, 12.1; HRMS (APCI, positive) *m*/*z*: [M + H]^+^ calcd. for C_14_H_19_O_2_^+^ 219.1380; found 219.1377.

**(*E*)-2-Hydroxy-2-methyl-5-phenylhept-4-en-3-one ((*E*)-1a)**: The product was obtained by the isomerization of (*Z*)-**1a** (10 mmol, 2.21 g), which was treated with catalytic amount of HCl aq. in THF (20 mL) under reflux condition for 16 h and purified by silica gel chromatography (hexane/EtOAc = 5/1) to afford (*E*)-**1a** (5.8 mmol, 1.27 g, 58%). The product was also afforded by synthesis of (*Z*)-**1a** as a minor product (2.19 mmol, 0.48 g, 11%): orange oil; ^1^H NMR (400 MHz, CDCl_3_) δ 7.50–7.44 (m, 2H), 7.44–7.38 (m, 3H), 6.55 (s, 1H), 4.19 (s, 1H), 3.09 (q, *J* = 7.5 Hz, 2H), 1.44 (s, 6H), 1.10 (t, *J* = 7.5 Hz, 3H); ^13^C­{^1^H} NMR (100 MHz, CDCl_3_) δ 203.3, 164.8, 141.2, 129.4, 128.6, 126.7, 117.6, 75.8, 26.7, 25.2, 13.4; HRMS (APCI, positive) *m*/*z*: [M + H]^+^ calcd. for C_14_H_19_O_2_^+^ 219.1380; found 219.1373.

**(*E*)-2-Hydroxy-2-methyl-5-(p-tolyl)­hept-4-en-3-one ((*E*)-1b)**: To a solution of **S2** (3 mmol, 0.69 g) in THF (6 mL) at −78 °C, the solution of ^*n*^BuLi in hexane (3.6 mmol, 2.3 mL, 1.55 M) was added dropwise. After stirring at −78 °C for 30 min, bromoethane (3.6 mmol, 0.39 g, 0.27 mL, *d* = 1.46) was slowly added, then stirred at rt for 1 h. To the resulting mixture at −78 °C, ^*n*^BuLi in hexane (3.6 mmol, 2.3 mL, 1.55 M) was farther added dropwise and stirred for 30 min, then a solution of acetone (3.6 mmol, 0.21 g, 0.26 mL, *d* = 0.79) in THF (6 mL) was slowly added. After stirring for 1 h, the reaction mixture was allowed to warm to rt and stirred for 1 h, then quenched with sat. NH_4_Cl aq. at 0 °C. The aquous layer was extracted with EtOAc (5 mL × 3) and the combined organic layers were dried over anh. Na_2_SO_4_, filtered, and concentrated. The crude product was dissolved in a solution of THF (3 mL), H_2_O (3 mL), and AcOH (6 mmol, 0.36 g, 0.34 mL, *d* = 1.05) and stirred under reflux condition for 16 h. The aquous layer was extracted with EtOAc (5 mL × 2) and the combined organic layers were washed with sat. NaHCO_3_ aq., dried over anh. Na_2_SO_4_, filtered, and concentrated. The crude product was purified by silica gel chromatography (hexane/EtOAc = 5/1) to afford (*E*)-**1b** (0.12 mmol, 28.3 mg, 4%). The product was also obtained by the isomerization of (*Z*)-**1b** (1.3 mmol, 0.31 g), which was treated with catalytic amount of HCl aq. in THF (6 mL) under reflux condition for 16 h and purified by silica gel chromatography (hexane/EtOAc = 5/1) to afford (*E*)-**1b** (1.1 mmol, 0.25 g, 83%): orange solid; mp 26.2–26.8 °C; ^1^H NMR (400 MHz, CDCl_3_) δ 7.39 (dt, *J* = 8.3, 2.0 Hz, 2H), 7.24–7.19 (m, 2H), 6.55 (s, 1H), 4.23 (s, 1H), 3.08 (q, *J* = 7.5 Hz, 2H), 2.39 (s, 3H), 1.44 (s, 6H), 1.10 (t, *J* = 7.5 Hz, 3H); ^13^C­{^1^H} NMR (100 MHz, CDCl_3_) δ 203.2, 164.9, 139.8, 138.2, 129.4, 126.7, 116.8, 75.8, 26.8, 25.1, 21.2, 13.6; HRMS (APCI, positive) *m*/*z*: [M + H]^+^ calcd. for C_15_H_21_O_2_^+^ 233.1536; found 233.1536.

**(*E*)-2-Hydroxy-5-(4-methoxyphenyl)-2-methylhept-4-en-3-one ((*E*)-1c)**: To a solution of **S3** (3 mmol, 0.74 g) in THF (6 mL) at −78 °C, the solution of ^*n*^BuLi in hexane (3.6 mmol, 2.3 mL, 1.55 M) was added dropwise. After stirring at −78 °C for 30 min, bromoethane (3.6 mmol, 0.39 g, 0.27 mL, *d* = 1.46) was slowly added, then stirred at rt for 1 h. To the resulting mixture at −78 °C, ^*n*^BuLi in hexane (3.6 mmol, 2.3 mL, 1.55 M) was farther added dropwise and stirred for 30 min, then a solution of acetone (3.6 mmol, 0.21 g, 0.26 mL, *d* = 0.79) in THF (6 mL) was slowly added. After stirring for 1 h, the reaction mixture was allowed to warm to rt and stirred for 1 h, then quenched with sat. NH_4_Cl aq. at 0 °C. The aquous layer was extracted with EtOAc (5 mL × 3) and the combined organic layers were dried over anh. Na_2_SO_4_, filtered, and concentrated. The crude product was dissolved in a solution of THF (3 mL), H_2_O (3 mL), and AcOH (6 mmol, 0.36 g, 0.34 mL, *d* = 1.05) and stirred under reflux condition for 16 h. The aquous layer was extracted with EtOAc (5 mL × 2) and the combined organic layers were washed with sat. NaHCO_3_ aq., dried over anh. Na_2_SO_4_, filtered, and concentrated. The crude product was purified by silica gel chromatography (hexane/EtOAc = 5/1) to afford (*E*)-**1c** (0.26 mmol, 64.1 mg, 9%). The product was also obtained by the isomerization of (*Z*)-**1c** (1.2 mmol, 0.29 g), which was treated with catalytic amount of HCl aq. in THF (6 mL) under reflux condition for 16 h and purified by silica gel chromatography (hexane/EtOAc = 5/1) to afford (*E*)-**1c** (0.29 mmol, 71.1 mg, 24%): yellow oil; ^1^H NMR (400 MHz, CDCl_3_) δ 7.47 (dt, *J* = 9.0, 2.6 Hz, 2H), 6.93 (dt, *J* = 9.0, 2.6 Hz, 2H), 6.55 (s, 1H), 4.25 (s, 1H), 3.85 (s, 3H), 3.09 (q, *J* = 7.4 Hz, 2H), 1.44 (s, 6H), 1.11 (t, *J* = 7.4 Hz, 3H); ^13^C­{^1^H} NMR (100 MHz, CDCl_3_) δ 203.0, 164.5, 160.9, 133.2, 128.3, 115.7, 114.1, 75.7, 55.4, 26.9, 24.9, 13.7; HRMS (APCI, positive) *m*/*z*: [M + H]^+^ calcd. for C_15_H_21_O_3_^+^ 249.1585; found 249.1582.

**(*E*)-5-(4-Fluorophenyl)-2-hydroxy-2-methylhept-4-en-3-one ((*E*)-1d)**: To a solution of **S4** (3 mmol, 0.70 g) in THF (6 mL) at −78 °C, the solution of ^*n*^BuLi in hexane (3.6 mmol, 2.3 mL, 1.55 M) was added dropwise. After stirring at −78 °C for 30 min, bromoethane (3.6 mmol, 0.39 g, 0.27 mL, *d* = 1.46) was slowly added, then stirred at rt for 1 h. To the resulting mixture at −78 °C, ^*n*^BuLi in hexane (3.6 mmol, 2.3 mL, 1.55 M) was farther added dropwise and stirred for 30 min, then a solution of acetone (3.6 mmol, 0.21 g, 0.26 mL, *d* = 0.79) in THF (6 mL) was slowly added. After stirring for 1 h, the reaction mixture was allowed to warm to rt and stirred for 1 h, then quenched with sat. NH_4_Cl aq. at 0 °C. The aqueous layer was extracted with EtOAc (5 mL × 3) and the combined organic layers were dried over anh. Na_2_SO_4_, filtered, and concentrated. The crude product was dissolved in a solution of THF (3 mL), H_2_O (3 mL), and AcOH (6 mmol, 0.36 g, 0.34 mL, *d* = 1.05) and stirred under reflux condition for 16 h. The aquous layer was extracted with EtOAc (5 mL × 2) and the combined organic layers were washed with sat. NaHCO_3_ aq., dried over anh. Na_2_SO_4_, filtered, and concentrated. The crude product was purified by silica gel chromatography (hexane/EtOAc = 5/1) to afford (*E*)-**1d** (0.37 mmol, 87.3 mg, 12%). The product was also obtained by the isomerization of (*Z*)-**1d** (1.1 mmol, 0.25 g), which was treated with catalytic amount of HCl aq. in THF (6 mL) under reflux condition for 16 h and purified by silica gel chromatography (hexane/EtOAc = 5/1) to afford (*E*)-**1d** (0.54 mmol, 0.13 g, 51%): orange oil; ^1^H NMR (400 MHz, CDCl_3_) δ 7.49–7.43 (m, 2H), 7.13–7.07 (m, 2H), 6.52 (s, 1H), 4.13 (s, 1H), 3.06 (q, *J* = 7.5 Hz, 2H), 1.44 (s, 6H), 1.09 (t, *J* = 7.5 Hz, 3H); ^13^C­{^1^H} NMR (100 MHz, CDCl_3_) δ 203.2, 163.6, 163.5 (d, *J* = 249.5 Hz), 137.2 (d, *J* = 2.9 Hz), 128.7 (d, *J* = 8.7 Hz), 117.5, 115.7 (d, *J* = 22.2 Hz), 75.8, 26.8, 25.2, 13.4; ^19^F NMR (375 MHz, CDCl_3_) δ – 111.5; HRMS (APCI, positive) *m*/*z*: [M + H]^+^ calcd. for C_14_H_18_FO_2_^+^ 237.1285; found 237.1288.

**(*E*)-5-([1,1’-Biphenyl]-4-yl)-2-hydroxy-2-methylhept-4-en-3-one ((*E*)-1e)**: To a solution of **S5** (3 mmol, 0.88 g) in THF (6 mL) at −78 °C, the solution of ^*n*^BuLi in hexane (3.6 mmol, 2.3 mL, 1.55 M) was added dropwise. After stirring at −78 °C for 30 min, bromoethane (3.6 mmol, 0.39 g, 0.27 mL, *d* = 1.46) was slowly added, then stirred at rt for 1 h. To the resulting mixture at −78 °C, ^*n*^BuLi in hexane (3.6 mmol, 2.3 mL, 1.55 M) was farther added dropwise and stirred for 30 min, then a solution of acetone (3.6 mmol, 0.21 g, 0.26 mL, *d* = 0.79) in THF (6 mL) was slowly added. After stirring for 1 h, the reaction mixture was allowed to warm to rt and stirred for 1 h, then quenched with sat. NH_4_Cl aq. at 0 °C. The aqueous layer was extracted with EtOAc (5 mL × 3) and the combined organic layers were dried over anh. Na_2_SO_4_, filtered, and concentrated. The crude product was dissolved in a solution of THF (3 mL), H_2_O (3 mL), and AcOH (6 mmol, 0.36 g, 0.34 mL, *d* = 1.05) and stirred under reflux condition for 16 h. The aquous layer was extracted with EtOAc (5 mL × 2) and the combined organic layers were washed with sat. NaHCO_3_ aq., dried over anh. Na_2_SO_4_, filtered, and concentrated. The crude product was purified by silica gel chromatography (hexane/EtOAc = 5/1) to afford (*E*)-**1e** (0.43 mmol, 0.13 mg, 14%). The product was also obtained by the isomerization of (*Z*)-**1e** (1.4 mmol, 0.40 g), which was stored with catalytic amount of HCl from residual CHCl_3_ under rt for 2 weeks and purified by silica gel chromatography (hexane/EtOAc = 5/1) to afford (*E*)-**1e** (0.98 mmol, 0.29 g, 72%): yellow solid; mp 69.3–69.6 °C; ^1^H NMR (400 MHz, CDCl_3_) δ 7.67–7.59 (m, 4H), 7.56 (dt, *J* = 8.5, 2.0 Hz, 2H), 7.50–7.43 (m, 2H), 7.41–7.35 (m, 1H), 6.63 (s, 1H), 4.20 (s, 1H), 3.13 (q, *J* = 7.5 Hz, 2H), 1.46 (s, 6H), 1.14 (t, *J* = 7.5 Hz, 3H); ^13^C­{^1^H} NMR (100 MHz, CDCl_3_) δ 203.3, 164.3, 142.4, 140.1, 140.0, 128.9, 127.8, 127.3, 127.3, 127.0, 117.4, 75.8, 26.8, 25.1, 13.6; HRMS (APCI, positive) *m*/*z*: [M + H]^+^ calcd. for C_20_H_23_O_2_^+^ 295.1693; found 295.1692.

**(*E*)-2-Hydroxy-5-(2-methoxyphenyl)-2-methylhept-4-en-3-one ((*E*)-1f)**: To a solution of **S6** (5 mmol, 1.23 g, 1.1 mL, *d* = 1.12) in THF (10 mL) at −78 °C, the solution of ^*n*^BuLi in hexane (5.0 mmol, 3.2 mL, 1.54 M) was added dropwise. After stirring at −78 °C for 30 min, bromoethane (5.0 mmol, 0.54 g, 0.37 mL, *d* = 1.46) was slowly added, then stirred at rt for 1 h. To the resulting mixture at −78 °C, ^*n*^BuLi in hexane (5.0 mmol, 3.2 mL, 1.54 M) was further added dropwise and stirred for 30 min, then a solution of acetone (5.0 mmol, 0.3 g, 0.37 mL, *d* = 0.79) in THF (5 mL) was slowly added. After stirring for 1 h, the reaction mixture was allowed to warm to rt and stirred for 1 h, then quenched with sat. NH_4_Cl aq. at 0 °C. The aqueous layer was extracted with EtOAc (5 mL × 3) and the combined organic layers were dried over anh. Na_2_SO_4_, filtered, and concentrated. The crude product was dissolved in a solution of THF (5 mL), H_2_O (5 mL), and AcOH (10 mmol, 0.60 g, 0.57 mL, *d* = 1.05) and stirred under reflux condition for 16 h. The aquous layer was extracted with EtOAc (5 mL × 2) and the combined organic layers were washed with sat. NaHCO_3_ aq., dried over anh. Na_2_SO_4_, filtered, and concentrated. The crude product was purified by silica gel chromatography (hexane/EtOAc = 5/1) to afford (*E*)-**1f** (0.68 mmol, 0.17 g, 14%). The product was also obtained by the isomerization of (*Z*)-**1f** (2.8 mmol, 0.70 g), which was treated with catalytic amount of HCl aq. in THF (3 mL) under reflux condition for 16 h and purified by silica gel chromatography (hexane/EtOAc = 5/1) to afford (*E*)-**1f** (0.97 mmol, 0.24 g, 35%): pale yellow oil; ^1^H NMR (400 MHz, CDCl_3_) δ 7.34–7.30 (m, 1H), 7.12 (dd, *J* = 7.5, 1.8 Hz, 1H), 7.00–6.92 (m, 2H), 6.37 (s, 1H), 4.23 (s, 1H), 3.82 (s, 3H), 3.07–2.99 (m, 2H), 1.41 (s, 6H), 0.98 (t, *J* = 7.5 Hz, 3H); ^13^C­{^1^H} NMR (100 MHz, CDCl_3_) δ 203.5, 165.2, 156.5, 131.5, 129.7, 129.3, 120.5, 119.8, 111.0, 75.7, 55.4, 26.7, 26.2, 12.7; HRMS (APCI, positive) *m*/*z*: [M + H]^+^ calcd. for C_15_H_21_O_3_^+^ 249.1485; found 249.1481.

**(*E*)-2-Hydroxy-2-methyl-5-(naphthalen-2-yl)­hept-4-en-3-one ((*E*)-1g)**: To a solution of **S7** (3 mmol, 0.80 g) in THF (6 mL) at −78 °C, the solution of ^*n*^BuLi in hexane (3.6 mmol, 2.3 mL, 1.55 M) was added dropwise. After stirring at −78 °C for 30 min, bromoethane (3.6 mmol, 0.39 g, 0.27 mL, *d* = 1.46) was slowly added, then stirred at rt for 1 h. To the resulting mixture at −78 °C, ^*n*^BuLi in hexane (3.6 mmol, 2.3 mL, 1.55 M) was farther added dropwise and stirred for 30 min, then a solution of acetone (3.6 mmol, 0.21 g, 0.26 mL, *d* = 0.79) in THF (6 mL) was slowly added. After stirring for 1 h, the reaction mixture was allowed to warm to rt and stirred for 1 h, then quenched with sat. NH_4_Cl aq. at 0 °C. The aquous layer was extracted with EtOAc (5 mL × 3) and the combined organic layers were dried over anh. Na_2_SO_4_, filtered, and concentrated. The crude product was dissolved in a solution of THF (3 mL), H_2_O (3 mL), and AcOH (6 mmol, 0.36 g, 0.34 mL, *d* = 1.05) and stirred under reflux condition for 16 h. The aqueous layer was extracted with EtOAc (5 mL × 2) and the combined organic layers were washed with sat. NaHCO_3_ aq., dried over anh. Na_2_SO_4_, filtered, and concentrated. The crude product was purified by silica gel chromatography (hexane/EtOAc = 5/1) to afford (*E*)-**1g** (0.35 mmol, 94.6 mg, 12%). The product was also obtained by the isomerization of (*Z*)-**1g** (1.1 mmol, 0.28 g), which was treated with catalytic amount of HCl aq. in THF (6 mL) under reflux condition for 16 h and purified by silica gel chromatography (hexane/EtOAc = 5/1) to afford (*E*)-**1g** (0.50 mmol, 0.13 g, 47%): yellow solid; mp 36.2–36.8 °C; ^1^H NMR (400 MHz, CDCl_3_) δ 7.95 (d, *J* = 1.5 Hz, 1H), 7.93–7.82 (m, 3H), 7.60–7.49 (m, 3H), 6.69 (s, 1H), 4.21 (s, 1H), 3.21 (q, *J* = 7.5 Hz, 2H), 1.48 (s, 6H), 1.15 (t, *J* = 7.5 Hz, 3H); ^13^C­{^1^H} NMR (100 MHz, CDCl_3_) δ 203.3, 164.7, 138.5, 133.7, 133.1, 128.5, 128.4, 127.6, 126.9, 126.7, 126.5, 124.3, 118.0, 75.9, 26.9, 25.2, 13.6; HRMS (APCI, positive) *m*/*z*: [M + H]^+^ calcd. for C_18_H_21_O_2_^+^ 269.1536; found 269.1532.

**(*E)*-2-Hydroxy-2-methyl-5-(naphthalen-1-yl)­hept-4-en-3-one ((*E*)-1h)**: To a solution of **S8** (5 mmol, 1.33 g, 1.2 mL, *d* = 1.13) in THF (10 mL) at −78 °C, the solution of ^*n*^BuLi in hexane (5.0 mmol, 3.2 mL, 1.54 M) was added dropwise. After stirring at −78 °C for 30 min, bromoethane (5.0 mmol, 0.37 mL, *d* = 1.46) was slowly added, then stirred at rt for 1 h. To the resulting mixture at −78 °C, ^*n*^BuLi in hexane (5.0 mmol, 3.2 mL, 1.54 M) was farther added dropwise and stirred for 30 min, then a solution of acetone (5.0 mmol, 0.3 g, 0.37 mL, *d* = 0.79) in THF (5 mL) was slowly added. After stirring for 1 h, the reaction mixture was allowed to warm to rt and stirred for 1 h, then quenched with sat. NH_4_Cl aq. at 0 °C. The aqueous layer was extracted with EtOAc (5 mL × 3) and the combined organic layers were dried over anh. Na_2_SO_4_, filtered, and concentrated. The crude product was dissolved in a solution of THF (5 mL), H_2_O (5 mL), and AcOH (10 mmol, 0.60 g, 0.57 mL, *d* = 1.05) and stirred under reflux condition for 16 h. The aquous layer was extracted with EtOAc (5 mL × 2) and the combined organic layers were washed with sat. NaHCO_3_ aq., dried over anh. Na_2_SO_4_, filtered, and concentrated. The crude product was purified by silica gel chromatography (hexane/EtOAc = 10/1) to afford (*E*)-**1h** (0.84 mmol, 0.23 g, 17%). The product was also obtained by the isomerization of (*Z*)-**1h** (2.1 mmol, 0.55 g), which was treated with catalytic amount of HCl aq. in THF (2 mL) under reflux condition for 16 h and purified by silica gel chromatography (hexane/EtOAc = 10/1) to afford (*E*)-**1h** (0.30 mmol, 81 mg, 15%): yellow oil; ^1^H NMR (400 MHz, CDCl_3_) δ 7.91–7.81 (m, 3H), 7.55–7.44 (m, 3H), 7.28 (dd, *J* = 7.0, 1.0 Hz, 1H), 6.45 (s, 1H), 4.20 (s, 1H), 3.13 (q, *J* = 7.5 Hz, 2H), 1.41 (s, 6H), 1.04 (t, *J* = 7.5 Hz, 3H); ^13^C­{^1^H} NMR (100 MHz, CDCl_3_) δ 203.5, 165.5, 140.4, 133.7, 130.6, 128.5, 128.4, 126.5, 126.1, 125.2, 125.0, 124.6, 121.1, 75.8, 28.2, 26.6, 12.7; HRMS (APCI, positive) *m*/*z*: [M + H]^+^ calcd. for C_18_H_21_O_2_^+^ 269.1536; found 269.1533.

**(*E*)-5-(Furan-2-yl)-2-hydroxy-2-methylhept-4-en-3-one ((*E*)-1i)**: To a solution of **S9** (5.9 mmol, 1.21 g) in THF (12 mL) at −78 °C, the solution of ^*n*^BuLi in hexane (5.9 mmol, 3.8 mL, 1.55 M) was added dropwise. After stirring at −78 °C for 30 min, bromoethane (5.9 mmol, 0.64 g, 0.44 mL, *d* = 1.46) was slowly added, then stirred at rt for 1 h. To the resulting mixture at −78 °C, ^*n*^BuLi in hexane (5.9 mmol, 3.8 mL, 1.55 M) was farther added dropwise and stirred for 30 min, then a solution of acetone (5.9 mmol, 0.34 g, 0.43 mL, *d* = 0.79) in THF (6 mL) was slowly added. After stirring for 1 h, the reaction mixture was allowed to warm to rt and stirred for 1 h, then quenched with sat. NH_4_Cl aq. at 0 °C. The aquous layer was extracted with EtOAc (5 mL × 3) and the combined organic layers were dried over anh. Na_2_SO_4_, filtered, and concentrated. The crude product was dissolved in a solution of THF (6 mL), H_2_O (6 mL) and AcOH (12 mmol, 0.72 g, 0.69 mL, *d* = 1.05) and stirred under reflux condition for 16 h. The aquous layer was extracted with EtOAc (5 mL × 2) and the combined organic layers were washed with sat. NaHCO_3_ aq., dried over anh. Na_2_SO_4_, filtered, and concentrated. The crude product was purified by silica gel chromatography (hexane/EtOAc = 5/1) to afford a mixture of (*E*)-**1i** and (*Z*)-**1i** (1.28 mmol, 0.27 g, 22%). The mixture was treated with catalytic amount of HCl aq. in THF (2 mL) under reflux condition for 16 h and purified by silica gel chromatography (hexane/EtOAc = 5/1) to afford (*E*)-**1i** (1.02 mmol, 0.21 mg, 79%): yellow oil; ^1^H NMR (400 MHz, CDCl_3_) δ 7.51 (d, *J* = 1.8 Hz, 1H), 6.87 (s, 1H), 6.79 (d, *J* = 3.5 Hz, 1H), 6.52 (dd, *J* = 3.5, 1.8 Hz, 1H), 4.25 (s, 1H), 2.94 (q, *J* = 7.5 Hz, 2H), 1.45 (s, 6H), 1.21 (t, *J* = 7.5 Hz, 3H); ^13^C­{^1^H} NMR (100 MHz, CDCl_3_) δ 203.1, 153.6, 150.6, 144.6, 113.2, 112.5, 111.7, 75.8, 26.8, 23.0, 14.4; HRMS (APCI, positive) *m*/*z*: [M + H]^+^ calcd. for C_12_H_17_O_3_^+^ 209.1172; found 209.1177.

**(*E*)-2-Hydroxy-2-methyl-5-(thiophen-2-yl)­hept-4-en-3-one ((*E*)-1j)**: To a solution of **S10** (10.4 mmol, 2.30 g) in THF (20 mL) at −78 °C, the solution of ^*n*^BuLi in hexane (10.4 mmol, 6.7 mL, 1.55 M) was added dropwise. After stirring at −78 °C for 30 min, bromoethane (10.4 mmol, 1.13 g, 0.78 mL, *d* = 1.46) was slowly added, then stirred at rt for 1 h. To the resulting mixture at −78 °C, ^*n*^BuLi in hexane (10.4 mmol, 6.7 mL, 1.55 M) was farther added dropwise and stirred for 30 min, then a solution of acetone (10.4 mmol, 0.60 g, 0.76 mL, *d* = 0.79) in THF (10 mL) was slowly added. After stirring for 1 h, the reaction mixture was allowed to warm to rt and stirred for 1 h, then quenched with sat. NH_4_Cl aq. at 0 °C. The aqueous layer was extracted with EtOAc (10 mL × 3) and the combined organic layers were dried over anh. Na_2_SO_4_, filtered, and concentrated. The crude product was dissolved in a solution of THF (10 mL), H_2_O (10 mL), and AcOH (20.8 mmol, 1.26 g, 1.2 mL, *d* = 1.05) and stirred under reflux condition for 16 h. The aquous layer was extracted with EtOAc (10 mL × 2) and the combined organic layers were washed with sat. NaHCO_3_ aq., dried over anh. Na_2_SO_4_, filtered, and concentrated. The crude product was purified by silica gel chromatography (hexane/EtOAc = 5/1) to afford (*E*)-**1j** (0.57 mmol, 0.13 g, 5%). The product was also obtained by the isomerization of (*Z*)-**1j** (1.4 mmol, 0.32 g), which was treated with catalytic amount of HCl aq. in THF (2 mL) under reflux condition for 16 h and purified by silica gel chromatography (hexane/EtOAc = 5/1) to afford (*E*)-**1j** (1.31 mmol, 0.29 g, 91%): yellow oil; ^1^H NMR (400 MHz, CDCl_3_) δ 7.43 (dd, *J* = 3.8, 1.3 Hz, 1H), 7.40 (dd, *J* = 5.0, 1.0 Hz, 1H), 7.10 (dd, *J* = 5.0, 3.8 Hz, 1H), 6.74 (s, 1H), 4.18 (s, 1H), 3.08 (q, *J* = 7.5 Hz, 2H), 1.44 (s, 6H), 1.23 (t, *J* = 7.5 Hz, 3H); ^13^C­{^1^H} NMR (100 MHz, CDCl_3_) δ 202.7, 156.7, 144.7, 128.4, 128.1, 127.8, 114.0, 75.8, 26.9, 25.5, 14.2; HRMS (APCI, positive) *m*/*z*: [M + H]^+^ calcd. for C_12_H_17_O_2_S^+^ 225.0943; found 225.0943.

**(*E*)-2-Hydroxy-2-methyl-5-phenylundec-4-en-3-one ((*E*)-1k)**: To a solution of **S1** (5 mmol, 1.08 g, 1.0 mL, *d* = 1.05) in THF (10 mL) at −78 °C, the solution of ^*n*^BuLi in hexane (5.0 mmol, 3.2 mL, 1.55 M) was added dropwise. After stirring at −78 °C for 30 min, 1-bromohexane (5.0 mmol, 0.83 g, 0.70 mL, *d* = 1.18) was slowly added, then stirred at rt for 1 h. To the resulting mixture at −78 °C, ^*n*^BuLi in hexane (5.0 mmol, 3.2 mL, 1.55 M) was further added dropwise and stirred for 30 min, then a solution of acetone (5.0 mmol, 0.30 g, 0.37 mL, *d* = 0.79) in THF (5 mL) was slowly added. After stirring for 1 h, the reaction mixture was allowed to warm to rt and stirred for 1 h, then quenched with sat. NH_4_Cl aq. at 0 °C. The aqueous layer was extracted with EtOAc (5 mL × 3) and the combined organic layers were dried over anh. Na_2_SO_4_, filtered, and concentrated. The crude product was dissolved in a solution of THF (5 mL), H_2_O (5 mL), and AcOH (10 mmol, 0.59 g, 0.57 mL, *d* = 1.05) and stirred under reflux condition for 16 h. The aqueous layer was extracted with EtOAc (5 mL × 2) and the combined organic layers were washed with sat. NaHCO_3_ aq., dried over anh. Na_2_SO_4_, filtered, and concentrated. The crude product was purified by silica gel chromatography (hexane/EtOAc = 10/1) to afford (*E*)-**1k** (0.45 mmol, 0.12 g, 9%). The product was also obtained by the isomerization of (*Z*)-**1k** (2.3 mmol, 0.62 g), which was treated with catalytic amount of HCl aq. in THF (2 mL) under reflux condition for 16 h and purified by silica gel chromatography (hexane/EtOAc = 10/1) to afford (*E*)-**1k** (1.82 mmol, 0.50 g, 81%): yellow oil; ^1^H NMR (400 MHz, CDCl_3_) δ 7.49–7.38 (m, 5H), 6.56 (s, 1H), 4.21 (s, 1H), 3.08 (t, *J* = 7.6 Hz, 2H), 1.47–1.20 (m, 14H), 0.86 (t, *J* = 7.0 Hz, 3H); ^13^C­{^1^H} NMR (100 MHz, CDCl_3_) δ 203.3, 163.9, 141.7, 129.4, 128.7, 126.8, 118.1, 75.8, 31.9, 31.5, 29.4, 29.0, 26.8, 22.5, 14.0; HRMS (APCI, positive) *m*/*z*: [M + H]^+^ calcd. for. C_18_H_27_O_2_^+^ 275.2006; found 275.2004.

**(*E*)-2-Hydroxy-2,7-dimethyl-5-phenyloct-4-en-3-one ((*E*)-1l)**: To a solution of **S1** (5 mmol, 1.08 g, 1.0 mL, *d* = 1.05) in THF (10 mL) at −78 °C, the solution of ^*n*^BuLi in hexane (5.0 mmol, 3.2 mL, 1.55 M) was added dropwise. After stirring at −78 °C for 30 min, 1-bromo-2-methylpropane (5.0 mmol, 0.69 g, 0.54 mL, *d* = 1.27) was slowly added, then stirred at rt for 1 h. To the resulting mixture at −78 °C, ^*n*^BuLi in hexane (5.0 mmol, 3.2 mL, 1.55 M) was farther added dropwise and stirred for 30 min, then a solution of acetone (5.0 mmol, 0.30 g, 0.37 mL, *d* = 0.79) in THF (5 mL) was slowly added. After stirring for 1 h, the reaction mixture was allowed to warm to rt and stirred for 1 h, then quenched with sat. NH_4_Cl aq. at 0 °C. The aqueous layer was extracted with EtOAc (5 mL × 3) and the combined organic layers were dried over anh. Na_2_SO_4_, filtered, and concentrated. The crude product was dissolved in a solution of THF (5 mL), H_2_O (5 mL), and AcOH (10 mmol, 0.60 g, 0.57 mL, *d* = 1.05) and stirred under reflux condition for 16 h. The aquous layer was extracted with EtOAc (5 mL × 2) and the combined organic layers were washed with sat. NaHCO_3_ aq., dried over anh. Na_2_SO_4_, filtered, and concentrated. The crude product was purified by silica gel chromatography (hexane/EtOAc = 5/1) to afford (*E*)-**1l** (0.21 mmol, 51 mg, 4%). The product was also obtained by the isomerization of (*Z*)-**1l** (1.8 mmol, 0.44 g), which was treated with catalytic amount of HCl aq. in THF (2 mL) under reflux condition for 16 h and purified by silica gel chromatography (hexane/EtOAc = 5/1) to afford (*E*)-**1l** (0.88 mmol, 0.22 g, 50%): orange oil; ^1^H NMR (400 MHz, CDCl_3_) δ 7.43–7.39 (m, 5H), 6.57 (s, 1H), 4.19 (s, 1H), 3.08 (d, *J* = 7.3 Hz, 2H), 1.66 (hept, *J* = 6.8 Hz, 1H), 1.44 (s, 6H), 0.86 (d, *J* = 6.8 Hz, 6H); ^13^C­{^1^H} NMR (100 MHz, CDCl_3_) δ 203.6, 162.9, 142.0, 129.2, 128.6, 126.8, 119.6, 75.9, 39.5, 28.1, 26.8, 22.3; HRMS (APCI, positive) *m*/*z*: [M + H]^+^ calcd. for. C_16_H_23_O_2_^+^ 247.1693; found 247.1697.

**(*E*)-2-Hydroxy-2-methyl-5,6-diphenylhex-4-en-3-one ((*E*)-1m)**: To a solution of **S1** (5 mmol, 1.08 g, 1.0 mL, *d* = 1.05) in THF (10 mL) at −78 °C, the solution of ^*n*^BuLi in hexane (5.0 mmol, 3.2 mL, 1.55 M) was added dropwise. After stirring at −78 °C for 30 min, benzyl bromide (5.0 mmol, 0.85 g, 0.59 mL, *d* = 1.44) was slowly added, then stirred at rt for 1 h. To the resulting mixture at −78 °C, ^*n*^BuLi in hexane (5.0 mmol, 3.2 mL, 1.55 M) was farther added dropwise and stirred for 30 min, then a solution of acetone (5.0 mmol, 0.30 g, 0.37 mL, *d* = 0.79) in THF (5 mL) was slowly added. After stirring for 1 h, the reaction mixture was allowed to warm to rt and stirred for 1 h, then quenched with sat. NH_4_Cl aq. at 0 °C. The aqueous layer was extracted with EtOAc (5 mL × 3) and the combined organic layers were dried over anh. Na_2_SO_4_, filtered, and concentrated. The crude product was dissolved in a solution of THF (5 mL), H_2_O (5 mL,) and AcOH (10 mmol, 0.60 g, 0.57 mL, *d* = 1.05) and stirred under reflux condition for 16 h. The aquous layer was extracted with EtOAc (5 mL × 2) and the combined organic layers were washed with sat. NaHCO_3_ aq., dried over anh. Na_2_SO_4_, filtered, and concentrated. The crude product was purified by silica gel chromatography (hexane/EtOAc = 5/1) to afford (*E*)-**1m** (0.39 mmol, 0.11 g, 8%). The product was also obtained by the isomerization of (*Z*)-**1m** (2.4 mmol, 0.67 g), which was treated with catalytic amount of HCl aq. in THF (2 mL) under reflux condition for 16 h and purified by silica gel chromatography (hexane/EtOAc = 5/1) to afford (*E*)-**1m** (1.24 mmol, 0.35 g, 52%): yellow oil; ^1^H NMR (400 MHz, CDCl_3_) δ 7.45–7.42 (m, 2H), 7.35–7.33 (m, 3H), 7.20–7.18 (m, 2H), 7.15–7.13 (m, 3H), 6.77 (s, 1H), 4.48 (s, 2H), 4.12 (s, 1H), 1.47 (s, 6H); ^13^C­{^1^H} NMR (100 MHz, CDCl_3_) δ 203.4, 159.7, 141.2, 138.3, 129.5, 128.6, 128.5, 128.4, 127.1, 126.2, 119.5, 76.0, 37.0, 26.7; HRMS (APCI, positive) *m*/*z*: [M + H]^+^ calcd. for. C_19_H_21_O_2_^+^ 281.1536; found 281.1534.

**(*E*)-1-(1-Hydroxycyclopentyl)-3-phenylpent-2-en-1-one ((*E*)-1n)**: To a solution of **S1** (5 mmol, 1.08 g, 1.0 mL, *d* = 1.05) in THF (10 mL) at −78 °C, the solution of ^*n*^BuLi in hexane (5.0 mmol, 3.2 mL, 1.55 M) was added dropwise. After stirring at −78 °C for 30 min, bromoethane (5.0 mmol, 0.54 g, 0.37 mL, *d* = 1.46) was slowly added, then stirred at rt for 1 h. To the resulting mixture at −-78 °C, ^*n*^BuLi in hexane (5.0 mmol, 3.2 mL, 1.55 M) was farther added dropwise and stirred for 30 min, then a solution of cyclopentanone (5.0 mmol, 0.42 g, 0.44 mL, *d* = 0.95) in THF (5 mL) was slowly added. After stirring for 1 h, the reaction mixture was allowed to warm to rt and stirred for 1 h, then quenched with sat. NH_4_Cl aq. at 0 °C. The aqueous layer was extracted with EtOAc (5 mL × 3) and the combined organic layers were dried over anh. Na_2_SO_4_, filtered, and concentrated. The crude product was dissolved in a solution of THF (5 mL), H_2_O (5 mL), and AcOH (10 mmol, 0.60 g, 0.57 mL, *d* = 1.05) and stirred under reflux condition for 16 h. The aquous layer was extracted with EtOAc (5 mL × 2) and the combined organic layers were washed with sat. NaHCO_3_ aq., dried over anh. Na_2_SO_4_, filtered, and concentrated. The crude product was purified by silica gel chromatography (hexane/EtOAc = 10/1) to afford (*E*)-**1n** (0.70 mmol, 0.17 g, 14%). The product was also obtained by the isomerization of (*Z*)-**1n** (3.0 mmol, 0.74 g), which was treated with catalytic amount of HCl aq. in THF (3 mL) under reflux condition for 16 h and purified by silica gel chromatography (hexane/EtOAc = 10/1) to afford (*E*)-**1n** (2.19 mmol, 0.53 g, 73%): yellow oil; ^1^H NMR (400 MHz, CDCl_3_) δ 7.46–7.43 (m, 2H), 7.41–7.39 (m, 3H), 6.47 (s, 1H), 4.30 (s, 1H), 3.11 (q, *J* = 7.5 Hz, 2H), 2.10–1.97 (m, 4H), 1.82–1.76 (m, 4H), 1.10 (t, *J* = 7.5 Hz, 3H); ^13^C­{^1^H} NMR (100 MHz, CDCl_3_) δ 203.1, 164.3, 141.4, 129.3, 128.7, 126.8, 118.0, 86.7, 39.7, 25.9, 25.2, 13.4; HRMS (APCI, positive) *m*/*z*: [M + H]^+^ calcd. for. C_16_H_21_O_2_^+^ 245.1536; found 245.1543.

**(*E*)-1-(1-Hydroxycyclohexyl)-3-phenylpent-2-en-1-one ((*E*)-1o)**: To a solution of **S1** (5 mmol, 1.08 g, 1.0 mL, *d* = 1.05) in THF (10 mL) at −78 °C, the solution of ^*n*^BuLi in hexane (5.0 mmol, 3.2 mL, 1.55 M) was added dropwise. After stirring at −78 °C for 30 min, bromoethane (5.0 mmol, 0.54 g, 0.37 mL, *d* = 1.46) was slowly added, then stirred at rt for 1 h. To the resulting mixture at −78 °C, ^*n*^BuLi in hexane (5.0 mmol, 3.2 mL, 1.55 M) was farther added dropwise and stirred for 30 min, then a solution of cyclohexanone (5.0 mmol, 0.49 g, 0.52 mL, *d* = 0.95) in THF (5 mL) was slowly added. After stirring for 1 h, the reaction mixture was allowed to warm to rt and stirred for 1 h, then quenched with sat. NH_4_Cl aq. at 0 °C. The aquous layer was extracted with EtOAc (5 mL × 3) and the combined organic layers were dried over anh. Na_2_SO_4_, filtered, and concentrated. The crude product was dissolved in a solution of THF (5 mL), H_2_O (5 mL), and AcOH (10 mmol, 0.60 g, 0.57 mL, *d* = 1.05) and stirred under reflux condition for 16 h. The aquous layer was extracted with EtOAc (5 mL × 2) and the combined organic layers were washed with sat. NaHCO_3_ aq., dried over anh. Na_2_SO_4_, filtered, and concentrated. The crude product was purified by silica gel chromatography (hexane/EtOAc = 20/1) to afford (*E*)-**1o** (0.65 mmol, 0.17 g, 13%). The product was also obtained by the isomerization of (*Z*)-**1o** (3.2 mmol, 0.82 g), which was treated with catalytic amount of HCl aq. in THF (3 mL) under reflux condition for 16 h and purified by silica gel chromatography (hexane/EtOAc = 20/1) to afford (*E*)-**1o** (2.30 mmol, 0.59 g, 72%): yellow oil; ^1^H NMR (400 MHz, CDCl_3_) δ 7.51–7.44 (m, 2H), 7.44–7.38 (m, 3H), 6.63 (s, 1H), 4.02 (s, 1H), 3.06 (q, *J* = 7.5 Hz, 2H), 1.82–1.58 (m, 7H), 1.56–1.48 (m, 2H), 1.37–1.20 (m, 1H), 1.09 (t, *J* = 7.5 Hz, 3H); ^13^C­{^1^H} NMR (100 MHz, CDCl_3_) δ 203.6, 164.4, 141.5, 129.3, 128.6, 126.8, 118.1, 77.7, 34.2, 25.4, 25.2, 21.3, 13.5; HRMS (APCI, positive) *m*/*z*: [M + H]^+^ calcd. for C_17_H_23_O_2_^+^ 259.1693; found 259.1693.

**(*E*)-1-Hydroxy-1,1,4-triphenylhex-3-en-2-one ((*E*)-1p)**: To a solution of **S1** (5 mmol, 1.08 g, 1.0 mL, *d* = 1.05) in THF (10 mL) at −78 °C, the solution of ^*n*^BuLi in hexane (5.0 mmol, 3.2 mL, 1.55 M) was added dropwise. After stirring at −78 °C for 30 min, bromoethane (5.0 mmol, 0.30 g, 0.37 mL, *d* = 1.46) was slowly added, then stirred at rt for 1 h. To the resulting mixture at −78 °C, ^*n*^BuLi in hexane (5.0 mmol, 3.2 mL, 1.55 M) was farther added dropwise and stirred for 30 min, then a solution of benzophenone (5.0 mmol, 0.91 g) in THF (5 mL) was slowly added. After stirring for 1 h, the reaction mixture was allowed to warm to rt and stirred for 1 h, then quenched with sat. NH_4_Cl aq. at 0 °C. The aqueous layer was extracted with EtOAc (5 mL × 3) and the combined organic layers were dried over anh. Na_2_SO_4_, filtered, and concentrated. The crude product was dissolved in a solution of THF (5 mL), H_2_O (5 mL), and AcOH (10 mmol, 0.60 g, 0.57 mL, *d* = 1.05) and stirred under reflux condition for 16 h. The aqueous layer was extracted with EtOAc (5 mL × 2) and the combined organic layers were washed with sat. NaHCO_3_ aq., dried over anh. Na_2_SO_4_, filtered, and concentrated. The crude product was purified by silica gel chromatography (hexane/EtOAc = 20/1) to afford (*E*)-**1p** (0.65 mmol, 0.17 g, 13%). The product was also obtained by the isomerization of (*Z*)-**1p** (2.6 mmol, 0.90 g), which was treated with catalytic amount of HCl aq. in THF (3 mL) under reflux condition for 16 h and purified by silica gel chromatography (hexane/EtOAc = 20/1) to afford (*E*)-**1p** (2.22 mmol, 0.76 g, 85%): white solid; mp 71.6–72.2 °C; ^1^H NMR (400 MHz, CDCl_3_) δ 7.46–7.40 (m, 4H), 7.40–7.22 (m, 11H), 6.59 (s, 1H), 5.38 (s, 1H), 3.09 (q, *J* = 7.5 Hz, 2H), 1.06 (t, *J* = 7.5 Hz, 3H); ^13^C­{^1^H} NMR (100 MHz, CDCl_3_) δ 198.6, 164.1, 142.1, 140.9, 129.5, 128.6, 128.3, 128.3, 128.0, 126.8, 120.3, 84.9, 25.3, 13.3; HRMS (APCI, positive) *m*/*z*: [M–OH]^+^ calcd. for C_24_H_21_O_1_^+^ 325.1587; found 325.1581.

**(*E*)-2-Hydroxy-2-methyl-5-phenylhex-4-en-3-one ((*E*)-3)**: To a solution of **S1** (5 mmol, 1.08 g, 1.0 mL, *d* = 1.05) in THF (10 mL) at −78 °C, the solution of ^*n*^BuLi in hexane (5.0 mmol, 3.2 mL, 1.55 M) was added dropwise. After stirring at −78 °C for 30 min, iodomethane (5.0 mmol, 0.71 g, 0.31 mL, *d* = 2.28) was slowly added, then stirred at rt for 1 h. To the resulting mixture at −78 °C, ^*n*^BuLi in hexane (5.0 mmol, 3.2 mL, 1.55 M) was farther added dropwise and stirred for 30 min, then a solution of acetone (5.0 mmol, 0.30 g, 0.37 mL, *d* = 0.79) in THF (5 mL) was slowly added. After stirring for 1 h, the reaction mixture was allowed to warm to rt and stirred for 1 h, then quenched with sat. NH_4_Cl aq. at 0 °C. The aquous layer was extracted with EtOAc (5 mL × 3) and the combined organic layers were dried over anh. Na_2_SO_4_, filtered, and concentrated. The crude product was dissolved in a solution of THF (5 mL), H_2_O (5 mL), and AcOH (10 mmol, 0.60 g, 0.57 mL, *d* = 1.05) and stirred under reflux condition for 16 h. The aqueous layer was extracted with EtOAc (5 mL × 2) and the combined organic layers were washed with sat. NaHCO_3_ aq., dried over anh. Na_2_SO_4_, filtered, and concentrated. The crude product was purified by silica gel chromatography (hexane/EtOAc = 5/1) to afford (*E*)-**3** (0.45 mmol, 0.09 g, 9%). The product was also obtained by the isomerization of (*Z*)-**3** (1.5 mmol, 0.31 g), which was treated with catalytic amount of HCl aq. in THF (2 mL) under reflux condition for 16 h and purified by silica gel chromatography (hexane/EtOAc = 5/1) to afford (*E*)-**3** (0.98 mmol, 0.20 g, 65%): orange oil; ^1^H NMR (400 MHz, CDCl_3_) δ 7.53–7.46 (m, 2H), 7.44–7.38 (m, 3H), 6.70–6.64 (m, 1H), 4.19 (s, 1H), 2.62 (d, *J* = 1.3 Hz, 3H), 1.45 (s, 6H); ^13^C­{^1^H} NMR (100 MHz, CDCl_3_) δ 203.7, 158.6, 142.4, 129.5, 128.6, 126.5, 118.0, 75.8, 26.8, 19.1; HRMS (APCI, positive) *m*/*z*: [M + H]^+^ calcd. for C_13_H_17_O_2_^+^ 205.1223; found 205.1220.

**(*E*)-2,2-Dimethyl-5-phenylhept-4-en-3-one ((*E*)-4)**: To a solution of pinacolone (5.0 mmol, 0.50 g, 0.62 mL, *d* = 0.81) in CH_2_Cl_2_ (10 mL) at −78 °C, TiCl_4_ (5.5 mmol, 1.04 g, 0.60 mL, *d* = 1.73) and Bu_3_N (6.0 mmol, 1.09 g, 1.4 mL, *d* = 0.78) were added. After 30 min, propiophenone (5.0 mmol, 0.67 g, 0.66 mL, *d* = 1.01) was added, and stirred at this temperature. After 2 h, pyridine (25 mmol, 1.96 g, 2.0 mL, *d* = 0.98) was added, then allowed to warm to rt. After 16 h, the reaction mixture was diluted with EtOAc (10 mL) and hexane (10 mL), filtered through a pad of Celite and concentrated. The crude product was purified by silica gel chromatography (hexane/EtOAc = 40/1) to afford (*E*)-**4** (2.58 mmol, 0.56 g, 52%): pale yellow oil; ^1^H NMR (400 MHz, CDCl_3_) δ 7.47–7.32 (m, 5H), 6.61 (s, 1H), 2.98 (q, *J* = 7.5 Hz, 2H), 1.20 (s, 9H), 1.06 (t, *J* = 7.5 Hz, 3H); ^13^C­{^1^H} NMR (100 MHz, CDCl_3_) δ 206.3, 160.2, 142.0, 128.6, 128.5, 126.7, 120.6, 44.1, 26.6, 24.7, 13.5; HRMS (APCI, positive) *m*/*z*: [M + H]^+^ calcd. for C_15_H_21_O^+^ 217.1587; found 217.1576.

**(*E*)-2-Methyl-5-phenyl-2-((trimethylsilyl)­oxy)­hept-4-en-3-one (6)**: To a solution of DMAP (0.3 mmol, 36.7 mg), Et_3_N (1.5 mmol, 0.15 g, 0.21 mL, *d* = 0.73), and TMSCl (1.5 mmol, 0.16 g, 0.19 mL, *d* = 0.86) in CH_2_Cl_2_ (1.5 mL) **S12** (1.5 mmol, 0.30 g) was added. The reaction mixture was stirred at rt for 1 h. The mixture was filtered through a pad of Celite and washed with CH_2_Cl_2_. The eluent was washed with 1 M HCl aq., H_2_O, and brine. The organic layer was dried over anh. Na_2_SO_4_, filtered, and concentrated. The crude product was used next step without further purification. To a solution of the crude product in CH_2_Cl_2_ (12 mL) at 0 °C was added *m*-CPBA (1.3 mmol, 0.33 g, ca. 30% water). The reaction mixture was stirred at this temperature for 4 h. The reaction mixture was quenched with sat. NaHCO_3_ aq. The aqueous layer was extracted with CH_2_Cl_2_ (10 mL × 2). The combined organic layers were dried over anh. Na_2_SO_4_, filtered, and concentrated. The crude product was purified by silica gel chromatography (hexane/EtOAc = 20/1) to afford (*E*)-**6** (0.67 mmol, 0.19 g, 56%) as a pale yellow oil: ^1^H NMR (400 MHz, CDCl_3_) δ 7.50–7.47 (m, 2H), 7.41–7.36 (m, 3H), 6.98 (s, 1H), 3.05 (q, *J* = 7.4 Hz, 2H), 1.41 (s, 6H), 1.08 (t, *J* = 7.4 Hz, 3H), 0.15 (s, 9H); ^13^C­{^1^H} NMR (100 MHz, CDCl_3_) δ 204.2, 161.6, 141.9, 128.8, 128.5, 126.7, 119.5, 80.0, 27.4, 24.5, 13.5, 2.2; HRMS (APCI, positive) *m*/*z*: [M + H]^+^ calcd. for C_17_H_26_O_2_Si^+^ 291.1775; found 291.1776.

**2-Hydroxy-2,5-dimethyl-5-phenylhepta-2-one (2a)**: CuI (1.9 mg, 0.01 mmol) and **BmP-F** (10.1 mg, 0.02 mmol) were dissolved in THF (0.2 mL) and the mixture was stirred for 30 min, then cooled with ice bath. The solution of Me_3_Al in hexane (0.57 mL, 0.80 mmol, 1.4 M) was added dropwise. To the resulting solution was added (*E*)-**1a** (0.20 mmol, 43.7 mg, 42 μL, *d* = 1.03) at once. The reaction was carried out at 40 °C and monitored by TLC. After 24 h, a minimal amount of sat. NH_4_Cl aq. was added at 0 °C, then stirred for 30 min. The mixture was filtered through silica gel pad, washed with Et_2_O and concentrated. The crude product was purified by silica gel chromatography (hexane/EtOAc = 5/1) to afford **2a** (37.4 mg, 81%).

[*1 mmol scale*] CuI (9.5 mg, 0.05 mmol) and **BmP-F** (50.6 mg, 0.10 mmol) were dissolved in THF (1 mL) and the mixture was stirred for 30 min, then cooled with ice bath. The solution of Me_3_Al in hexane (2.9 mL, 4.0 mmol, 1.4 M) was added dropwise. To the resulting solution was added (*E*)-**1a** (1.00 mmol, 0.22 g, 0.21 mL, *d* = 1.03) at once. The reaction was carried out at 40 °C and monitored by TLC. After 24 h, a minimal amount of sat. NH_4_Cl aq. was added at 0 °C, then stirred for 30 min. The mixture was filtered through silica gel pad, washed with Et_2_O and concentrated. The crude product was purified by silica gel chromatography (hexane/EtOAc = 5/1) to afford **2a** (0.22 g, 90%, 95% ee): pale yellow oil; ^1^H NMR (400 MHz, CDCl_3_) δ 7.33–7.26 (m, 4H), 7.21–7.14 (m, 1H), 3.62 (s, 1H), 3.04 (d, *J* = 17.5 Hz, 1H), 2.78 (d, *J* = 17.5 Hz, 1H), 1.92–1.73 (m. 2H), 1.48 (s, 3H), 1.27 (s, 3H), 1.24 (s, 3H), 0.69 (t, *J* = 7.5 Hz, 3H); ^13^C­{^1^H} NMR (100 MHz, CDCl_3_) δ 212.6, 146.4, 128.1, 125.9, 125.8, 76.2, 47.0, 40.3, 35.4, 26.3, 23.4, 8.4 (One carbon atom was not found probably due to overlapping); [α]^26.0^_D_ (96% ee) = +1.53 (*c* 1.43, CHCl_3_); HRMS (APCI, positive) *m*/*z*: [M–OH]^+^ calcd. for. C_15_H_21_O^+^ 217.1587; found 217.1587; HPLC (CHIRALPAK IB, Daicel, 4.6 × 150 mm, hexane/^*i*^PrOH = 99/1, 1.0 mL/min, 210 nm), t_r_ (major) = 4.6 min, t_r_ (minor) = 4.9 min.

**2-Hydroxy-2,5-dimethyl-5-(p-tolyl)­heptan-3-one (2b)**: CuI (1.9 mg, 0.01 mmol) and **BmP-F** (10.1 mg, 0.02 mmol) were dissolved in THF (0.2 mL) and the mixture was stirred for 30 min, then cooled with ice bath. The solution of Me_3_Al in hexane (0.57 mL, 0.80 mmol, 1.4 M) was added dropwise. To the resulting solution was added (*E*)-**1b** (0.20 mol, 46.5 mg) at once. The reaction was carried out at 40 °C and monitored by TLC. After 24 h, a minimal amount of sat. NH_4_Cl aq. was added at 0 °C, then stirred for 30 min. The mixture was filtered through silica gel pad, washed with Et_2_O and concentrated. The crude product was purified by silica gel chromatography (hexane/EtOAc = 5/1) to afford **2b** (41.2 mg, 83%): pale yellow oil; ^1^H NMR (400 MHz, CDCl_3_) δ 7.16 (dt, *J* = 8.4, 2.1 Hz, 2H), 7.11 (d, *J* = 8.0 Hz, 2H), 3.64 (s, 1H), 3.01 (d, *J* = 17.4 Hz, 1H), 2.76 (d, *J* = 17.4 Hz, 1H), 2.31 (s, 3H), 1.88–1.72 (m, 2H), 1.45 (s, 3H), 1.27 (s, 3H), 1.24 (s, 3H), 0.69 (t, *J* = 7.4 Hz, 3H); ^13^C­{^1^H} NMR (100 MHz, CDCl_3_) δ 212.7, 143.4, 135.2, 128.8, 125.8, 76.2, 47.0, 40.0, 35.3, 26.3, 23.5, 20.8, 8.4 (One carbon atom was not found probably due to overlapping); [α]^29.0^_D_ (96% ee) = – 0.41 (*c* 1.08, CHCl_3_); HRMS (APCI, positive) *m*/*z*: [M–OH]^+^ calcd. for C_16_H_23_O^+^ 231.1744; found 231.1746; HPLC (CHIRALPAK IB, Daicel, 4.6 × 150 mm, hexane/^*i*^PrOH = 99/1, 1.0 mL/min, 210 nm), t_r_ (major) = 4.6 min, t_r_ (minor) = 5.0 min.

**2-Hydroxy-5-(4-methoxyphenyl)-2,5-dimethylheptan-3-one (2c)**: CuI (1.9 mg, 0.01 mmol) and **BmP-F** (10.1 mg, 0.02 mmol) were dissolved in THF (0.2 mL) and the mixture was stirred for 30 min, then cooled with ice bath. The solution of Me_3_Al in hexane (0.57 mL, 0.80 mmol, 1.4 M) was added dropwise. To the resulting solution was added (*E*)-**1c** (0.20 mmol, 49.7 mg, 46 μL, *d* = 1.07) at once. The reaction was carried out at 40 °C and monitored by TLC. After 24 h, a minimal amount of sat. NH_4_Cl aq. was added at 0 °C, then stirred for 30 min. The mixture was filtered through silica gel pad, washed with Et_2_O and concentrated. The crude product was purified by silica gel chromatography (hexane/EtOAc = 5/1) to afford **2c** (47.9 mg, 91%): pale yellow oil; ^1^H NMR (400 MHz, CDCl_3_) δ 7.19 (dt, *J* = 9.0, 2.8 Hz, 2H), 6.84 (dt, *J* = 9.0, 2.8 Hz, 2H), 3.78 (s, 3H), 3.66 (s, 1H), 3.00 (d, *J* = 17.5 Hz, 1H), 2.74 (d, *J* = 17.5 Hz, 1H), 1.89–1.70 (m, 2H), 1.45 (s, 3H), 1.26 (s, 3H), 1.22 (s, 3H), 0.68 (t, *J* = 7.2 Hz, 3H); ^13^C­{^1^H} NMR (100 MHz, CDCl_3_) δ 212.8, 157.4, 138.4, 126.9, 113.3, 76.2, 55.1, 47.0, 39.7, 35.4, 26.3, 26.2, 23.5, 8.4; [α]^29.0^_D_ (96% ee) = – 0.97 (*c* 1.29, CHCl_3_); HRMS (APCI, positive) *m*/*z*: [M–OH]^+^ calcd. for. C_16_H_23_O_2_^+^ 247.1693; found 247.1704; HPLC (CHIRALPAK IB, Daicel, 4.6 × 150 mm, hexane/^*i*^PrOH = 99/1, 1.0 mL/min, 210 nm), t_r_ (major) = 7.9 min, t_r_ (minor) = 9.8 min.

**5-(4-Fluorophenyl)-2-hydroxy-2,5-dimethylheptan-3-one (2d)**: CuI (1.9 mg, 0.01 mmol) and **BmP-F** (10.1 mg, 0.02 mmol) were dissolved in THF (0.2 mL) and the mixture was stirred for 30 min, then cooled with ice bath. The solution of Me_3_Al in hexane (0.57 mL, 0.80 mmol, 1.4 M) was added dropwise. To the resulting solution was added (*E*)-**1d** (0.20 mmol, 47.3 mg, 43 μL, *d* = 1.09) at once. The reaction was carried out at 40 °C and monitored by TLC. After 24 h, a minimal amount of sat. NH_4_Cl aq. was added at 0 °C, then stirred for 30 min. The mixture was filtered through silica gel pad, washed with Et_2_O and concentrated. The crude product was purified by silica gel chromatography (hexane/EtOAc = 5/1) to afford **2d** (45.6 mg, 91%): pale yellow oil; ^1^H NMR (400 MHz, CDCl_3_) δ 7.26–7.22 (m, 2H), 7.00–6.95 (m, 2H), 3.60 (s, 1H), 3.02 (d, *J* = 17.5 Hz, 1H), 2.77 (d, *J* = 17.5 Hz, 1H), 1.89–1.68 (m, 2H), 1.46 (s, 3H), 1.28 (s, 3H), 1.24 (s, 3H), 0.68 (t, *J* = 7.2 Hz, 3H); ^13^C­{^1^H} NMR (100 MHz, CDCl_3_) δ 212.5, 160.9 (d, *J* = 244.1 Hz), 142.1 (d, *J* = 2.9 Hz), 127.5 (d, *J* = 8.0 Hz), 114.7 (d, *J* = 21.1 Hz), 76.2, 47.0, 39.9, 35.7, 26.31, 26.28, 23.5, 8.3; ^19^F NMR (375 MHz, CDCl_3_) δ – 117.7; [α]^29.0^_D_ (94% ee) = +5.53 (*c* 1.35, CHCl_3_); HRMS (APCI, positive) *m*/*z*: [M–OH]^+^ calcd. for C_15_H_20_FO^+^ 235.1493; found 235.1499; HPLC (CHIRALCEL OD-H, Daicel, 4.6 × 250 mm, hexane/^*i*^PrOH = 99/1, 1.0 mL/min, 210 nm), t_r_ (major) = 23.8 min, t_r_ (minor) = 25.8 min.

**5-([1,1’-Biphenyl]-4-yl)-2-hydroxy-2,5-dimethylheptan-3-one (2e)**: CuI (1.9 mg, 0.01 mmol) and **BmP-F** (10.1 mg, 0.02 mmol) were dissolved in THF (0.2 mL) and the mixture was stirred for 30 min, then cooled with ice bath. The solution of Me_3_Al in hexane (0.57 mL, 0.80 mmol, 1.4 M) was added dropwise. To the resulting solution was added (*E*)-**1e** (0.20 mmol, 58.9 mg) at once. The reaction was carried out at 40 °C and monitored by TLC. After 24 h, a minimal amount of sat. NH_4_Cl aq. was added at 0 °C, then stirred for 30 min. The mixture was filtered through silica gel pad, washed with Et_2_O and concentrated. The crude product was purified by silica gel chromatography (hexane/EtOAc = 5/1) to afford **2e** (50.5 mg, 82%): pale yellow oil; ^1^H NMR (400 MHz, CDCl_3_) δ 7.60–7.5 (m, 2H), 7.56–7.53 (m, 2H), 7.41 (t, *J* = 7.6 Hz, 2H), 7.35–7.23 (m, 3H), 3.65 (s, 1H), 3.07 (d, *J* = 17.5 Hz, 1H), 2.81 (d, *J* = 17.5 Hz, 1H), 1.90–1.77 (m, 2H), 1.51 (s, 3H), 1.29 (s, 3H), 1.26 (s, 3H), 0.72 (t, *J* = 7.4 Hz, 3H); ^13^C­{^1^H} NMR (100 MHz, CDCl_3_) δ 212.6, 145.6, 140.7, 138.5, 128.6, 127.0, 126.9, 126.7, 126.4, 76.2, 46.8, 40.1, 35.4, 26.3, 23.5, 8.5 (One carbon atom was not found probably due to overlapping); [α]^30.0^_D_ (97% ee) = +1.84 (*c* 1.14, CHCl_3_); HRMS (APCI, positive) *m*/*z*: [M–OH]^+^ calcd. for C_21_H_25_O^+^ 293.1900; found 293.1900; HPLC (CHIRALPAK IA, Daicel, 4.6 × 250 mm, hexane/^*i*^PrOH = 99/1, 1.0 mL/min, 210 nm), t_r_ (major) = 25.5 min, t_r_ (minor) = 29.0 min.

**2-Hydroxy-5-(2-methoxyphenyl)-2,5-dimethylheptan-3-one (2f)**: CuI (1.9 mg, 0.01 mmol) and **BmP-F** (10.1 mg, 0.02 mmol) were dissolved in THF (0.2 mL) and the mixture was stirred for 30 min, then cooled with ice bath. The solution of Me_3_Al in hexane (0.57 mL, 0.80 mmol, 1.4 M) was added dropwise. To the resulting solution was added (*E*)-**1f** (0.20 mmol, 49.7 mg, 47 μL, *d* = 1.06) at once. The reaction was carried out at 40 °C and monitored by TLC. After 24 h, a minimal amount of sat. NH_4_Cl aq. was added at 0 °C, then stirred for 30 min. The mixture was filtered through silica gel pad, washed with Et_2_O, and concentrated. The crude product was purified by silica gel chromatography (hexane/EtOAc = 5/1) to afford **2f** (43.0 mg, 81%): colorless oil; ^1^H NMR (400 MHz, CDCl_3_) δ 7.28 (dd, *J* = 7.8, 1.8 Hz, 1H), 7.23–7.17 (m, 1H), 6.94 (td, *J* = 7.5, 1.3 Hz, 1H), 6.82 (dd, *J* = 8.1, 1.1 Hz, 1H), 3.84 (broad singlet, 1H), 3.78 (s, 3H), 3.59 (d, *J* = 18.3 Hz, 1H), 2.83 (d, *J* = 18.0 Hz, 1H), 2.21–2.11 (m, 1H), 1.66–1.58 (m, 1H), 1.48 (s, 3H), 1.32 (s, 3H), 1.25 (s, 3H), 0.62 (t, *J* = 7.6 Hz, 3H); ^13^C­{^1^H} NMR (100 MHz, CDCl_3_) δ 213.3, 157.4, 133.3, 128.9, 127.3, 120.4, 110.9, 76.0, 54.8, 45.0, 40.4, 32.5, 26.4, 26.4, 24.4, 8.7; [α]^27.0^_D_ (92% ee) = +13.3 (*c* 1.19, CHCl_3_); HRMS (APCI, positive) *m*/*z*: [M + H]^+^ calcd. for C_16_H_25_O_3_^+^ 265.1798; found 265.1796; HPLC (CHIRALPAK IB, Daicel, 4.6 × 150 mm, hexane/^*i*^PrOH = 99/1, 1.0 mL/min, 210 nm), t_r_ (major) = 4.43 min, t_r_ (minor) = 4.83 min.

**2-Hydroxy-2,5-dimethyl-5-(naphthalen-2-yl)­heptan-3-one (2g)**: CuI (1.9 mg, 0.01 mmol) and **BmP-F** (10.1 mg, 0.02 mmol) were dissolved in THF (0.2 mL) and the mixture was stirred for 30 min, then cooled with ice bath. The solution of Me_3_Al in hexane (0.57 mL, 0.80 mmol, 1.4 M) was added dropwise. To the resulting solution was added (*E*)-**1g** (0.20 mmol, 53.7 mg) at once. The reaction was carried out at 40 °C and monitored by TLC. After 24 h, a minimal amount of sat. NH_4_Cl aq. was added at 0 °C, then stirred for 30 min. The mixture was filtered through silica gel pad, washed with Et_2_O, and concentrated. The crude product was purified by silica gel chromatography (hexane/EtOAc = 5/1) to afford **2g** (50.9 mg, 90%): pale yellow oil; ^1^H NMR (400 MHz, CDCl_3_) δ 7.81–7.77 (m, 3H), 7.70 (d, *J* = 2.0 Hz, 1H), 7.48–7.41 (m, 3H), 3.60 (s, 1H), 3.15 (d, *J* = 17.8 Hz, 1H), 2.87 (d, *J* = 17.8 Hz, 1H), 1.99–1.83 (m, 2H), 1.59 (s, 3H), 1.29 (s, 3H), 1.25 (s, 3H), 0.69 (t, *J* = 7.2 Hz, 3H); ^13^C­{^1^H} NMR (100 MHz, CDCl_3_) δ 212.5, 143.9, 133.3, 131.7, 127.9, 127.7, 127.3, 125.9, 125.4, 124.8, 124.4, 76.2, 46.9, 40.5, 35.3, 26.4, 26.4, 23.4, 8.5; [α]^30.0^_D_ (94% ee) = +7.11 (*c* 1.00, CHCl_3_); HRMS (APCI, positive) *m*/*z*: [M–OH]^+^ calcd. for C_19_H_23_O^+^ 267.1744; found 267.1750; HPLC (CHIRALPAK IB, Daicel, 4.6 × 150 mm, hexane/^*i*^PrOH = 99/1, 1.0 mL/min, 210 nm), t_r_ (major) = 5.5 min, t_r_ (minor) = 6.2 min.

**2-Hydroxy-2,5-dimethyl-5-(naphthalen-1-yl)­heptan-3-one (2h)**: CuI (1.9 mg, 0.01 mmol) and **BmP-F** (10.1 mg, 0.02 mmol) were dissolved in THF (0.2 mL) and the mixture was stirred for 30 min, then cooled with ice bath. The solution of Me_3_Al in hexane (0.57 mL, 0.80 mmol, 1.4 M) was added dropwise. To the resulting solution was added (*E*)-**1h** (0.20 mmol, 53.7 mg) at once. The reaction was carried out at 40 °C and monitored by TLC. After 24 h, a minimal amount of sat. NH_4_Cl aq. was added at 0 °C, then stirred for 30 min. The mixture was filtered through silica gel pad, washed with Et_2_O and concentrated. The crude product was purified by silica gel chromatography (hexane/EtOAc = 5/1) to afford **2h** (26.1 mg, 46%): colorless oil; ^1^H NMR (400 MHz, CDCl_3_) δ 8.32–8.29 (m, 1H), 7.88–7.86 (m, 1H), 7.73 (d, *J* = 8.0 Hz, 1H), 7.51–7.40 (m, 4H), 3.63 (s, 1H), 3.60–3.58 (m, 1H), 3.15 (d, *J* = 18.0 Hz, 1H), 2.53–2.44 (m, 1H), 2.07–2.00 (m, 1H), 1.71 (s, 3H), 1.28 (s, 3H), 1.12 (s, 3H), 0.61 (t, *J* = 7.4 Hz, 3H); ^13^C­{^1^H} NMR (100 MHz, CDCl_3_) δ 212.6, 140.9, 134.9, 131.3, 130.1, 127.9, 126.4, 125.1, 125.1, 125.0, 124.6, 76.3, 46.4, 42.3, 33.9, 27.0, 26.5, 26.4, 8.8; [α]^27.0^_D_ (94% ee) = – 14.4 (*c* 1.04, CHCl_3_); HRMS (APCI, positive) *m*/*z*: [M–OH]^+^ calcd. for C_19_H_23_O^+^ 267.1744; found 267.1739; HPLC (CHIRALPAK IA, Daicel, 4.6 × 250 mm, hexane/^*i*^PrOH = 99/1, 1.0 mL/min, 210 nm), t_r_ (minor) = 8.77 min, t_r_ (major) = 9.30 min.

**5-(Furan-2-yl)-2-hydroxy-2,5-dimethylheptan-3-one (2i)**: CuI (1.9 mg, 0.01 mmol) and **BmP-F** (10.1 mg, 0.02 mmol) were dissolved in THF (0.2 mL) and the mixture was stirred for 30 min, then cooled with ice bath. The solution of Me_3_Al in hexane (0.57 mL, 0.80 mmol, 1.4 M) was added dropwise. To the resulting solution was added (*E*)-**1i** (0.20 mmol, 41.7 mg, 40 μL, *d* = 1.05) at once. The reaction was carried out at 40 °C and monitored by TLC. After 24 h, a minimal amount of sat. NH_4_Cl aq. was added at 0 °C, then stirred for 30 min. The mixture was filtered through silica gel pad, washed with Et_2_O and concentrated. The crude product was purified by silica gel chromatography (hexane/EtOAc = 5/1) to afford **2i** (39.6 mg, 87%): pale yellow oil; ^1^H NMR (400 MHz, CDCl_3_) δ 7.28 (dd, *J* = 1.9, 0.9 Hz, 1H), 6.28 (dd, *J* = 3.1, 1.9 Hz, 1H), 6.02 (dd, *J* = 3.3, 0.8 Hz, 1H), 3.72 (s, 1H), 2.98 (d, *J* = 17.0 Hz, 1H), 2.76 (d, *J* = 17.0 Hz, 1H), 1.86–1.69 (m, 2H), 1.39 (s, 3H), 1.30 (s, 3H), 1.27 (s, 3H), 0.74 (t, *J* = 7.6 Hz, 3H); ^13^C­{^1^H} NMR (100 MHz, CDCl_3_) δ 212.7, 160.0, 140.6, 110.0, 104.7, 76.5, 43.9, 38.5, 33.1, 26.2, 26.1, 22.5, 8.5; [α]^26.0^_D_ (95% ee) = – 12.7 (*c* 1.01, CHCl_3_); HRMS (APCI, positive) *m*/*z*: [M + H]^+^ calcd. for C_13_H_21_O_3_^+^ 225.1485; found 225.1487; HPLC (CHIRALPAK IA, Daicel, 4.6 × 250 mm, hexane/^*i*^PrOH = 99/1, 1.0 mL/min, 210 nm), t_r_ (minor) = 7.69 min, t_r_ (major) = 8.23 min.

**2-Hydroxy-2,5-dimethyl-5-(thiophen-2-yl)­heptan-3-one (2j)**: CuI (1.9 mg, 0.01 mmol) and **BmP-F** (10.1 mg, 0.02 mmol) were dissolved in THF (0.2 mL) and the mixture was stirred for 30 min, then cooled with ice bath. The solution of Me_3_Al in hexane (0.57 mL, 0.80 mmol, 1.4 M) was added dropwise. To the resulting solution was added (*E*)-**1j** (0.20 mmol, 44.9 mg, 41 μL, *d* = 1.09) at once. The reaction was carried out at 40 °C and monitored by TLC. After 24 h, a minimal amount of sat. NH_4_Cl aq. was added at 0 °C, then stirred for 30 min. The mixture was filtered through silica gel pad, washed with Et_2_O and concentrated. The crude product was purified by silica gel chromatography (hexane/EtOAc = 5/1) to afford **2j** (36.7 mg, 76%): pale yellow oil; ^1^H NMR (400 MHz, CDCl_3_) δ 7.13 (dd, *J* = 5.0, 1.3 Hz, 1H), 6.92 (dd, *J* = 5.0, 3.5 Hz, 1H), 6.81 (dd, *J* = 3.5, 1.3 Hz, 1H), 3.66 (s, 1H), 2.96 (d, *J* = 17.5 Hz, 1H), 2.84 (d, *J* = 17.3 Hz, 1H), 1.92–1.85 (m, 2H), 1.53 (s, 3H), 1.27 (s, 3H), 1.26 (s, 3H), 0.80 (t, *J* = 7.5 Hz, 3H); ^13^C­{^1^H} NMR (100 MHz, CDCl_3_) δ 212.4, 152.9, 126.5, 123.1, 122.8, 76.4, 47.3, 40.0, 36.0, 26.2, 26.2, 25.2, 8.6; [α]^25.0^_D_ (97% ee) = – 6.55 (*c* 1.01, CHCl_3_); HRMS (APCI, positive) *m*/*z*: [M + H]^+^ calcd. for C_13_H_21_O_2_S^+^ 241.1257; found 241.1257; HPLC (CHIRALPAK IC, Daicel, 4.6 × 250 mm, hexane/^*i*^PrOH = 99/1, 1.0 mL/min, 210 nm), t_r_ (major) = 10.2 min, t_r_ (minor) = 11.4 min.

**2-Hydroxy-2,5-dimethyl-5-phenylundecan-3-one (2k)**: CuI (1.9 mg, 0.01 mmol) and **BmP-F** (10.1 mg, 0.02 mmol) were dissolved in THF (0.2 mL) and the mixture was stirred for 30 min, then cooled with ice bath. The solution of Me_3_Al in hexane (0.57 mL, 0.80 mmol, 1.4 M) was added dropwise. To the resulting solution was added (*E*)-**1k** (0.20 mmol, 54.9 mg, 57 μL, *d* = 0.97) at once. The reaction was carried out at 40 °C and monitored by TLC. After 24 h, a minimal amount of sat. NH_4_Cl aq. was added at 0 °C, then stirred for 30 min. The mixture was filtered through silica gel pad, washed with Et_2_O and concentrated. The crude product was purified by silica gel chromatography (hexane/EtOAc = 10/1) to afford **2k** (54.0 mg, 92%): colorless oil; ^1^H NMR (400 MHz, CDCl_3_) δ 7.31–7.25 (m, 4H), 7.20–7.15 (m, 1H), 3.62 (s, 1H), 3.03 (d, *J* = 17.5 Hz, 1H), 2.78 (d, *J* = 17.3 Hz, 1H), 1.82–1.68 (m, 2H), 1.49 (s, 3H), 1.27–1.14 (m, 13H), 1.01–0.88 (m, 1H), 0.84 (t, *J* = 6.9 Hz, 3H); ^13^C­{^1^H} NMR (100 MHz, CDCl_3_) δ 212.6, 146.8, 128.1, 125.8, 125.7, 76.2, 47.3, 43.0, 40.1, 31.7, 29.8, 26.3, 24.0, 23.9, 22.6, 14.0 (One carbon atom was not found probably due to overlapping); [α]^26.0^_D_ (94% ee) = – 4.55 (*c* 1.02, CHCl_3_); HRMS (APCI, positive) *m*/*z*: [M–OH]^+^ calcd. for C_19_H_29_O^+^ 273.2213; found 273.2213; HPLC (CHIRALPAK IC, Daicel, 4.6 × 250 mm, hexane/^*i*^PrOH = 99/1, 1.0 mL/min, 210 nm), t_r_ (major) = 8.47 min, t_r_ (minor) = 10.2 min.

**2-Hydroxy-2,5,7-trimethyl-5-phenyloctan-3-one (2l)**: CuI (1.9 mg, 0.01 mmol) and **BmP-F** (10.1 mg, 0.02 mmol) were dissolved in THF (0.2 mL) and the mixture was stirred for 30 min, then cooled with ice bath. The solution of Me_3_Al in hexane (0.57 mL, 0.80 mmol, 1.4 M) was added dropwise. To the resulting solution was added (*E*)-**1l** (0.20 mmol, 49.3 mg, 50 μL, *d* = 0.98) at once. The reaction was carried out at 40 °C and monitored by TLC. After 24 h, a minimal amount of sat. NH_4_Cl aq. was added at 0 °C, then stirred for 30 min. The mixture was filtered through silica gel pad, washed with Et_2_O, and concentrated. The crude product was purified by silica gel chromatography (hexane/EtOAc = 5/1) to afford **2l** (49.9 mg, 94%): pale yellow oil; ^1^H NMR (400 MHz, CDCl_3_) δ 7.32–7.25 (m, 4H), 7.19–7.14 (m, 1H), 3.63 (s, 1H), 3.06 (d, *J* = 17.5 Hz, 1H), 2.75 (d, *J* = 17.5 Hz, 1H), 1.82–1.76 (m, 1H), 1.68–1.62 (m, 1H), 1.54 (s, 3H), 1.53–1.44 (m, 1H), 1.28 (s, 3H), 1.22 (s, 3H), 0.83 (d, *J* = 6.5 Hz, 3H), 0.58 (d, *J* = 6.8 Hz, 3H); ^13^C­{^1^H} NMR (100 MHz, CDCl_3_) δ 212.5, 146.8, 128.0, 126.0, 125.8, 76.2, 52.1, 48.1, 40.4, 26.3, 26.3, 25.2, 24.6, 24.3, 23.9; [α]^26.0^_D_ (95% ee) = – 6.10 (*c* 1.15, CHCl_3_); HRMS (APCI, positive) *m*/*z*: [M–CH_3_]^+^ calcd. for C_16_H_23_O_2_^+^ 247.1693; found 247.1691; HPLC (CHIRALPAK IC, Daicel, 4.6 × 250 mm, hexane/^*i*^PrOH = 99/1, 1.0 mL/min, 210 nm), t_r_ (major) = 8.90 min, t_r_ (minor) = 11.1 min.

**2-Hydroxy-2,5-dimethyl-5,6-diphenylhexan-3-one (2m)**: CuI (1.9 mg, 0.01 mmol) and **BmP-F** (10.1 mg, 0.02 mmol) were dissolved in THF (0.2 mL) and the mixture was stirred for 30 min, then cooled with ice bath. The solution of Me_3_Al in hexane (0.57 mL, 0.80 mmol, 1.4 M) was added dropwise. To the resulting solution was added (*E*)-**1m** (0.20 mmol, 56.1 mg, 52 μL, *d* = 1.07) at once. The reaction was carried out at 40 °C and monitored by TLC. After 24 h, a minimal amount of sat. NH_4_Cl aq. was added at 0 °C, then stirred for 30 min. The mixture was filtered through silica gel pad, washed with Et_2_O and concentrated. The crude product was purified by silica gel chromatography (hexane/EtOAc = 5/1) to afford **2m** (45.2 mg, 76%): colorless oil; ^1^H NMR (400 MHz, CDCl_3_) δ 7.33–7.11 (m, 8H), 6.82–6.78 (m, 2H), 3.63 (s, 1H), 3.17–3.05 (m, 3H), 2.84 (d, *J* = 18.0 Hz, 1H), 1.44 (s, 3H), 1.28 (d, *J* = 1.8 Hz, 6H); ^13^C­{^1^H} NMR (100 MHz, CDCl_3_) δ 212.6, 146.5, 137.7, 130.5, 128.1, 127.7, 126.3, 126.0, 126.0, 77.5, 76.2, 48.7, 45.1, 40.8, 26.5, 24.9; [α]^26.0^_D_ (95% ee) = +15.1 (*c* 1.00, CHCl_3_); HRMS (APCI, positive) *m*/*z*: [M + H]^+^ calcd. for C_20_H_25_O_2_^+^ 297.1849; found 297.1850; HPLC (CHIRALPAK IC, Daicel, 4.6 × 250 mm, hexane/^*i*^PrOH = 99/1, 1.0 mL/min, 210 nm), t_r_ (major) = 14.5 min, t_r_ (minor) = 16.1 min.

**1-(1-Hydroxycyclopentyl)-3-methyl-3-phenylpentan-1-one (2n)**: CuI (1.9 mg, 0.01 mmol) and **BmP-F** (10.1 mg, 0.02 mmol) were dissolved in THF (0.2 mL) and the mixture was stirred for 30 min, then cooled with ice bath. The solution of Me_3_Al in hexane (0.57 mL, 0.80 mmol, 1.4 M) was added dropwise. To the resulting solution was added (*E*)-**1n** (0.20 mmol, 48.9 mg, 46 μL, *d* = 1.06) at once. The reaction was carried out at 40 °C and monitored by TLC. After 24 h, a minimal amount of sat. NH_4_Cl aq. was added at 0 °C, then stirred for 30 min. The mixture was filtered through silica gel pad, washed with Et_2_O and concentrated. The crude product was purified by silica gel chromatography (hexane/EtOAc = 5/1) to afford **2n** (40.3 mg, 76%): colorless oil; ^1^H NMR (400 MHz, CDCl_3_) δ 7.35–7.26 (m, 4H), 7.21–7.15 (m, 1H), 3.57 (s, 1H), 2.99 (d, *J* = 17.0 Hz, 1H), 2.76 (d, *J* = 16.8 Hz, 1H), 1.97–1.65 (m, 8H), 1.57–1.43 (m, 5H), 0.69 (t, *J* = 7.6 Hz, 3H); ^13^C­{^1^H} NMR (100 MHz, CDCl_3_) δ 212.6, 146.4, 128.1, 126.0, 125.8, 87.3, 47.3, 40.7, 38.6, 38.6, 35.2, 25.1, 23.4, 8.4 (One carbon atom was not found probably due to overlapping); [α]^26.0^_D_ (84% ee) = +6.17 (*c* 1.09, CHCl_3_); HRMS (APCI, positive) *m*/*z*: [M–OH]^+^ calcd. for C_17_H_23_O^+^ 243.1743; found 243.1738; HPLC (CHIRALPAK IB, Daicel, 4.6 × 150 mm, hexane/^*i*^PrOH = 99/1, 1.0 mL/min, 205 nm), t_r_ (major) = 4.18 min, t_r_ (minor) = 4.72 min.

**1-(1-Hydroxycyclohexyl)-3-methyl-3-phenylpentan-1-one (2o)**: CuI (1.9 mg, 0.01 mmol) and **BmP-F** (10.1 mg, 0.02 mmol) were dissolved in THF (0.2 mL) and the mixture was stirred for 30 min, then cooled with ice bath. The solution of Me_3_Al in hexane (0.57 mL, 0.80 mmol, 1.4 M) was added dropwise. To the resulting solution was added (*E*)-**1o** (0.20 mmol, 51.7 mg, 49 μL, *d* = 1.05) at once. The reaction was carried out at 40 °C and monitored by TLC. After 24 h, a minimal amount of sat. NH_4_Cl aq. was added at 0 °C, then stirred for 30 min. The mixture was filtered through silica gel pad, washed with Et_2_O and concentrated. The crude product was purified by silica gel chromatography (hexane/EtOAc = 10/1) to afford **2o** (30.0 mg, 54%): colorless oil; ^1^H NMR (400 MHz, CDCl_3_) δ 7.30–7.24 (m, 4H), 7.19–7.15 (m, 1H), 3.40 (s, 1H), 3.08 (d, *J* = 17.5 Hz, 1H), 2.79 (d, *J* = 17.5 Hz, 1H), 1.90–1.52 (m, 9H), 1.46 (s, 3H), 1.37–1.16 (m, 3H), 0.68 (t, *J* = 7.6 Hz, 3H); ^13^C­{^1^H} NMR (100 MHz, CDCl_3_) δ 213.0, 146.7, 128.0, 125.9, 125.7, 78.1, 47.0, 40.3, 35.5, 33.7, 25.3, 23.5, 21.1, 8.4 (Two carbon atoms were not found probably due to overlapping); [α]^26.0^_D_ (69% ee) = +9.61 (*c* 1.09, CHCl_3_); HRMS (APCI, positive) *m*/*z*: [M + H]^+^ calcd. for C_18_H_27_O_2_^+^ 275.2006; found 275.2001; HPLC (CHIRALPAK IC, Daicel, 4.6 × 250 mm, hexane/^*i*^PrOH = 99/1, 1.0 mL/min, 210 nm), t_r_ (major) = 7.92 min, t_r_ (minor) = 12.4 min.

**(R)-3-Methyl-3-phenylpentanoic acid (8)**: To a solution of **2a** (0.20 mmol, 46.9 mg, 47 μL, *d* = 0.99) in MeOH (0.5 mL) was added a solution of NaIO_4_ (0.43 g, 2.0 mmol) in H_2_O (5.0 mL). The reaction mixture was stirred at rt for 24 h, then MeOH was removed under reduced pressure and, CH_2_Cl_2_ (5.0 mL) and 1 M HCl aq. (2.0 mL) were added. The aqueous layer was extracted with CH_2_Cl_2_ (5 mL × 3). The combined organic layers were dried over anh. Na_2_SO_4_, filtered, and concentrated to afford **8** (37.2 mg, >99%): yellow oil; [α]^26.0^_D_ = – 13.0 (*c* 1.31, CHCl_3_); The NMR spectra matched the previous report.^14^ The determination of enantiomer excess: A mixture of **8** (0.19 mmol, 37.2 mg), MeOH (0.5 mL) and catalytic amount of conc. H_2_SO_4_ was refluxed for 2 h. The mixture was concentrated to remove MeOH, then EtOAc (1 mL), and sat. NaHCO_3_ aq. (1 mL) were added. The aqueous layer was extracted with EtOAc (1 mL × 2). The combined organic layers were dried over anh. Na_2_SO_4_, filtered, and concentrated to afford the methyl ester. The crude product was used in the next step without further purification. To a solution of the crude methyl ester in Et_2_O (1.0 mL) at −78 °C was added dropwise a solution of DIBAL-H in hexane (0.40 mmol, 0.4 mL, 1.0 M). The reaction mixture was stirred at −78 °C for 1 h, then quenched with a minimal amount of H_2_O, filtered through a pad of Celite, washed with Et_2_O and concentrated. The crude product was purified by silica gel chromatography (hexane/EtOAc = 10/1) to afford the corresponding aldehyde **9** (7.2 mg, 21% in 2 steps).

**3-Methyl-3-phenylpentanal (9)**: To a solution of **2a** (0.20 mmol, 46.9 mg, 47 μL, *d* = 0.99) in MeOH (1.5 mL) at 0 °C, NaBH_4_ (11.3 mg, 0.30 mmol) was added. The reaction mixture was stirred at this temperature for 2 h. The reaction mixture was quenched with 1 M HCl aq. and extracted with CH_2_Cl_2_ (6 mL × 3). The combined organic layers were dried over anh. Na_2_SO_4_, filtered, and concentrated. The crude product was used for next step without further purification. To a solution of the crude product in MeOH (0.5 mL) was added a solution of NaIO_4_ (0.43 g, 2.0 mmol) in H_2_O (5.0 mL). The reaction mixture was stirred at rt for 24 h, then MeOH was removed under reduced pressure. The aqueous layer was extracted with CH_2_Cl_2_ (5 mL × 3). The combined organic layers were dried over anh. Na_2_SO_4_, filtered, and concentrated. The crude product was purified by silica gel chromatography (hexane/EtOAc = 10/1) to afford **9** (27.2 mg, 76%): colorless oil; ^1^H NMR (400 MHz, CDCl_3_) δ 9.50 (dd, *J* = 3.5, 2.5 Hz, 1H), 7.37–7.31 (m, 4H), 7.25–7.19 (m, 1H), 2.81 (ddd, *J* = 15.3, 2.5, 0.5 Hz, 1H), 2.53 (dd, *J* = 15.1, 3.5 Hz, 1H), 1.84 (dq, *J* = 13.9, 7.6 Hz, 1H), 1.69 (dq, *J* = 13.9, 7.5 Hz, 1H), 1.46 (s, 3H), 0.71 (t, *J* = 7.6 Hz, 3H); ^13^C­{^1^H} NMR (100 MHz, CDCl_3_) δ 203.3, 145.6, 128.4, 126.1, 55.1, 40.1, 36.0, 24.3, 8.3 (One carbon atom was not found probably due to overlapping); [α]^24.0^_D_ (98% ee) = – 20.5 (*c* 1.14, CHCl_3_); HRMS (APCI, positive) *m*/*z*: [M + H]^+^ calcd. for C_12_H_17_O^+^ 177.1274; found 177.1277; HPLC (CHIRALPAK IB, Daicel, 4.6 × 150 mm, hexane/^*i*^PrOH = 99.9/0.1, 1.0 mL/min, 210 nm), t_r_ (minor) = 4.67 min, t_r_ (major) = 5.11 min. The aldehyde **9** derived from carboxylic acid **8** was obtained as 96% ee (−).

## Supplementary Material



## Data Availability

The data underlying this study are available in the published article and the Supporting Information.

## Notes

General information; synthesis and physical properties of new compound; NMR spectra for all compounds; and HPLC spectra (PDF)
