# Supplementary material for: Hydroxy Group-Assisted Cu-Catalyzed Asymmetric Conjugate Addition for the Creation of an All-Carbon Quaternary Center
Source: J Org Chem. 2026 May 18;91(21):7356–69. doi: 10.1021/acs.joc.6c00144 (PMC13227467; doi:10.1021/acs.joc.6c00144)

Supporting Information

Hydroxy Group Assisted Cu-Catalyzed Asymmetric Conjugate Addition for Creation of an All-Carbon Quaternary Center

Taiyo Yamamoto, Yuma Shiratori, Shogo Yamaguchi, Ken Yamanomoto, Kohei Endo*

* email: kendo@rs.tus.ac.jp

Department of Chemistry, Faculty of Science, Tokyo University of Science, Shinjuku, Tokyo 162-8601, Japan

Contents:

1. Optimization Study **S2**
2. Control experiments **S3**
3. Chiral HPLC Charts **S5**
4. NMR Chart of New Compounds **S13**

1. Optimization Study

Table S1. Optimization of Cu-salt

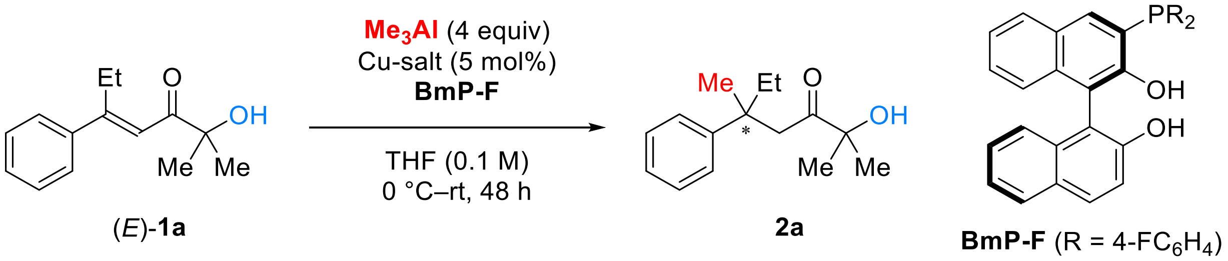

Entry[a]	Cu-salt	Yield / %	Ee / %[b]
1	CuI	83	90
2	CuBr	78	18
3	CuCl	23	86
4	CuOAc	trace	-
5	$CuCl_2 \cdot 2H_2O$	30	33
6	$CuBr_2$	72	9

[a] Me_3Al (1.4 M in hexane) was used. [b] Ee was determined by chiral HPLC analysis.

Table S2. Optimization of concentration and temperature

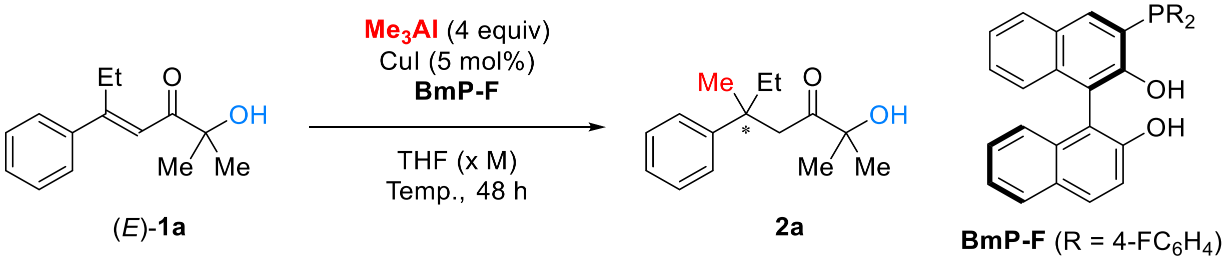

Entry[a]	x / M	Temp. / °C	Yield / %	Ee / %[b]
1	0.1	0 °C–rt	83	90
2	0.2	0 °C–rt	81	88
3	1.0	0 °C–rt	74	92
4	1.0	0 °C	trace	-
5[b]	1.0	0 °C–40 °C	81	96
6[b]	1.0	0 °C–50 °C	85	94

[a] Me_3Al (1.4 M in hexane) was used. [b] Ee was determined by chiral HPLC analysis. (b) 24 hours.

2. Control experiments

Table S3. Control experiments

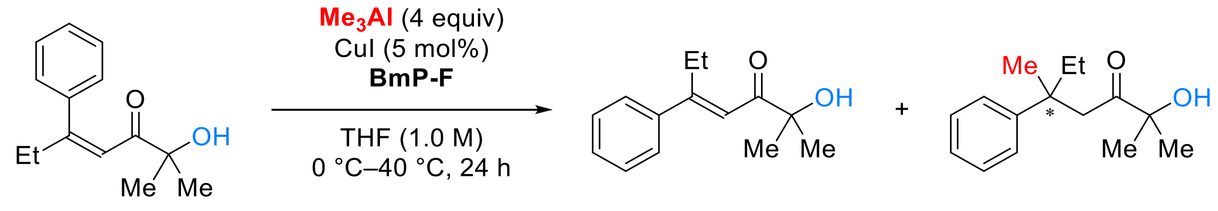

(*Z*)-**1a** (*E*)-**1a** **2a**

Entry[a]	Conditions	Conv. / %[b]	NMR Yield / %[b]	
			(*E*)-**1a**	**2a**
1	w/o Me_3Al	0	0	0
2	w/o CuI	44	55	0
3	cat. Me_3Al	79	20	0
4	w/o Me_3Al, CuI	0	0	0
5	w/o **BmP-F**	70	9	60

[a] Me_3Al (1.4 M in hexane) was used. [b] Determined by ^1H NMR analysis of the crude product. CH_2Br_2 was used as an internal standard.

Table S4. Time-dependent comparison of yields between optimized and ligand-free conditions

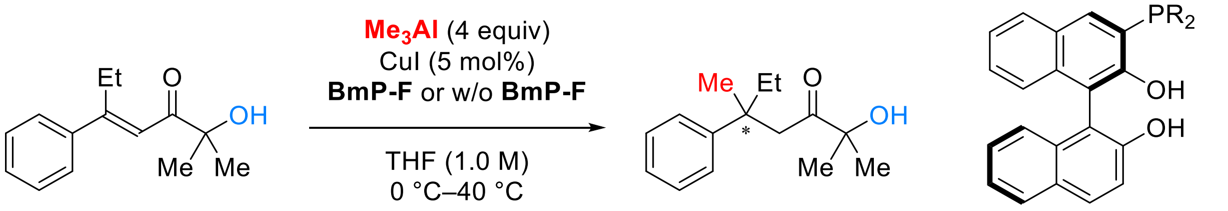

(*E*)-**1a** **2a** **BmP-F** (R = 4-FC_6H_4)

Time / h[a]	NMR Yield / %[b]	
	Optimized conditions	Ligand-free conditions
1	44	9
2	53	11
4	90	20
8	89	48

[a] Me_3Al (1.4 M in hexane) was used. [b] Determined by ^1H NMR analysis of the crude product. CH_2Br_2 was used as an internal standard.

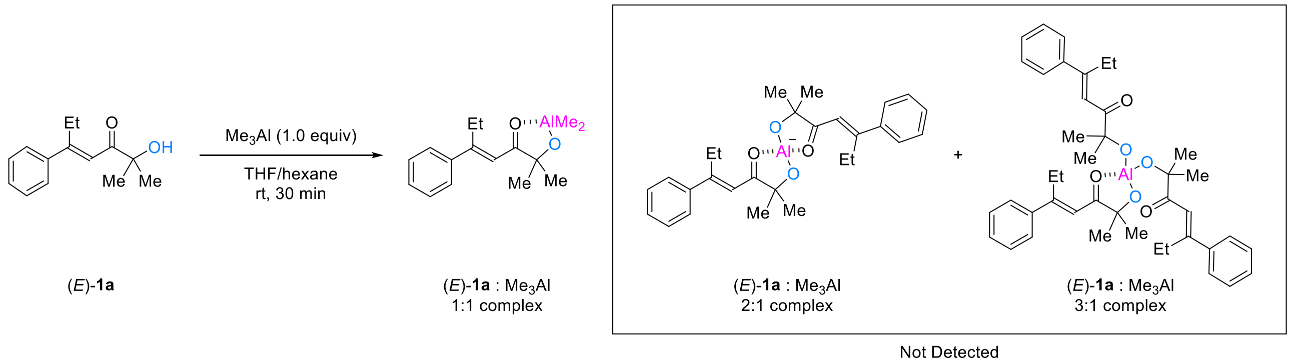

^{1}H NMR (CD_2Cl_2, 400 MHz)

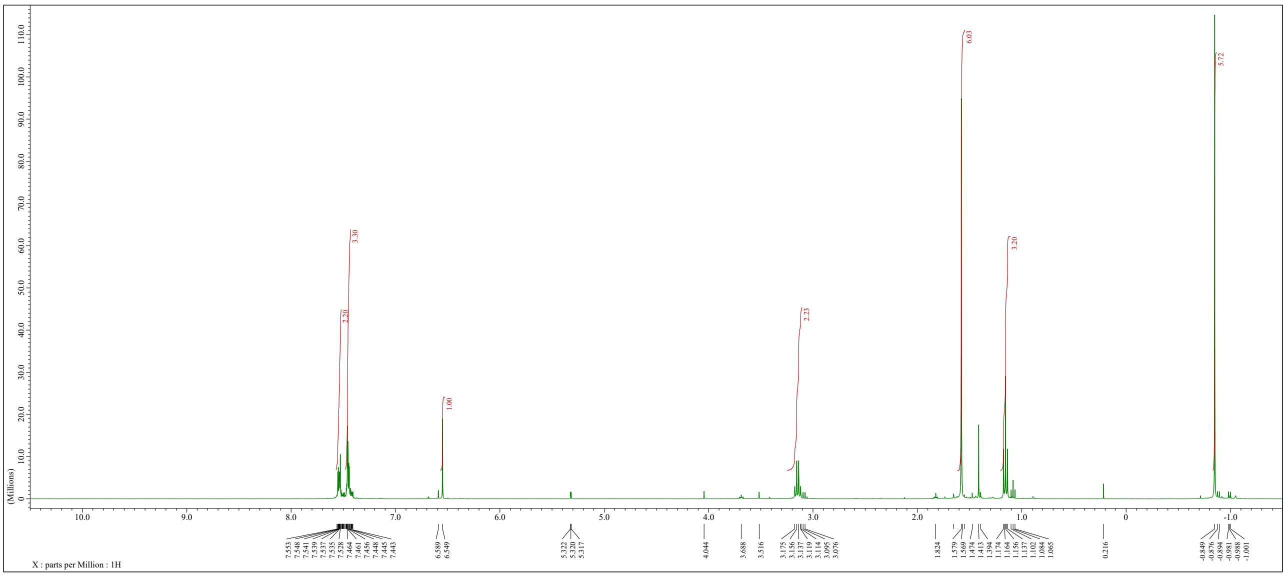

Figure S1. NMR spectra of deprotonated (*E*)-1a with Me_3Al

Experimental procedure: To a solution of Me_3Al in hexane (0.10 mmol, 70 μL, 1.4 M) in THF (0.1 mL) at 0 °C was added (*E*)-**1a** (0.1 mmol, 21.8 mg, 21 μL, d = 1.03) at once. The reaction mixture was stirred at rt for 30 minutes, then volatiles were removed in *vacuo*. The resulting white precipitates were dissolved in CD_2Cl_2 (0.5 mL) and transferred into an NMR tube, then ^{1}H NMR analysis was performed.

In the ^{1}H NMR spectra, the peak attributed to the proton of the hydroxy group at (*E*)-**1a** disappeared. The integration ratio suggests the generation of Me_2Al-complex like the 1:1 complex as the reasonable intermediate in the reaction mechanism.

3. Chiral HPLC Charts

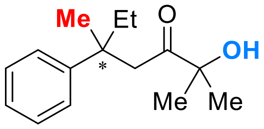

Chiral HPLC Chart (racemic)

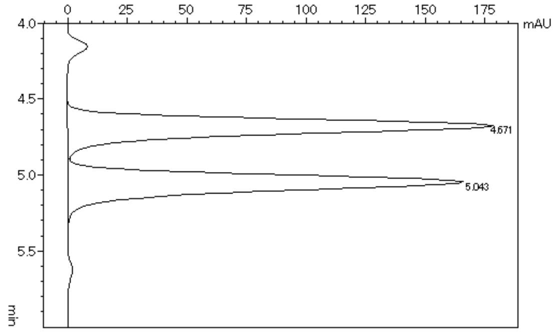

CALCULATION REPORT

PDA Ch1 210nm 4nm

Peak#	Ret. Time	Area	Height	Area %
1	4.671	1234572	178875	50.042
2	5.043	1232520	165756	49.958
Total		2467092	344632	100.000

Chiral HPLC Chart (96% ee)

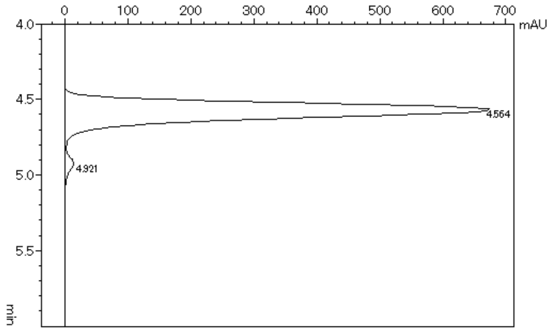

CALCULATION REPORT

PDA Ch1 210nm 4nm

Peak#	Ret. Time	Area	Height	Area %
1	4.564	4660200	674495	97.798
2	4.921	104946	13894	2.202
Total		4765145	688389	100.000

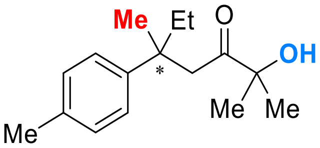

Chiral HPLC Chart (racemic)

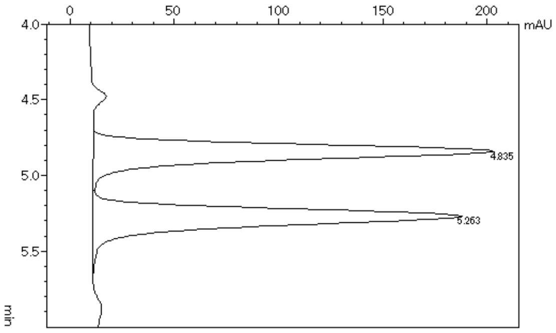

CALCULATION REPORT

PDA Ch1 210nm 4nm

Peak#	Ret. Time	Area	Height	Area %
1	4.835	1339494	192361	49.790
2	5.263	1350819	176718	50.210
Total		2690313	369079	100.000

Chiral HPLC Chart (96% ee)

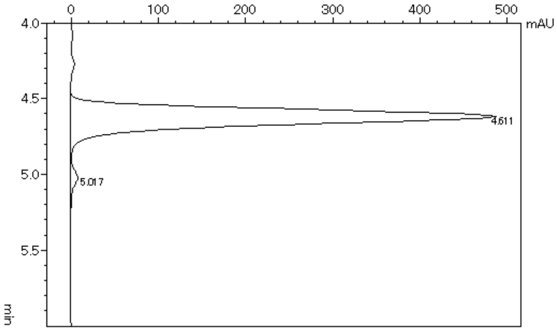

CALCULATION REPORT

PDA Ch1 210nm 4nm

Peak#	Ret. Time	Area	Height	Area %
1	4.611	3350612	489216	98.093
2	5.017	65133	8080	1.907
Total		3415744	497296	100.000

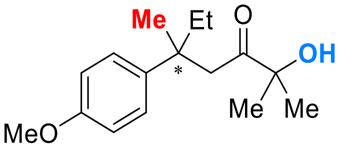

Chiral HPLC Chart (racemic)

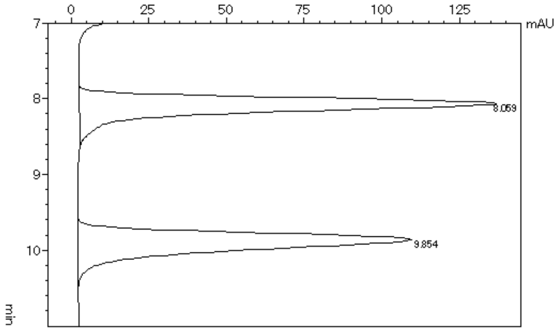

CALCULATION REPORT

PDA Ch1 210nm 4nm

Peak#	Ret. Time	Area	Height	Area %
1	8.059	1689508	134336	50.247
2	9.854	1672918	107381	49.753
Total		3362426	241718	100.000

Chiral HPLC Chart (96% ee)

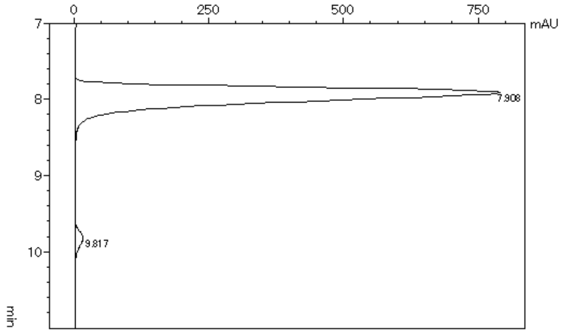

CALCULATION REPORT

PDA Ch1 210nm 4nm

Peak#	Ret. Time	Area	Height	Area %
1	7.908	10335300	790795	97.848
2	9.817	227303	15082	2.152
Total		10562603	805876	100.000

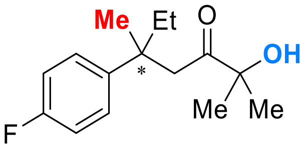

Chiral HPLC Chart (racemic)

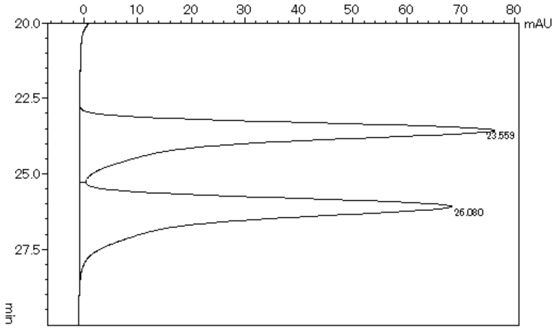

CALCULATION REPORT

PDA Ch1 210nm 4nm

Peak#	Ret. Time	Area	Height	Area %
1	23.559	3542419	77054	49.788
2	26.080	3572544	69061	50.212
Total		7114963	146115	100.000

Chiral HPLC Chart (94% ee)

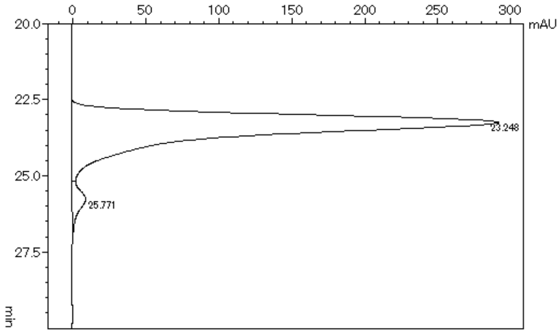

CALCULATION REPORT

PDA Ch1 210nm 4nm

Peak#	Ret. Time	Area	Height	Area %
1	23.248	13591189	292991	96.753
2	25.771	456087	9244	3.247
Total		14047276	302235	100.000

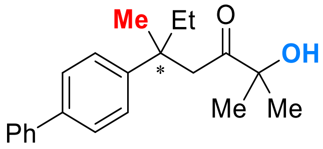

Chiral HPLC Chart (racemic)

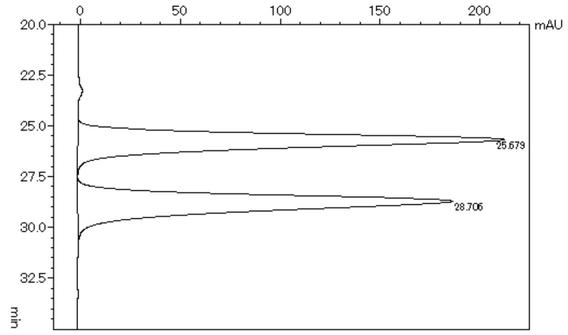

CALCULATION REPORT

PDA Ch1 210nm 4nm

Peak#	Ret. Time	Area	Height	Area %
1	25.679	9897662	213843	50.196
2	28.706	9820439	187475	49.804
Total		19718101	401318	100.000

Chiral HPLC Chart (97% ee)

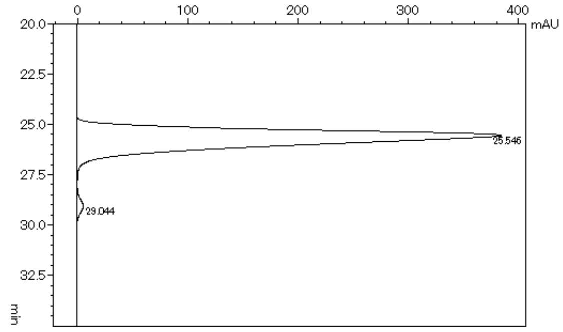

CALCULATION REPORT

PDA Ch1 210nm 4nm

Peak#	Ret. Time	Area	Height	Area %
1	25.546	20007023	385499	98.674
2	29.044	268813	5724	1.326
Total		20275836	391223	100.000

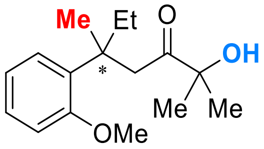

Chiral HPLC Chart (racemic)

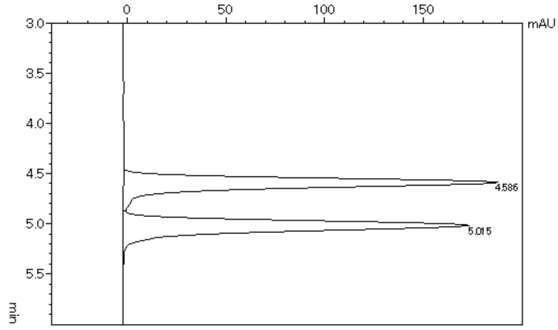

CALCULATION REPORT

PDA Ch1 210nm 4nm

Peak#	Ret. Time	Area	Height	Area %
1	4.586	1268515	189901	50.239
2	5.015	1256432	174934	49.761
Total		2524947	364835	100.000

Chiral HPLC Chart (92% ee)

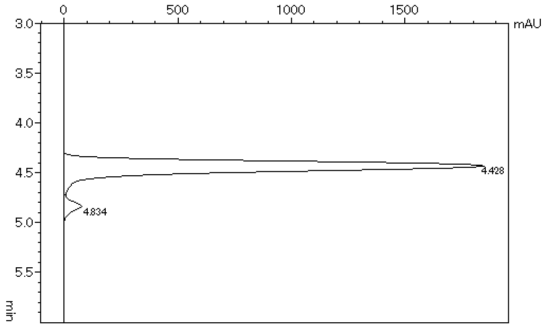

CALCULATION REPORT

PDA Ch1 210nm 4nm

Peak#	Ret. Time	Area	Height	Area %
1	4.428	13150800	1851879	95.912
2	4.834	560503	77947	4.088
Total		13711304	1929826	100.000

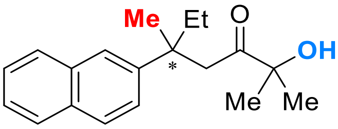

Chiral HPLC Chart (racemic)

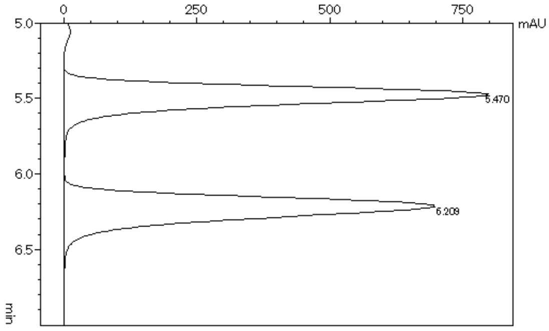

CALCULATION REPORT

PDA Ch1 210nm 4nm

Peak#	Ret. Time	Area	Height	Area %
1	5.470	6723858	798999	49.907
2	6.209	6748959	696799	50.093
Total		13472817	1495798	100.000

Chiral HPLC Chart (94% ee)

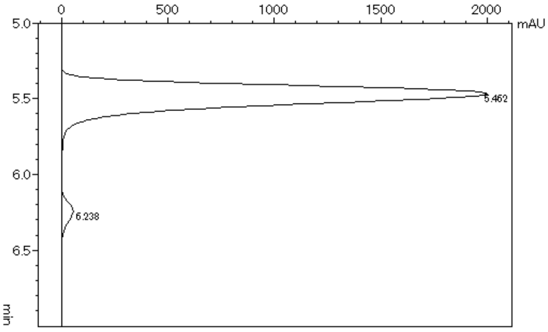

CALCULATION REPORT

PDA Ch1 210nm 4nm

Peak#	Ret. Time	Area	Height	Area %
1	5.462	17738198	2003200	97.132
2	6.238	523845	54763	2.868
Total		18262042	2057963	100.000

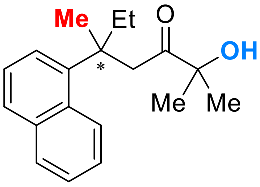

Chiral HPLC Chart (racemic)

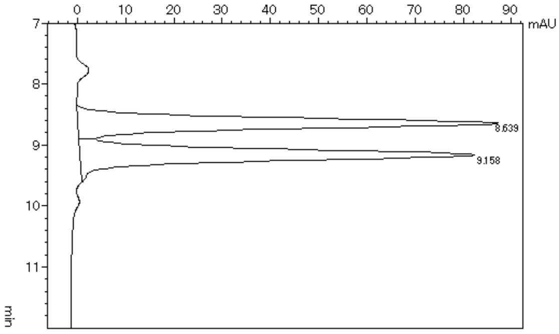

CALCULATION REPORT

PDA Ch1 210nm 4nm

Peak#	Ret. Time	Area	Height	Area %
1	8.639	1053096	87307	49.840
2	9.158	1059871	81865	50.160
Total		2112967	169172	100.000

Chiral HPLC Chart (94% ee)

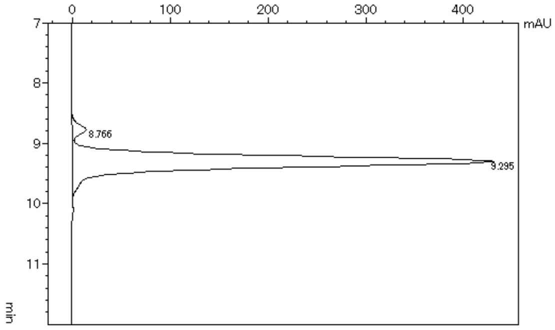

CALCULATION REPORT

PDA Ch1 210nm 4nm

Peak#	Ret. Time	Area	Height	Area %
1	8.766	171341	14259	2.862
2	9.295	5816126	431521	97.138
Total		5987468	445780	100.000

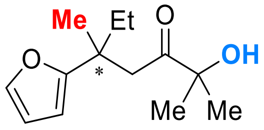

Chiral HPLC Chart (racemic)

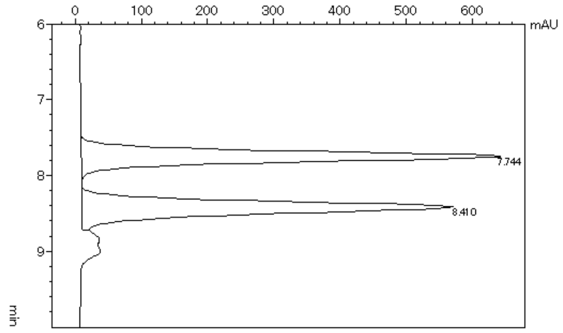

CALCULATION REPORT

PDA Ch1 210nm 4nm

Peak#	Ret. Time	Area	Height	Area %
1	7.744	6413374	633506	50.041
2	8.410	6402837	561203	49.959
Total		12816211	1194709	100.000

Chiral HPLC Chart (95% ee)

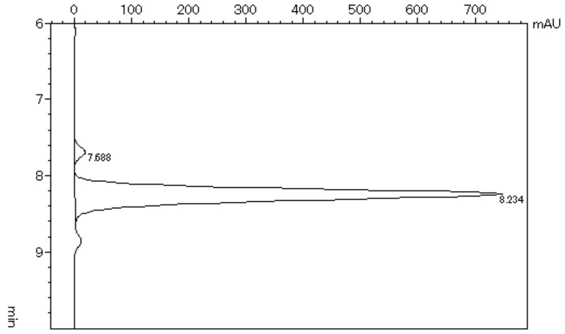

CALCULATION REPORT

PDA Ch1 210nm 4nm

Peak#	Ret. Time	Area	Height	Area %
1	7.688	192691	19031	2.293
2	8.234	8210812	747286	97.707
Total		8403503	766317	100.000

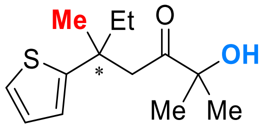

Chiral HPLC Chart (racemic)

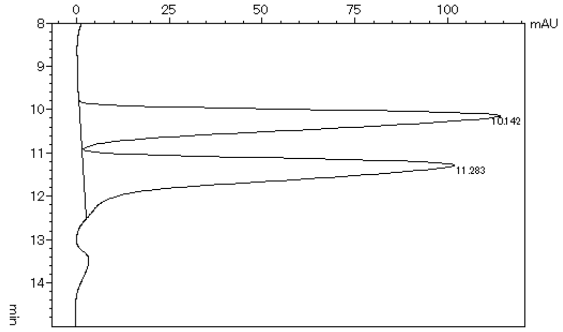

CALCULATION REPORT

PDA Ch1 210nm 4nm

Peak#	Ret. Time	Area	Height	Area %
1	10.142	3379979	113459	49.697
2	11.283	3421171	100059	50.303
Total		6801150	213518	100.000

Chiral HPLC Chart (97% ee)

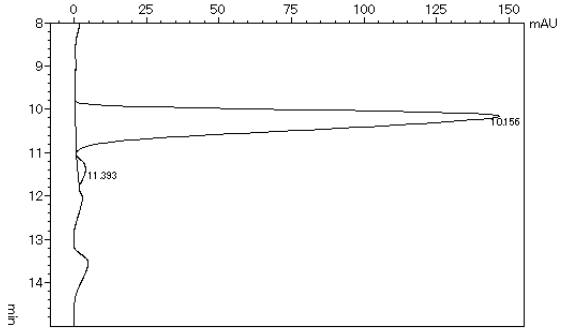

CALCULATION REPORT

PDA Ch1 210nm 4nm

Peak#	Ret. Time	Area	Height	Area %
1	10.156	4403893	146329	98.291
2	11.393	76567	2792	1.709
Total		4480460	149121	100.000

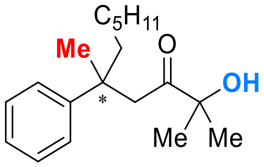

Chiral HPLC Chart (racemic)

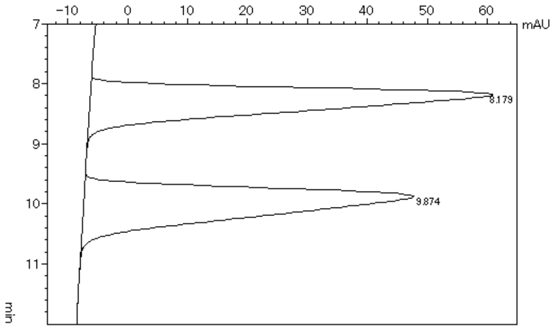

Chiral HPLC Chart (94% ee)

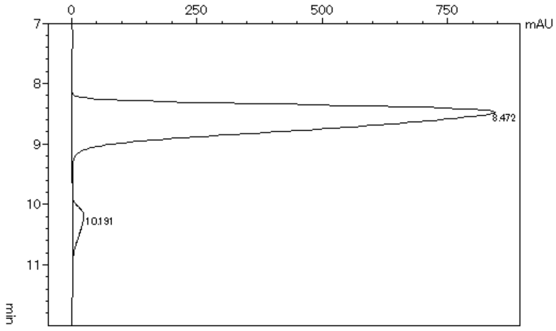

CALCULATION REPORT

PDA Ch1 210nm 4nm

Peak#	Ret. Time	Area	Height	Area %
1	8.179	1708055	67280	49.991
2	9.874	1708643	55076	50.009
Total		3416698	122356	100.000

CALCULATION REPORT

PDA Ch1 210nm 4nm

Peak#	Ret. Time	Area	Height	Area %
1	8.472	22923216	844469	96.951
2	10.191	720805	22895	3.049
Total		23644021	867364	100.000

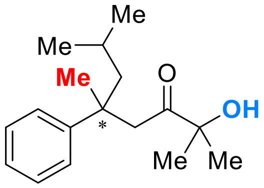

Chiral HPLC Chart (racemic)

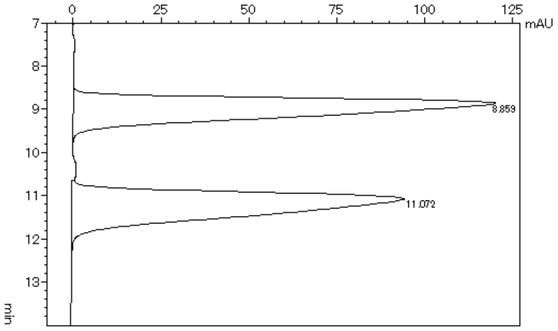

Chiral HPLC Chart (95% ee)

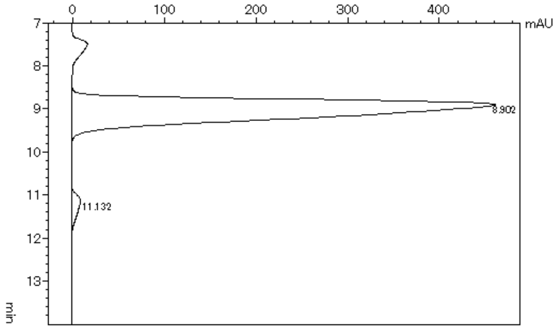

CALCULATION REPORT

PDA Ch1 210nm 4nm

Peak#	Ret. Time	Area	Height	Area %
1	8.859	3344252	120126	50.059
2	11.072	3336380	94404	49.941
Total		6680632	214530	100.000

CALCULATION REPORT

PDA Ch1 210nm 4nm

Peak#	Ret. Time	Area	Height	Area %
1	8.902	13024329	462162	97.538
2	11.132	328685	9502	2.462
Total		13353014	471664	100.000

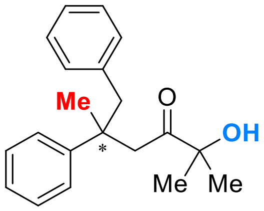

Chiral HPLC Chart (racemic)

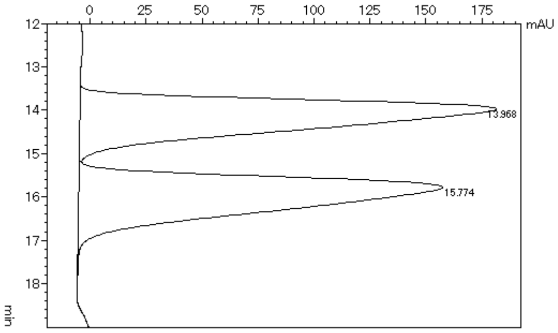

CALCULATION REPORT

PDA Ch1 210nm 4nm

Peak#	Ret. Time	Area	Height	Area %
1	13.968	8328157	186321	49.919
2	15.774	8355305	162872	50.081
Total		16683462	349193	100.000

Chiral HPLC Chart (95% ee)

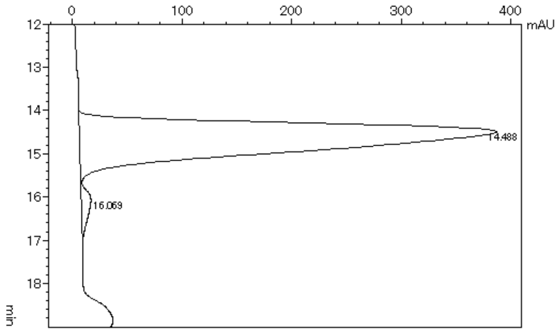

CALCULATION REPORT

PDA Ch1 210nm 4nm

Peak#	Ret. Time	Area	Height	Area %
1	14.488	15848066	381489	97.528
2	16.069	401696	8901	2.472
Total		16249762	390390	100.000

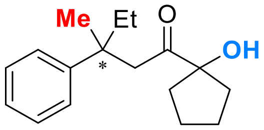

Chiral HPLC Chart (racemic)

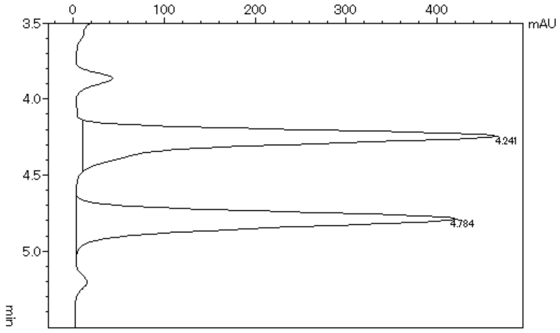

CALCULATION REPORT

PDA Ch1 205nm 4nm

Peak#	Ret. Time	Area	Height	Area %
1	4.241	3059272	457863	50.220
2	4.784	3032414	419282	49.780
Total		6091686	877145	100.000

Chiral HPLC Chart (84% ee)

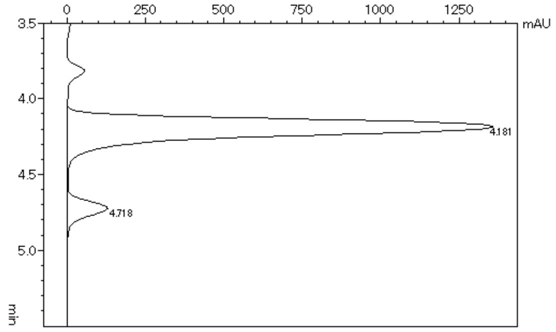

CALCULATION REPORT

PDA Ch1 210nm 4nm

Peak#	Ret. Time	Area	Height	Area %
1	4.181	10367642	1356399	92.061
2	4.718	894015	128597	7.939
Total		11261657	1484996	100.000

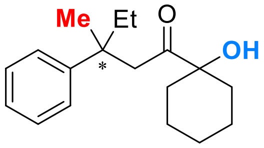

Chiral HPLC Chart (racemic)

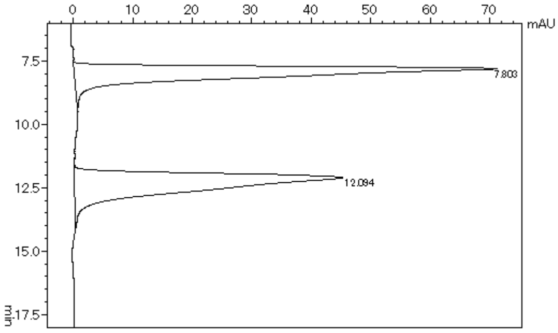

CALCULATION REPORT

PDA Ch1 210nm 4nm

Peak#	Ret. Time	Area	Height	Area %
1	7.803	1875615	71176	50.100
2	12.094	1868133	45003	49.900
Total		3743747	116179	100.000

Chiral HPLC Chart (69% ee)

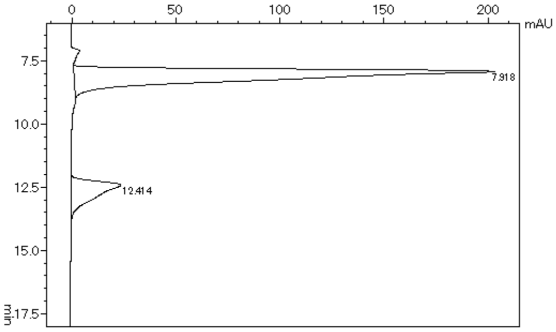

CALCULATION REPORT

PDA Ch1 210nm 4nm

Peak#	Ret. Time	Area	Height	Area %
1	7.918	5256354	202628	84.283
2	12.414	980180	23795	15.717
Total		6236534	226423	100.000

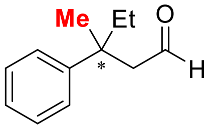

Chiral HPLC Chart (racemic)

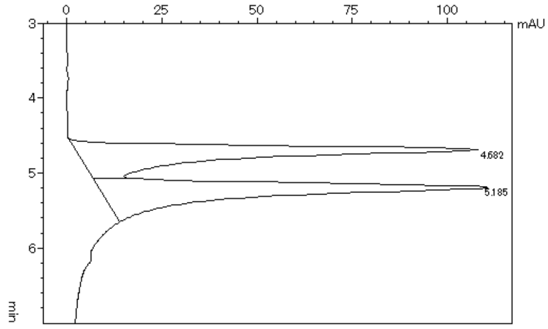

CALCULATION REPORT

PDA Ch1 210nm 4nm

Peak#	Ret. Time	Area	Height	Area %
1	4.682	1198353	105796	49.578
2	5.185	1218744	102298	50.422
Total		2417097	208094	100.000

Chiral HPLC Chart (98% ee)

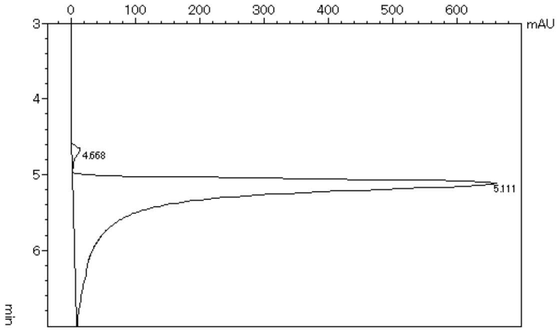

CALCULATION REPORT

PDA Ch1 210nm 4nm

Peak#	Ret. Time	Area	Height	Area %
1	4.668	143684	14049	1.216
2	5.111	11669560	660393	98.784
Total		11813244	674442	100.000

4. NMR Chart of New Compounds

^{1}H NMR (400 MHz, $CDCl_3$) **BmP-CF$_3$**

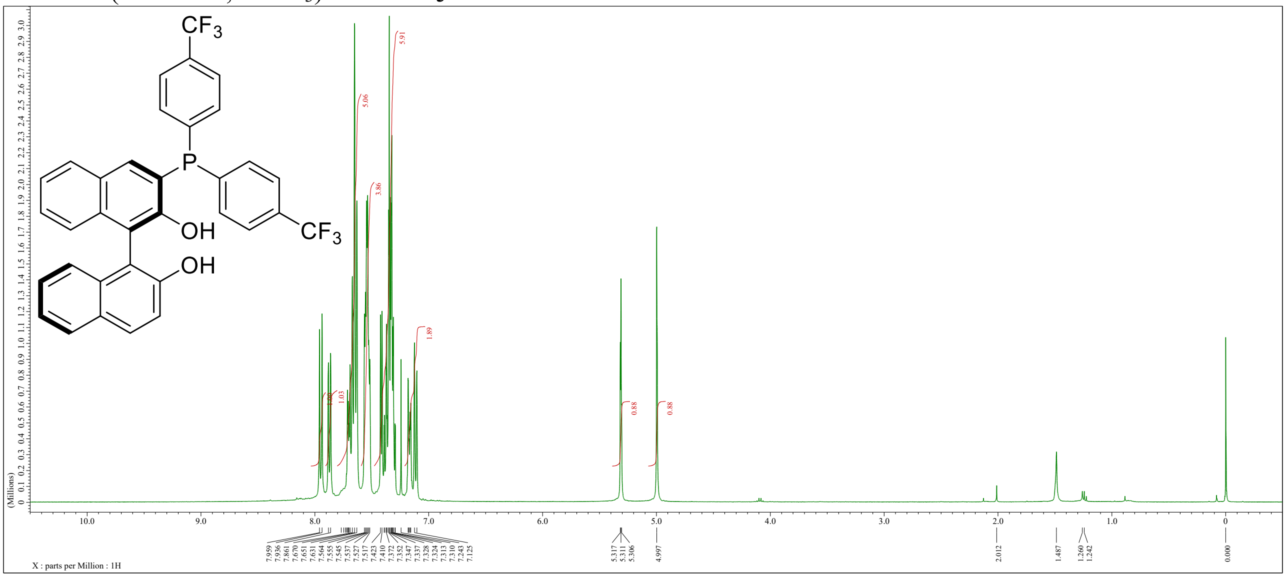

^{13}C{^{1}H} NMR (100 MHz, $CDCl_3$) **BmP-CF$_3$**

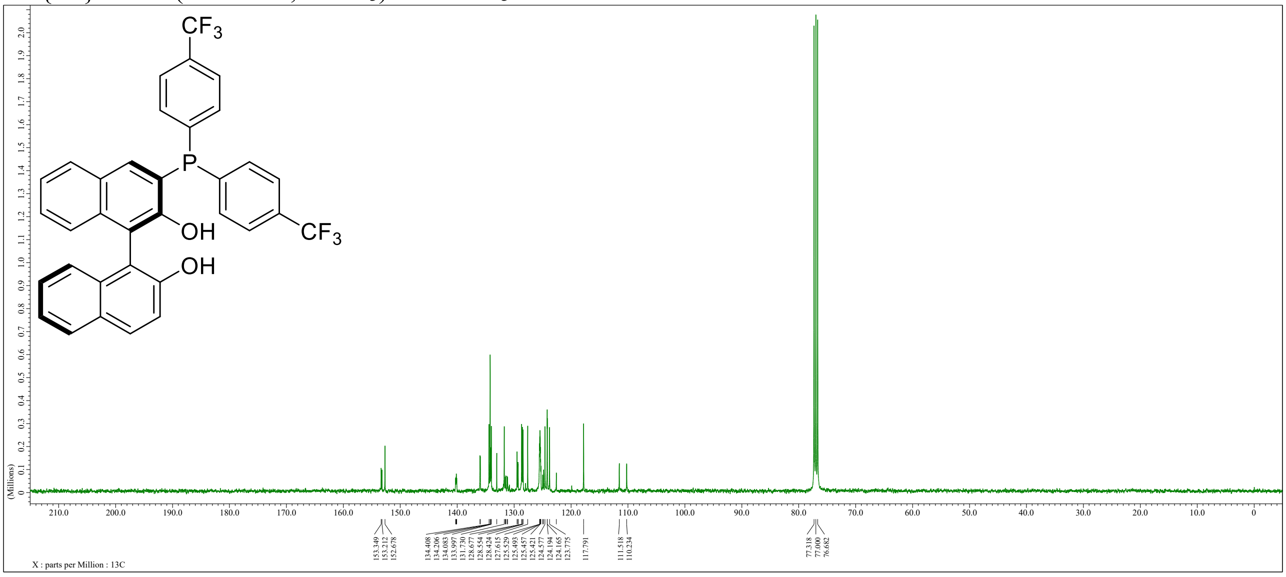

^{19}F NMR (375 MHz, $CDCl_3$) **BmP-CF$_3$**

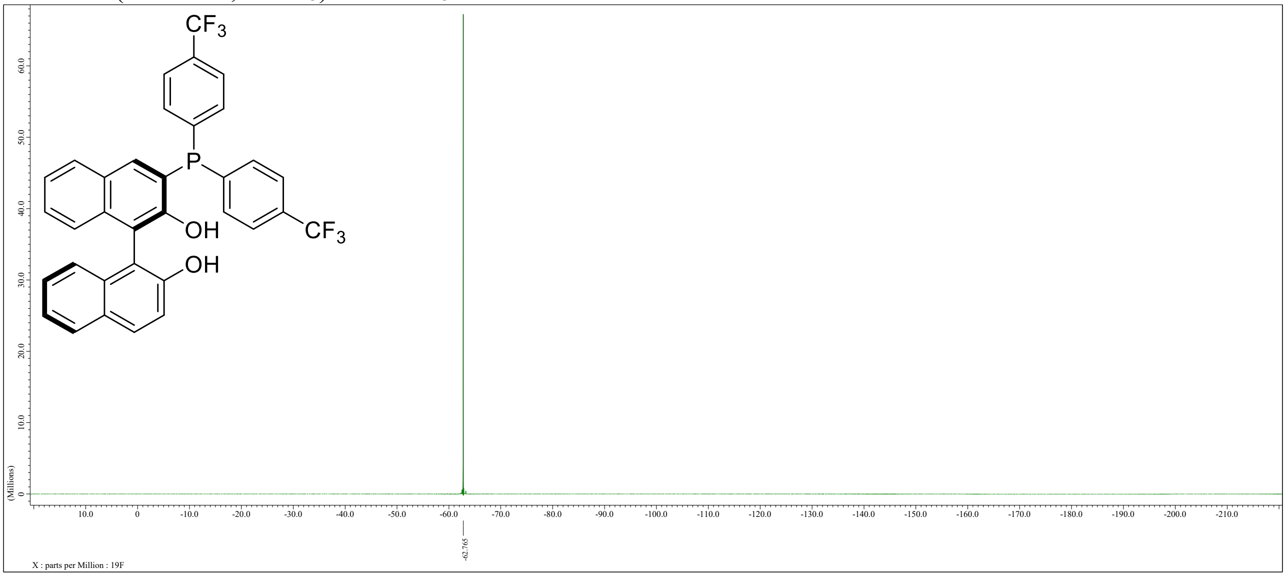

^{31}P NMR (162 MHz, $CDCl_3$) **BmP-CF$_3$**

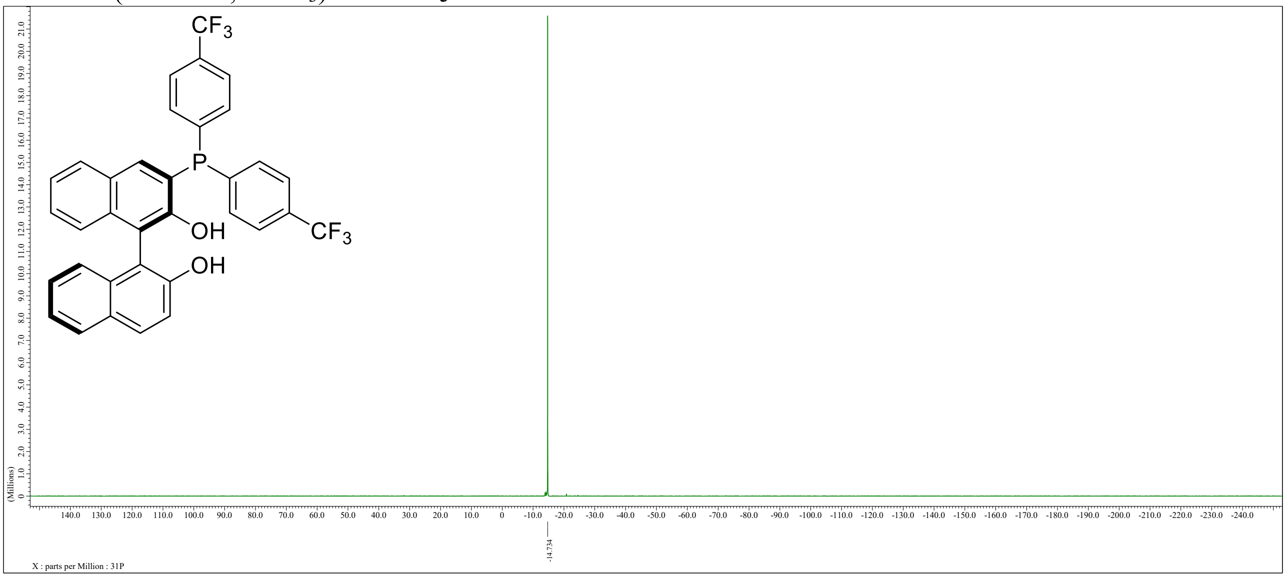

^{1}H NMR (400 MHz, $CDCl_3$) **BmP-F**

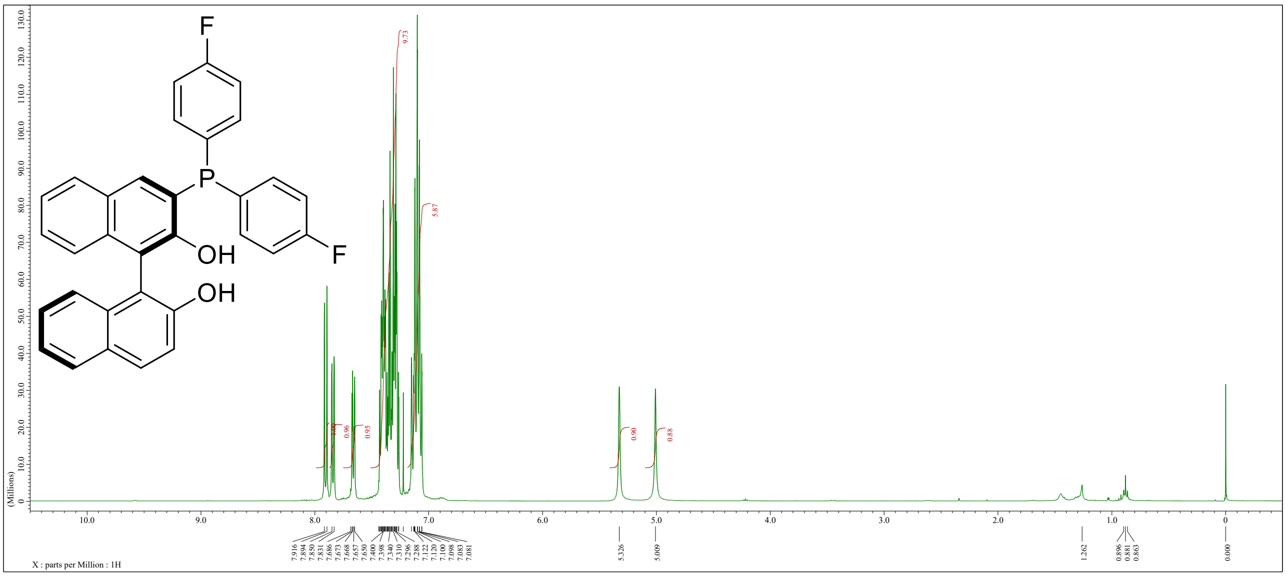

^{13}C{^{1}H} NMR (100 MHz, $CDCl_3$) **BmP-F**

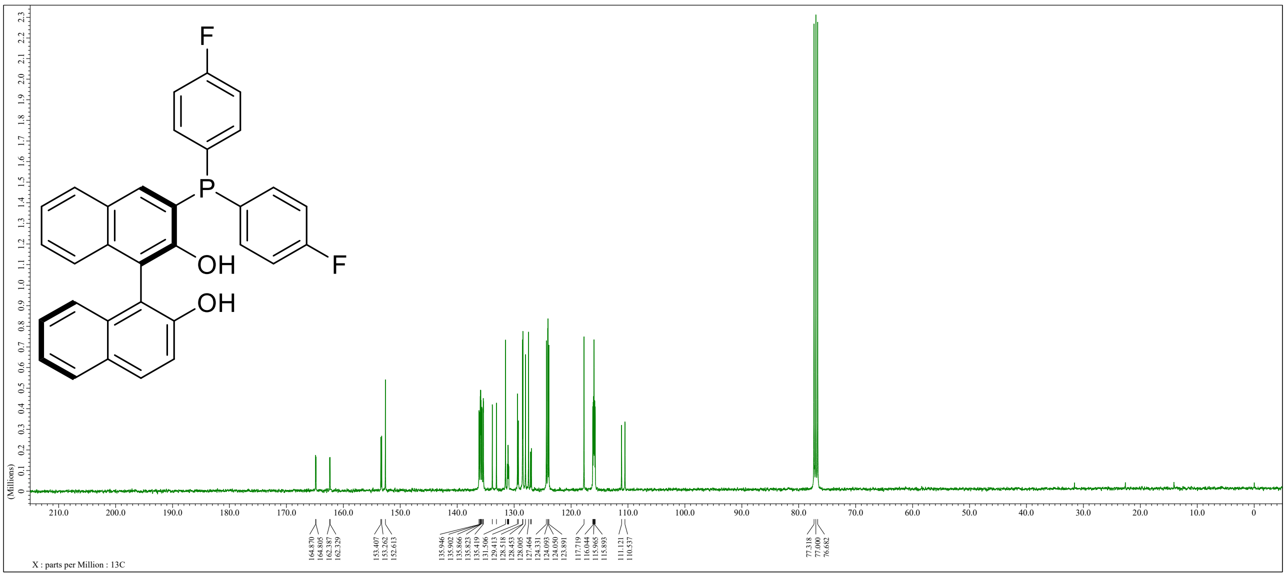

^{19}F NMR (375 MHz, $CDCl_3$) **BmP-F**

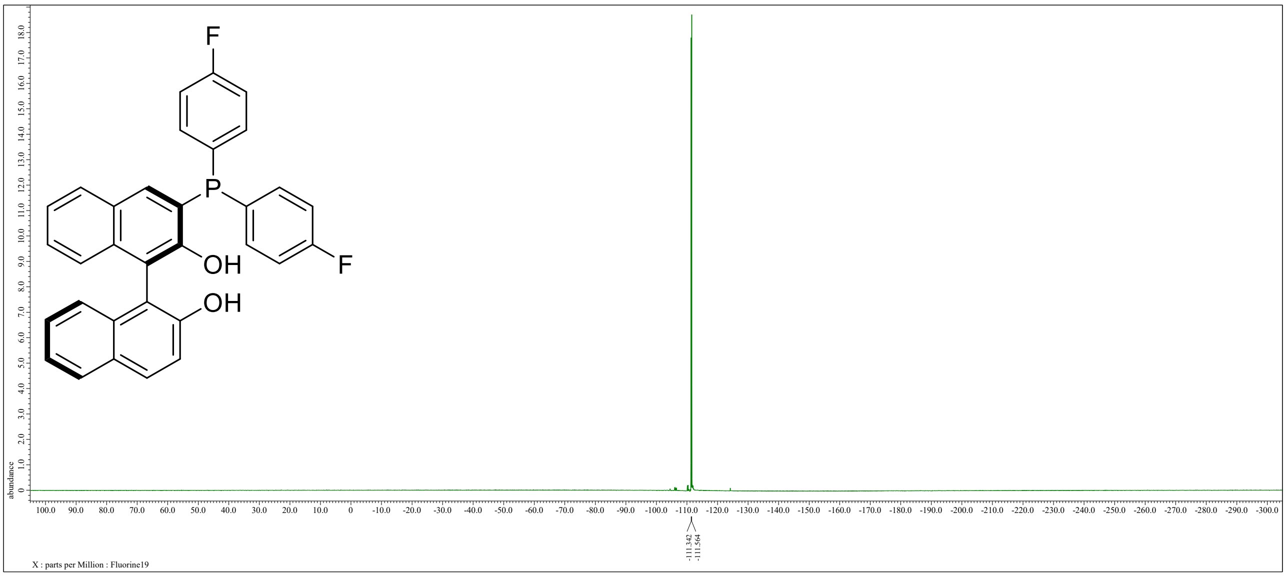

^{31}P NMR (162 MHz, $CDCl_3$) **BmP-F**

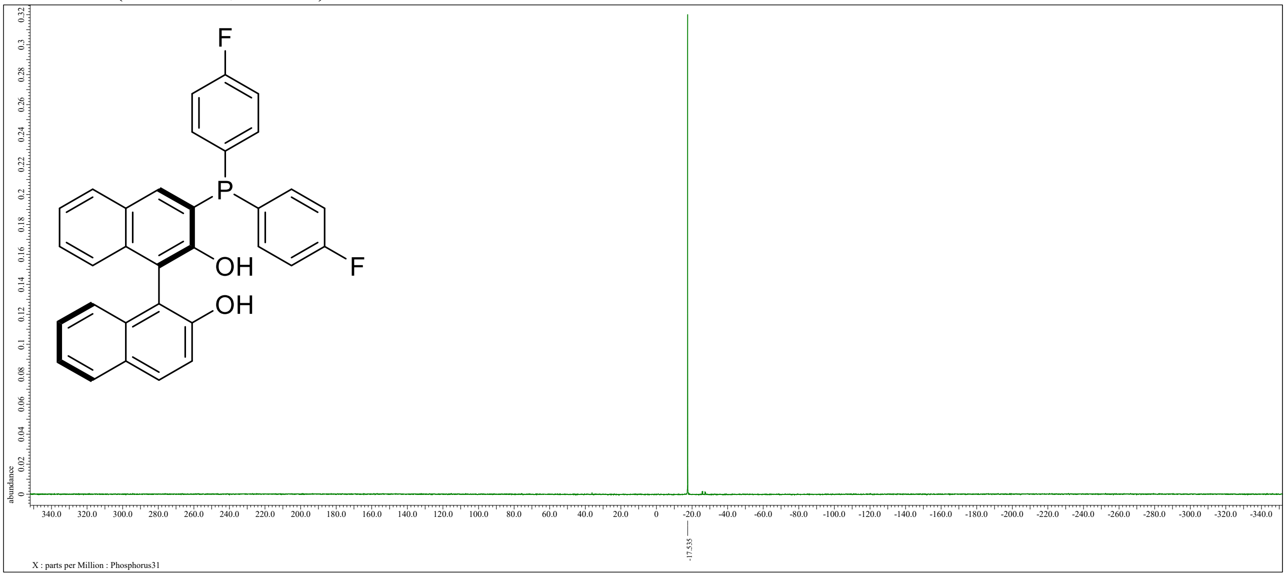

^{1}H NMR (400 MHz, $CDCl_3$) **BmP-3,5-^{i}Pr**

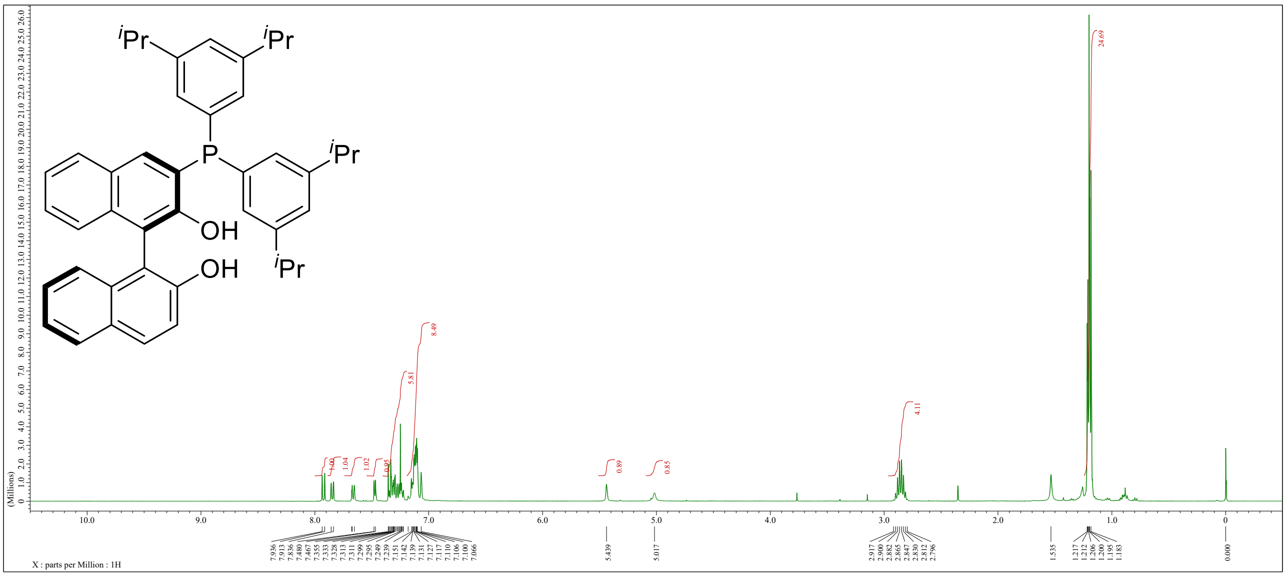

$^{13}C\{^{1}H\}$ NMR (100 MHz, $CDCl_3$) **BmP-3,5-^{i}Pr**

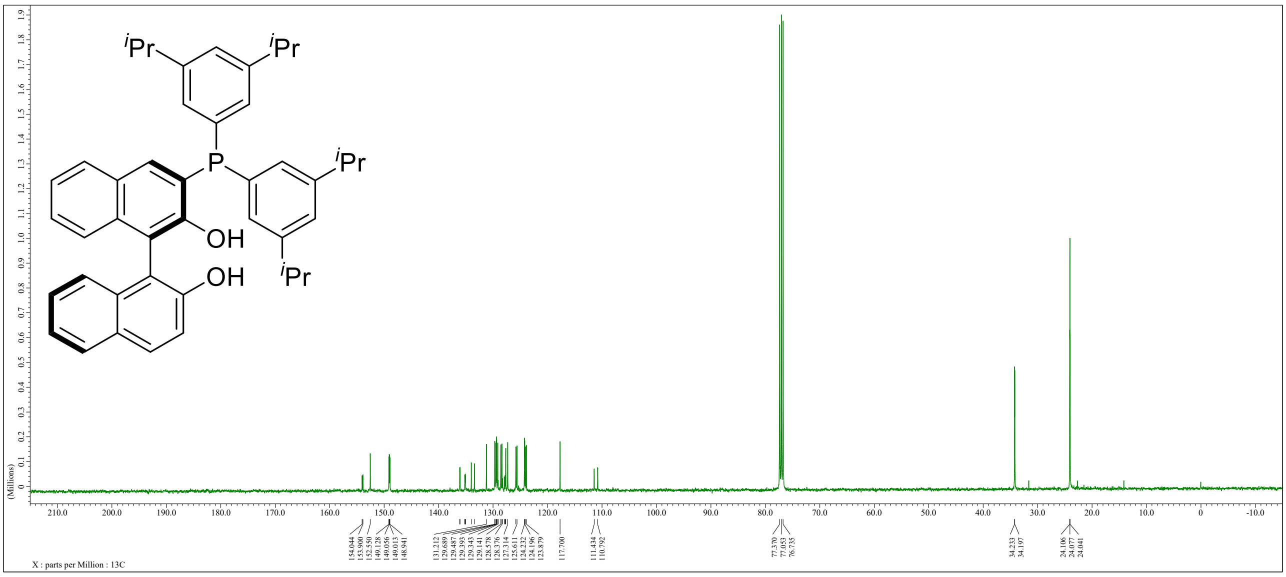

^{31}P NMR (162 MHz, $CDCl_3$) **BmP-3,5-^{i}Pr**

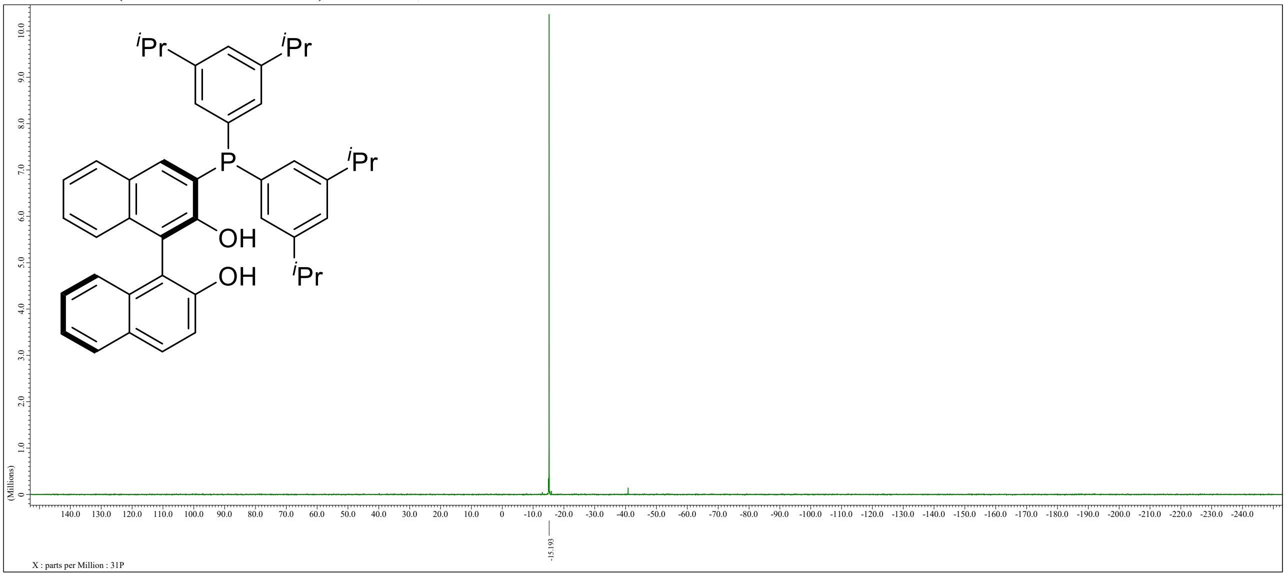

^{1}H NMR (400 MHz, $CDCl_3$) **S4**

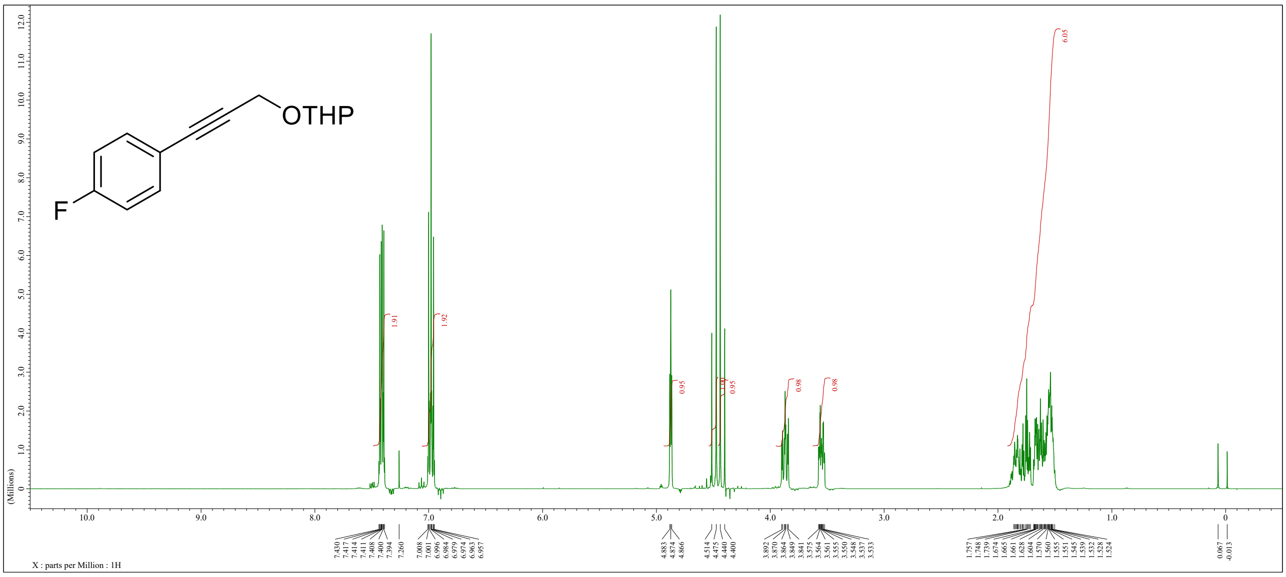

$^{13}C\{^{1}H\}$ NMR (100 MHz, $CDCl_3$) **S4**

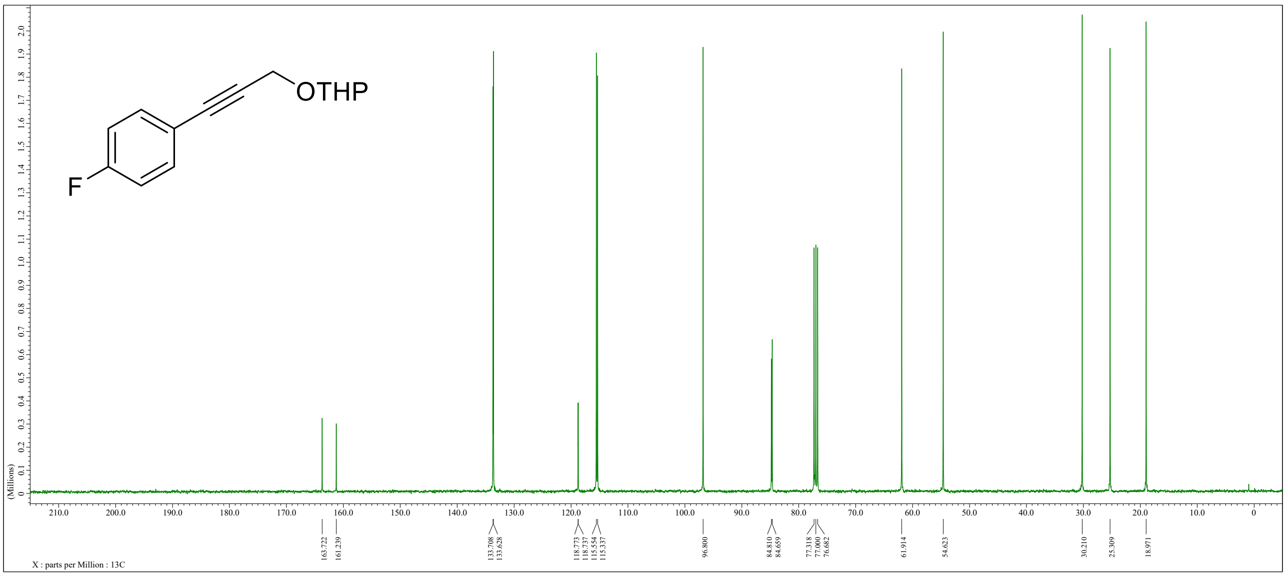

^{19}F NMR (375 MHz, $CDCl_3$) **S4**

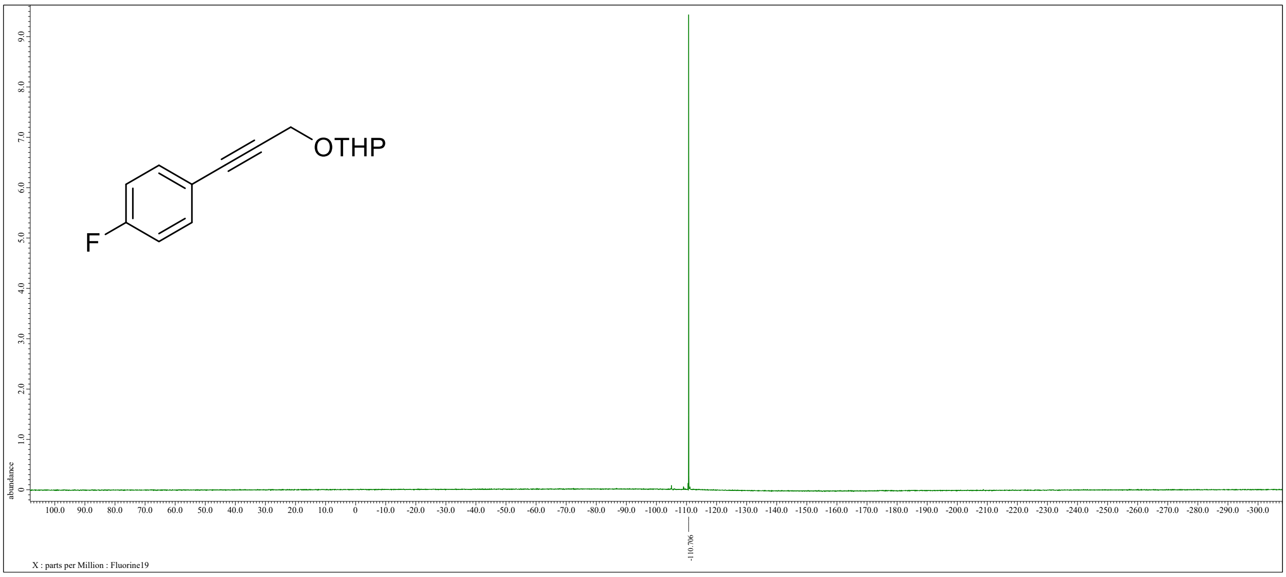

^{1}H NMR (400 MHz, $CDCl_3$) **S5**

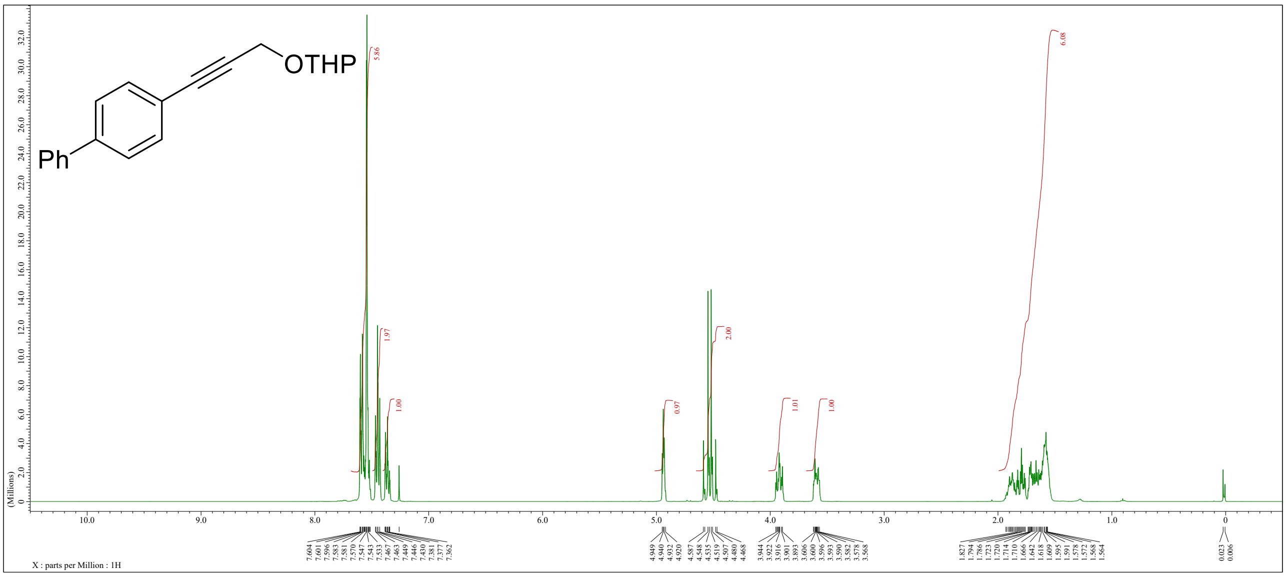

$^{13}C\{^{1}H\}$ NMR (100 MHz, $CDCl_3$) **S5**

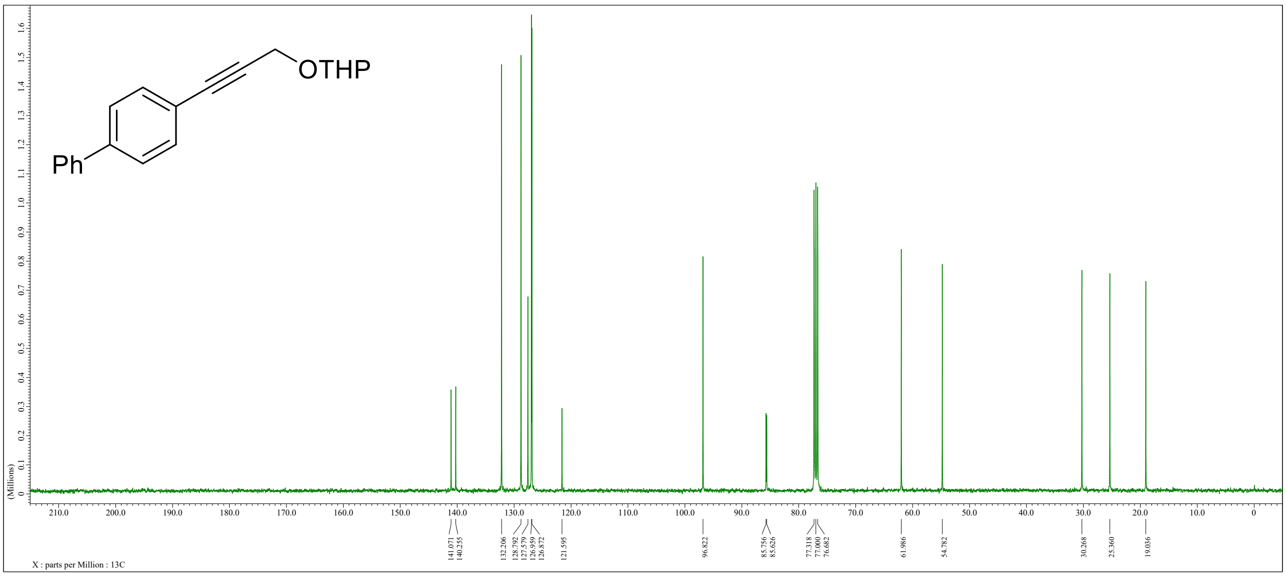

^{1}H NMR (400 MHz, $CDCl_3$) **S7**

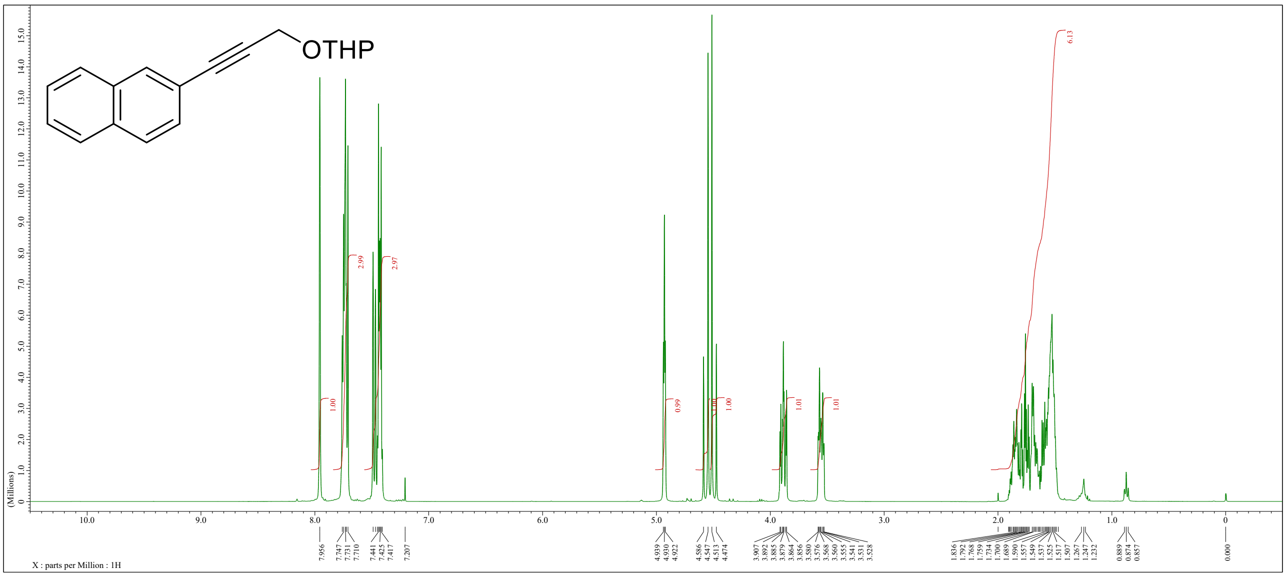

$^{13}C\{^{1}H\}$ NMR (100 MHz, $CDCl_3$) **S7**

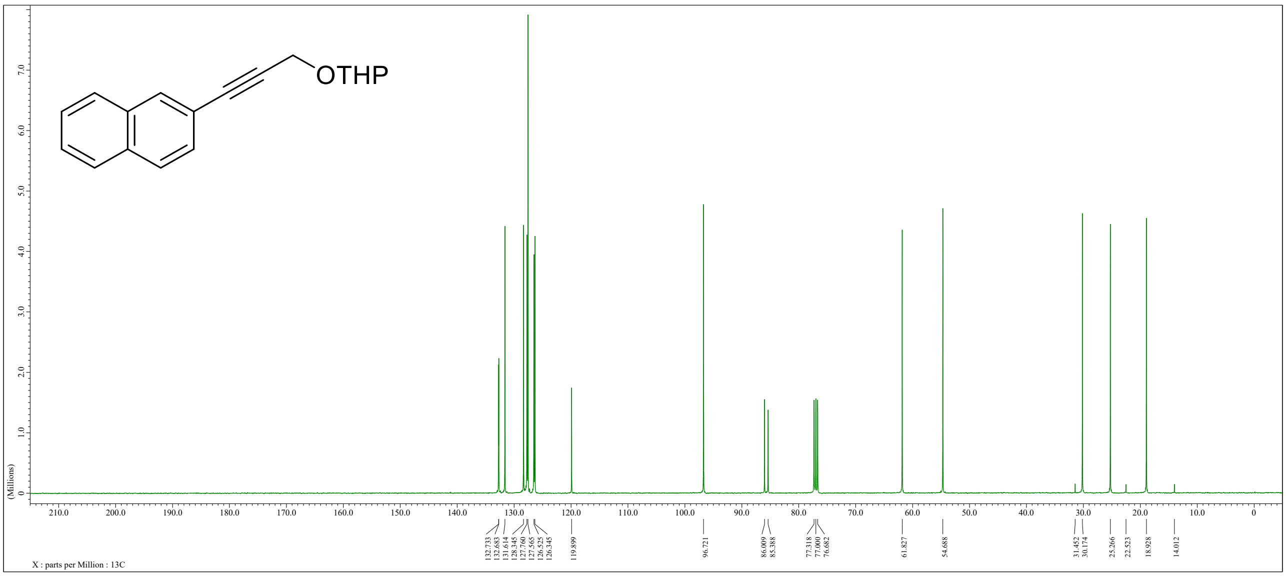

^{1}H NMR (400 MHz, $CDCl_3$) **S9**

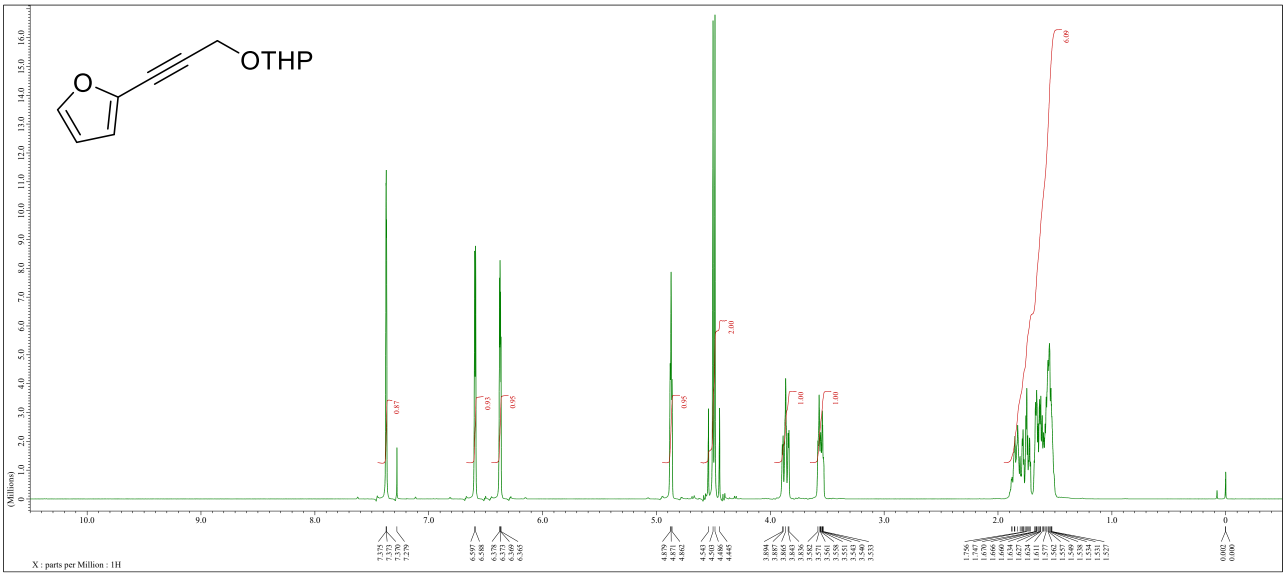

$^{13}C\{^{1}H\}$ NMR (100 MHz, $CDCl_3$) **S9**

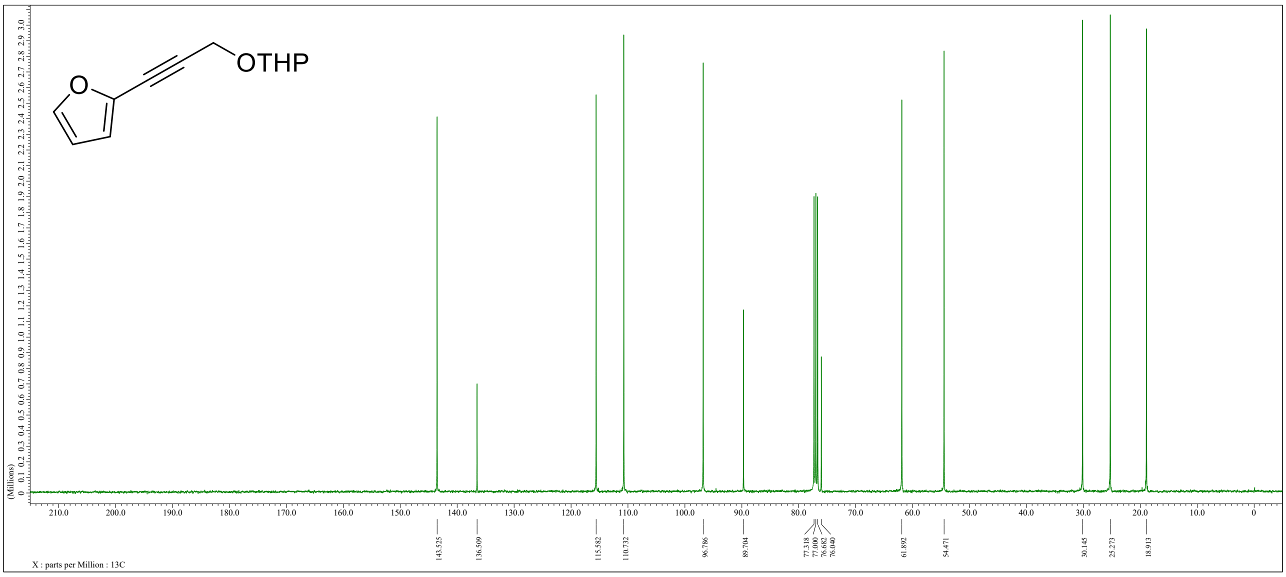

^{1}H NMR (400 MHz, $CDCl_3$) **S11**

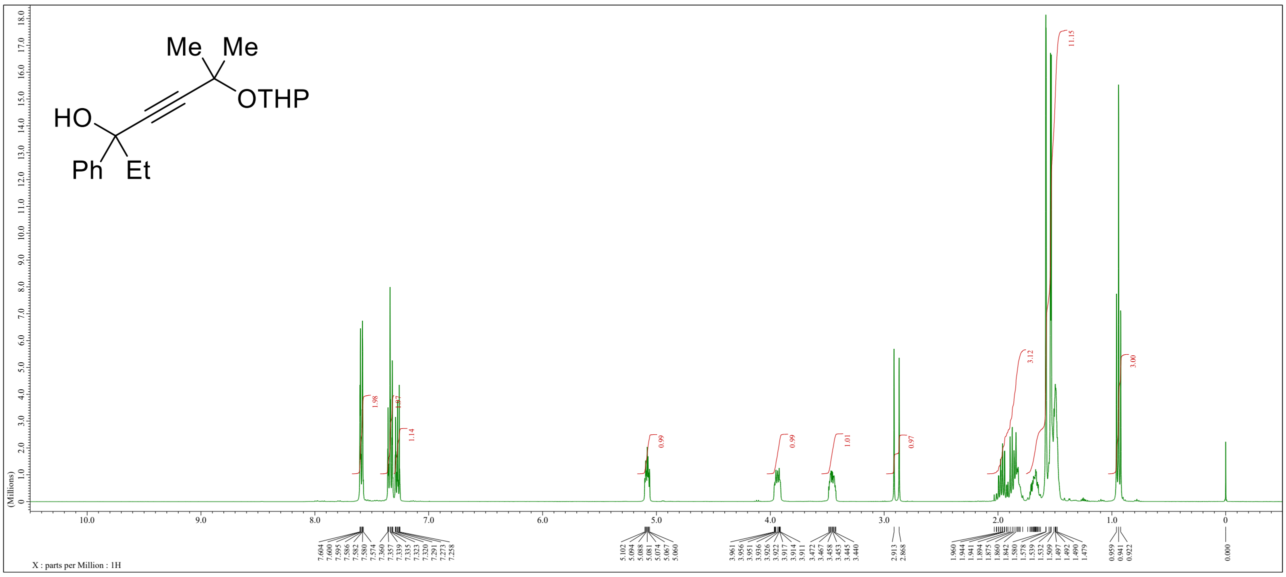

$^{13}C\{^{1}H\}$ NMR (100 MHz, $CDCl_3$) **S11**

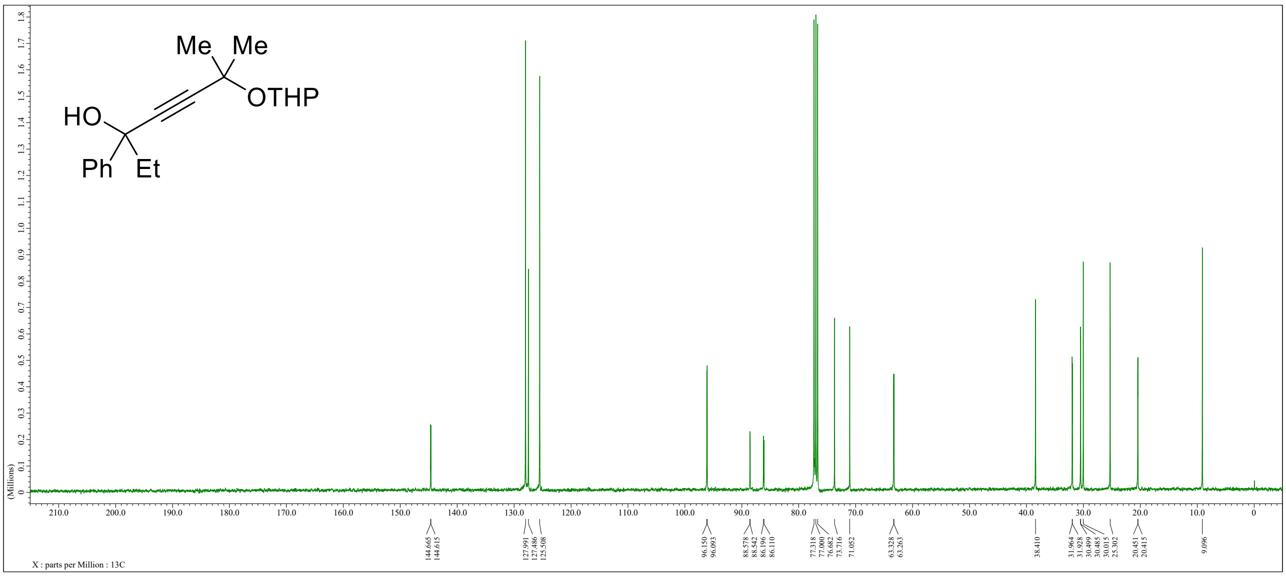

^{1}H NMR (400 MHz, $CDCl_3$) **S12**

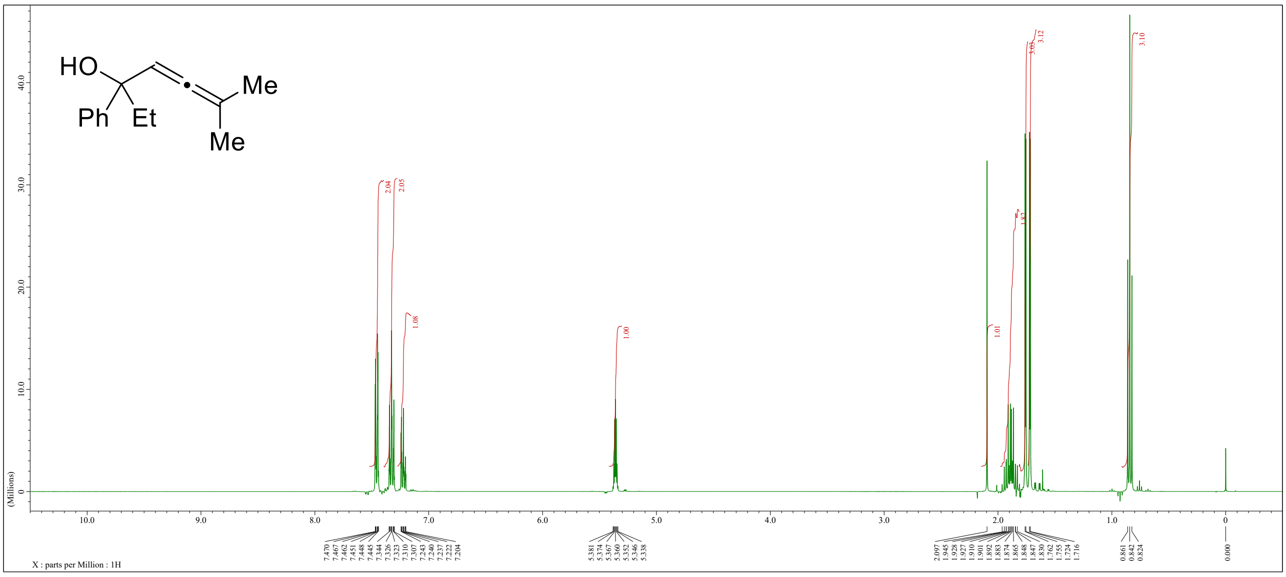

$^{13}C\{^{1}H\}$ NMR (100 MHz, $CDCl_3$) **S12**

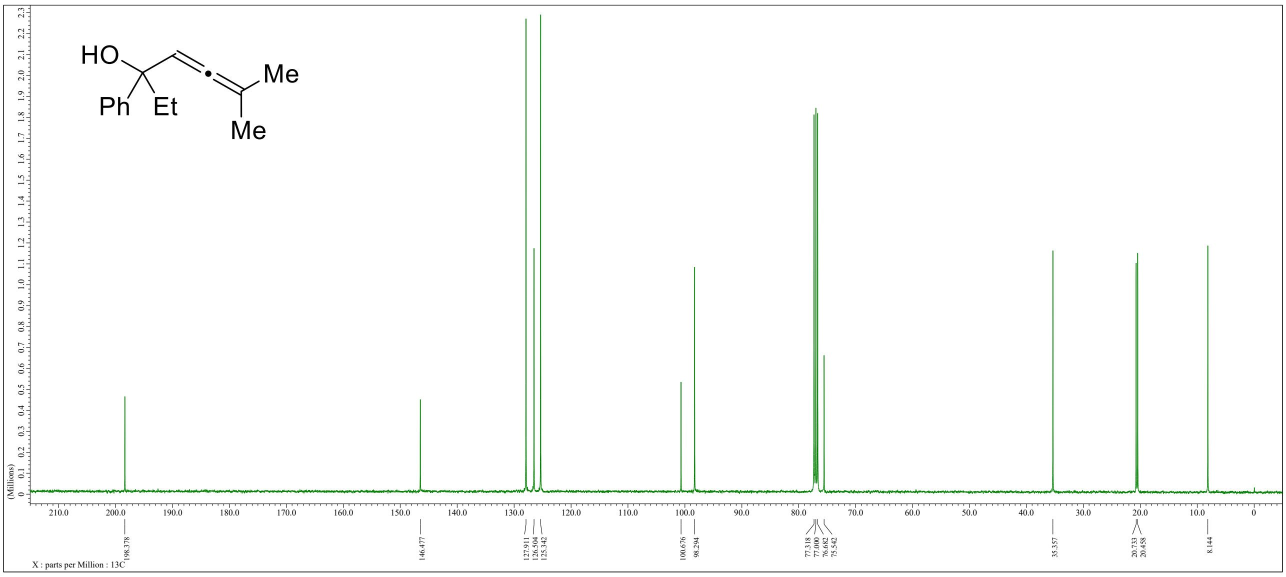

^{1}H NMR (400 MHz, $CDCl_3$) **S13**

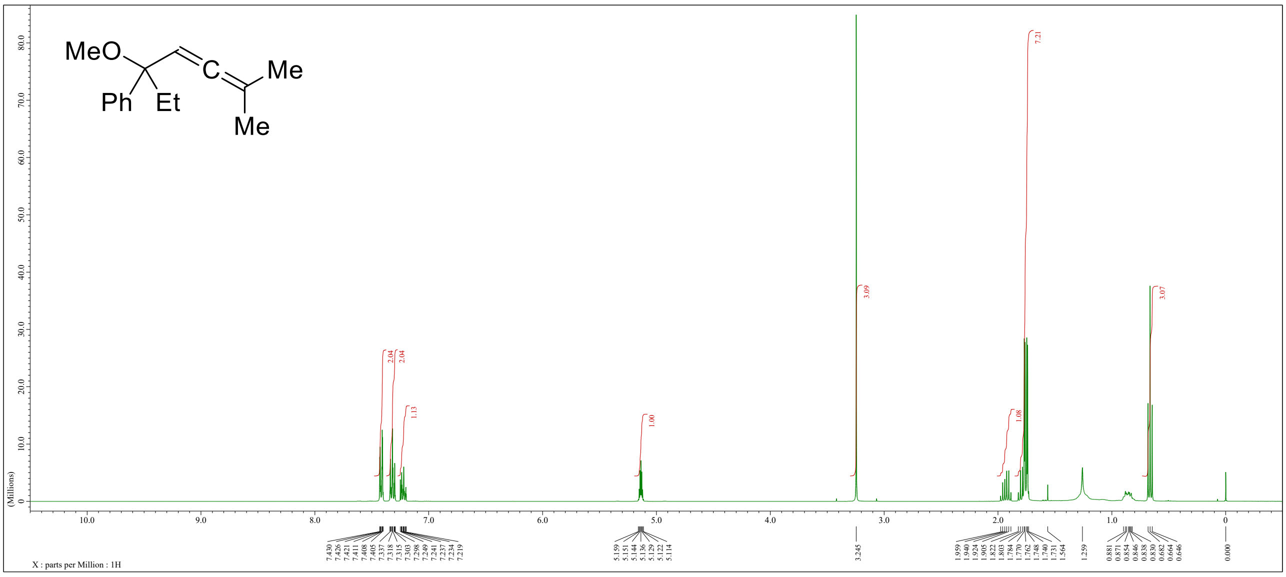

^{13}C{^{1}H} NMR (100 MHz, $CDCl_3$) **S13**

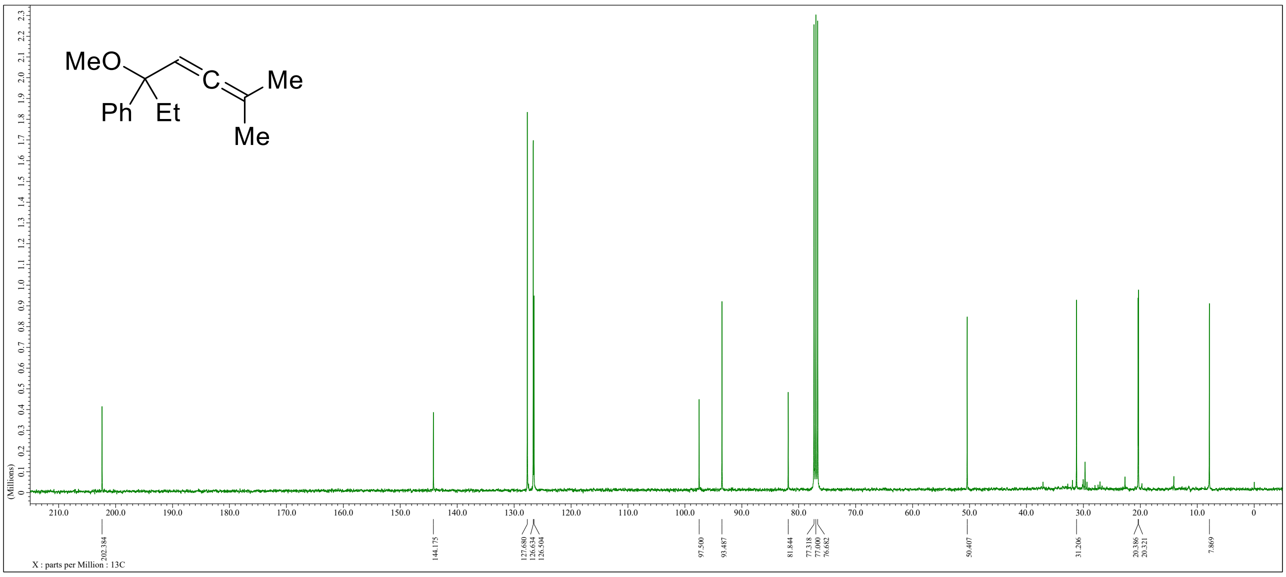

^{1}H NMR (400 MHz, $CDCl_3$) (*Z*)-**1a**

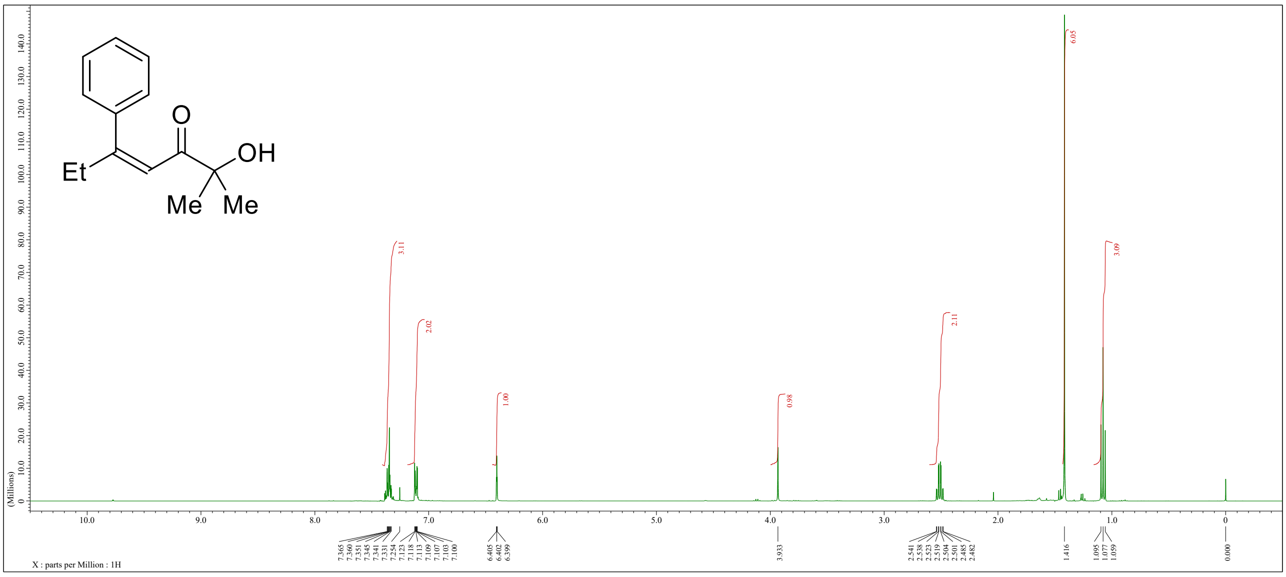

^{13}C{^{1}H} NMR (100 MHz, $CDCl_3$) (*Z*)-**1a**

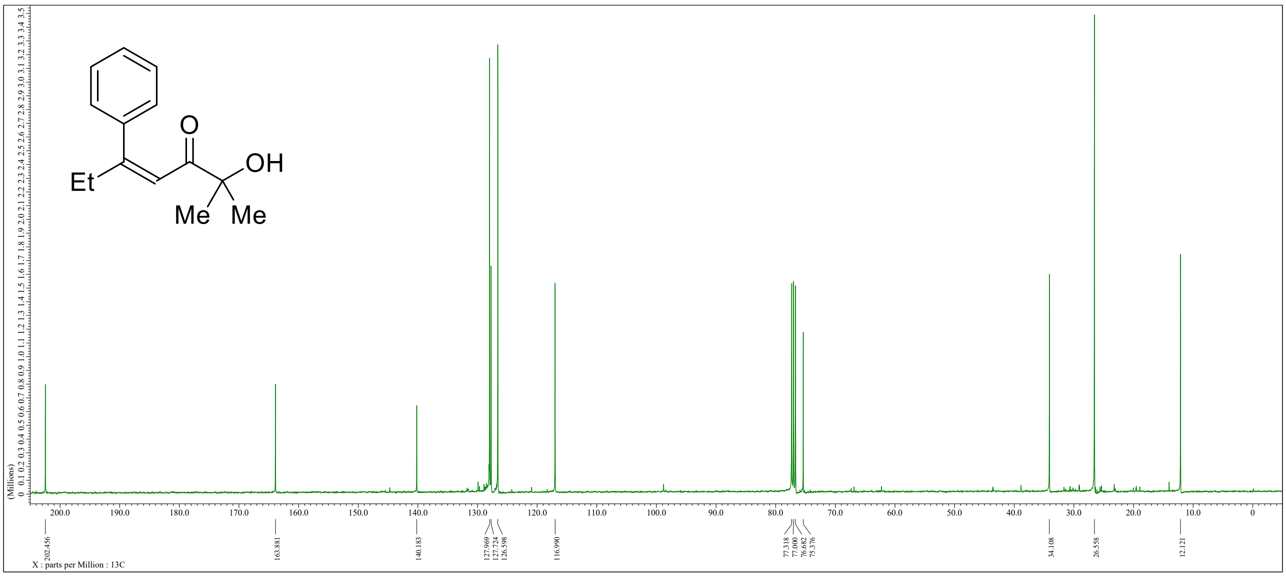

^{1}H NMR (400 MHz, $CDCl_3$) (*E*)-**1a**

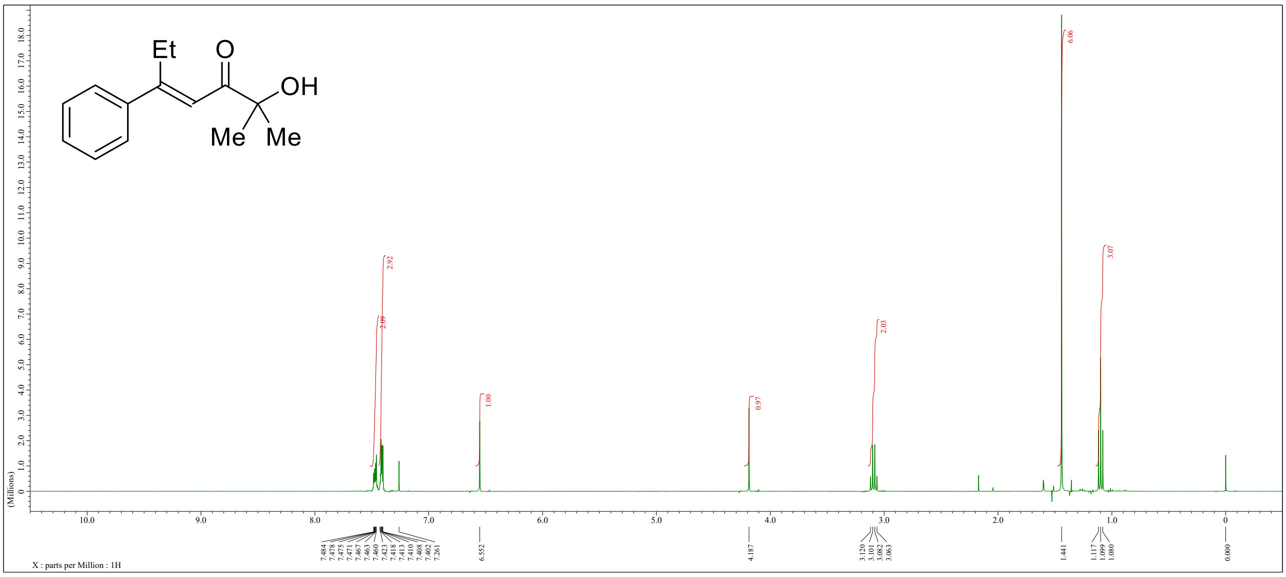

^{13}C{^{1}H} NMR (100 MHz, $CDCl_3$) (*E*)-**1a**

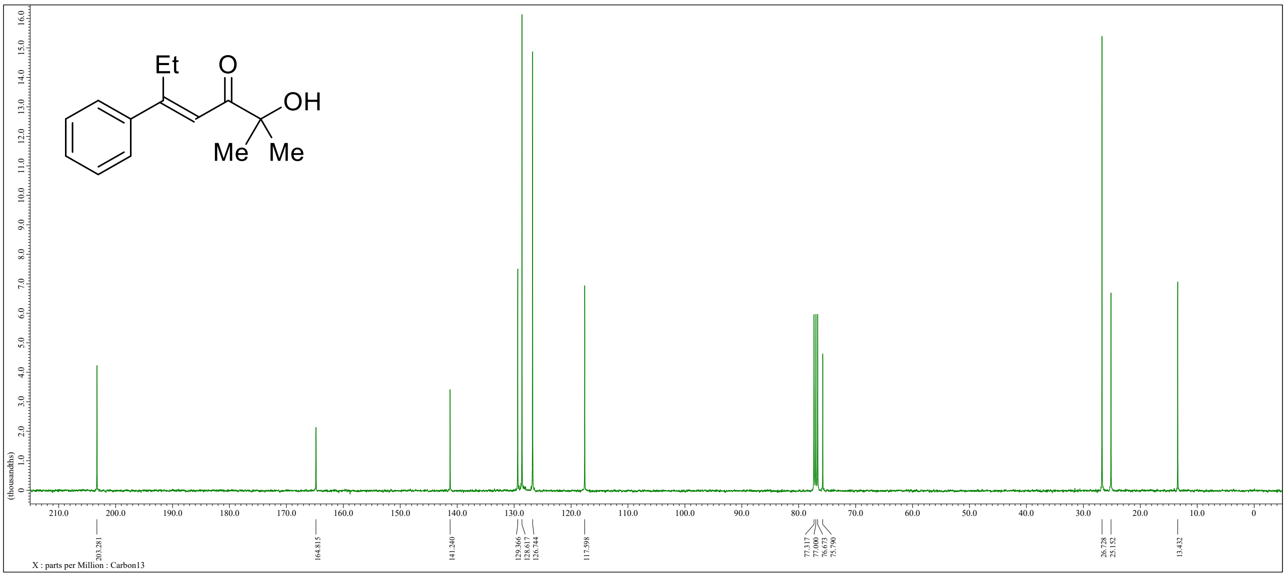

^{1}H NMR (400 MHz, $CDCl_3$) (*E*)-**1b**

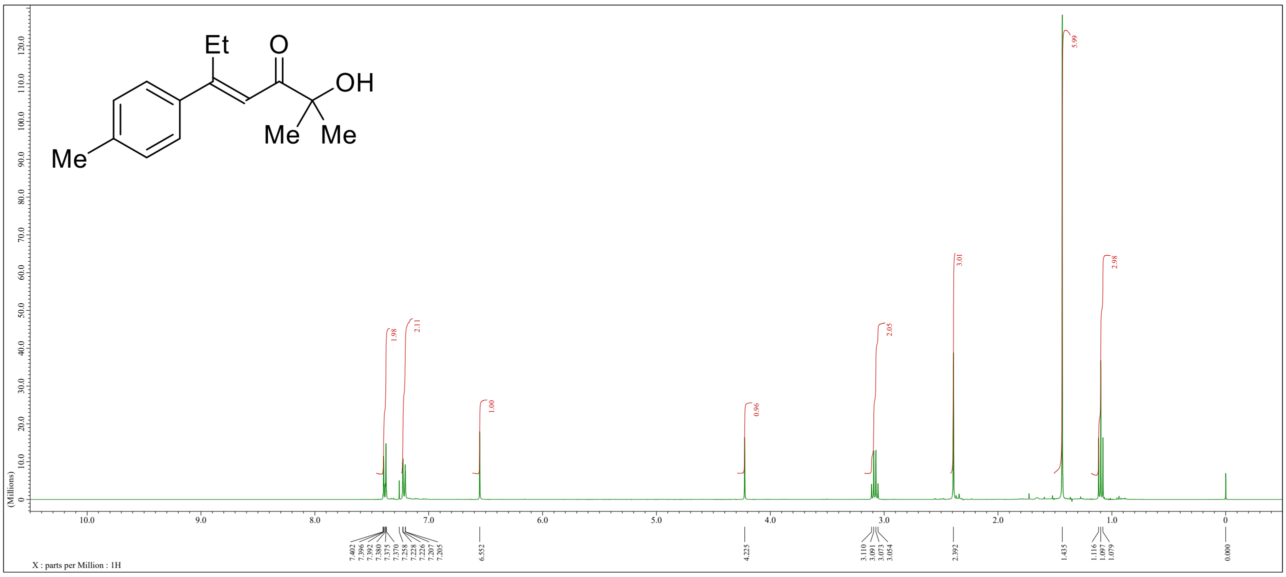

^{13}C{^{1}H} NMR (100 MHz, $CDCl_3$) (*E*)-**1b**

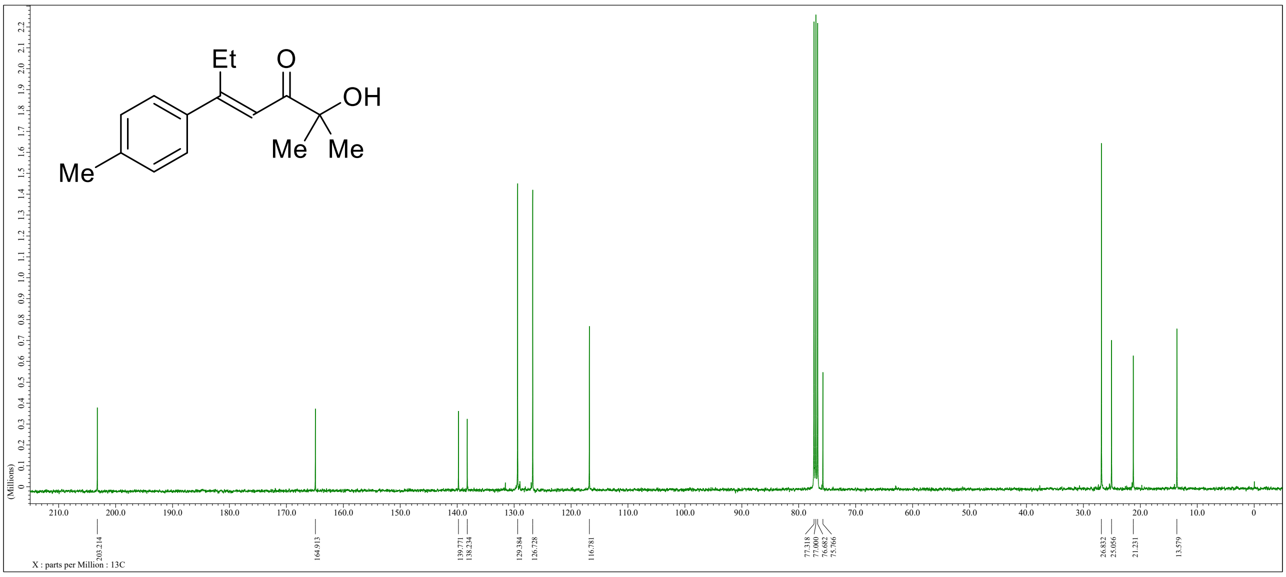

^{1}H NMR (400 MHz, $CDCl_3$) (*E*)-**1c**

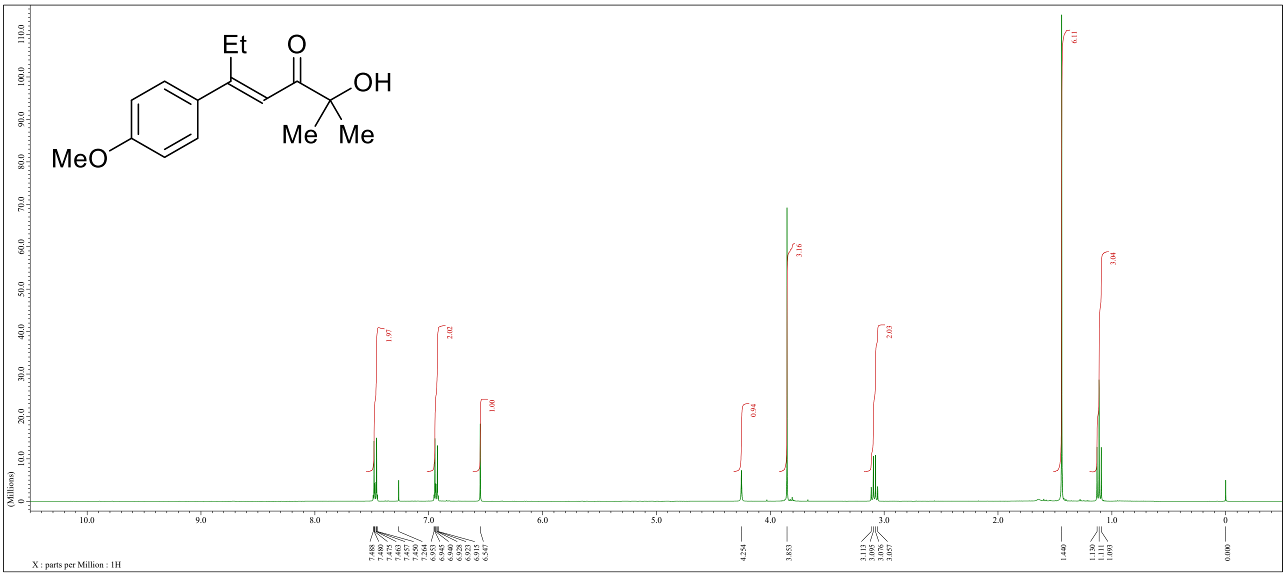

$^{13}C\{^{1}H\}$ NMR (100 MHz, $CDCl_3$) (*E*)-**1c**

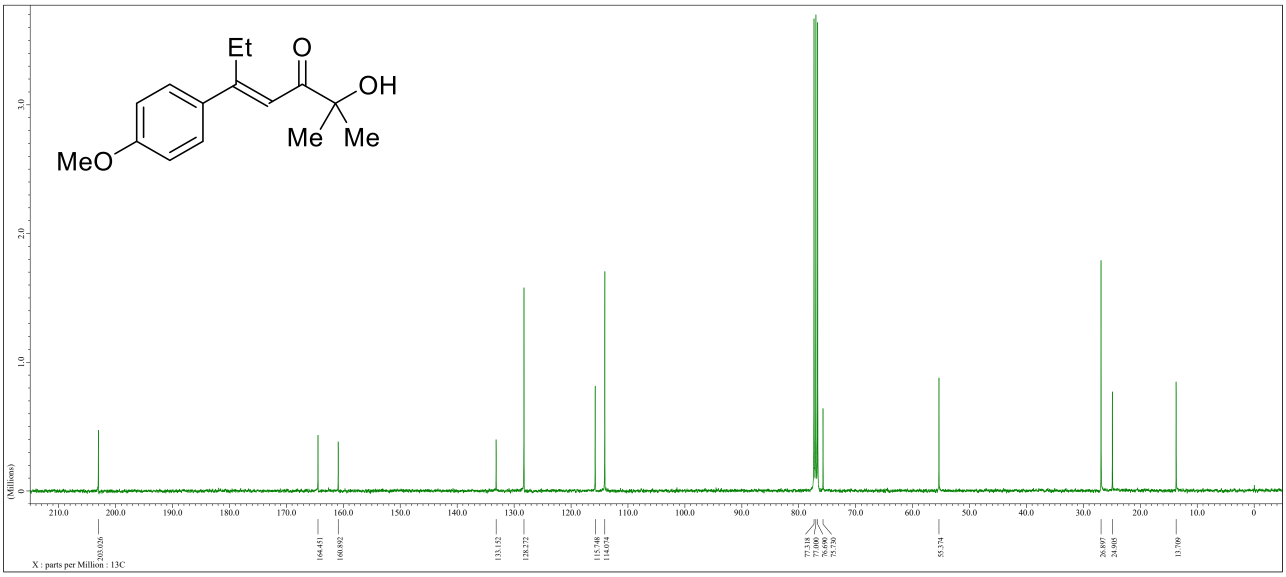

^{1}H NMR (400 MHz, $CDCl_3$) (*E*)-**1d**

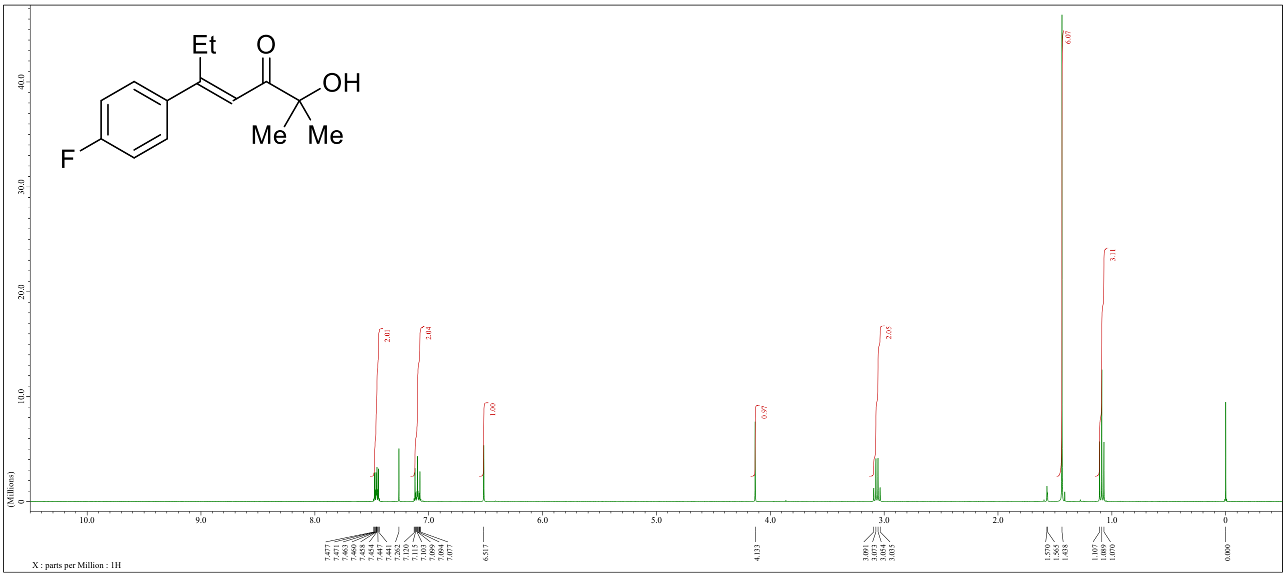

$^{13}C\{^{1}H\}$ NMR (100 MHz, $CDCl_3$) (*E*)-**1d**

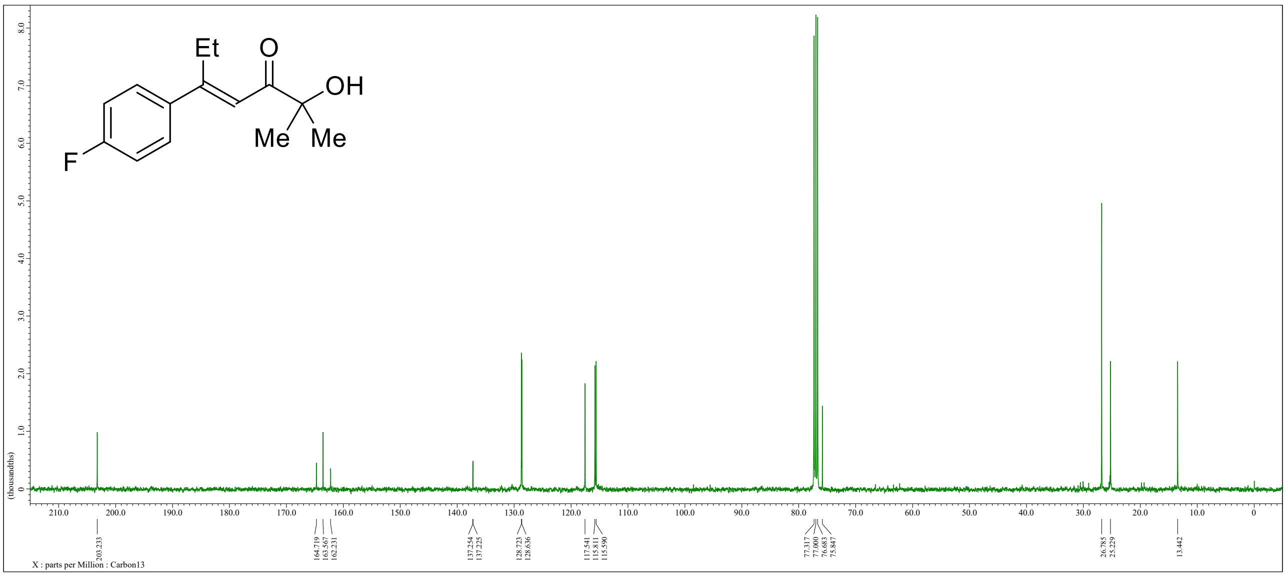

^{19}F NMR (375 MHz, $CDCl_3$) (*E*)-**1d**

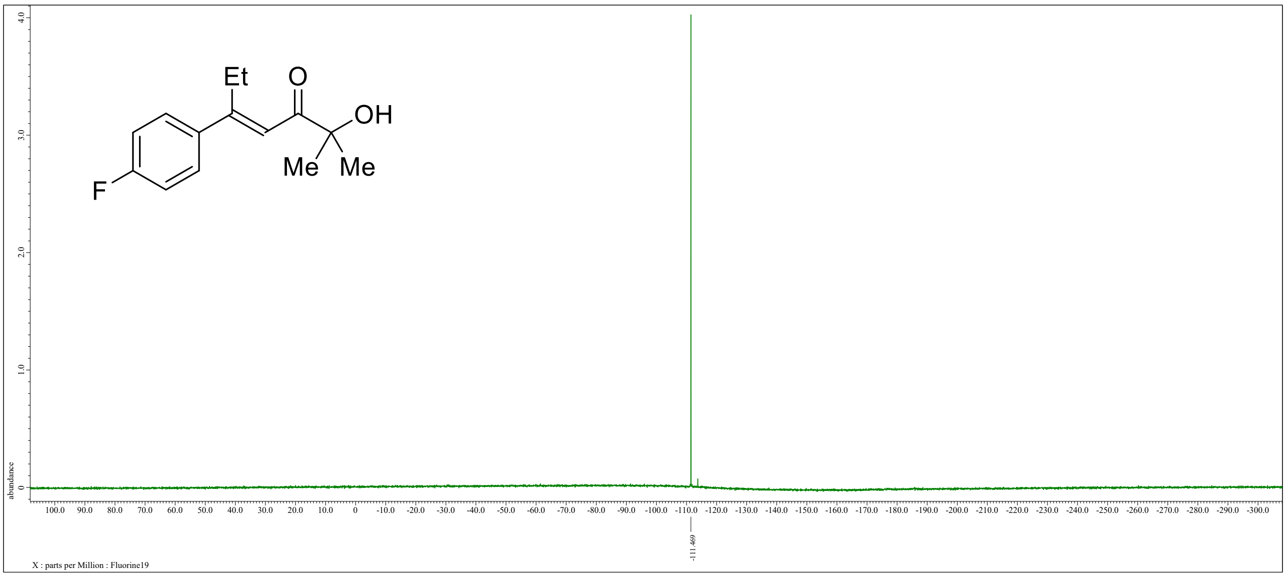

^{1}H NMR (400 MHz, CDCl$_3$) (*E*)-**1e**

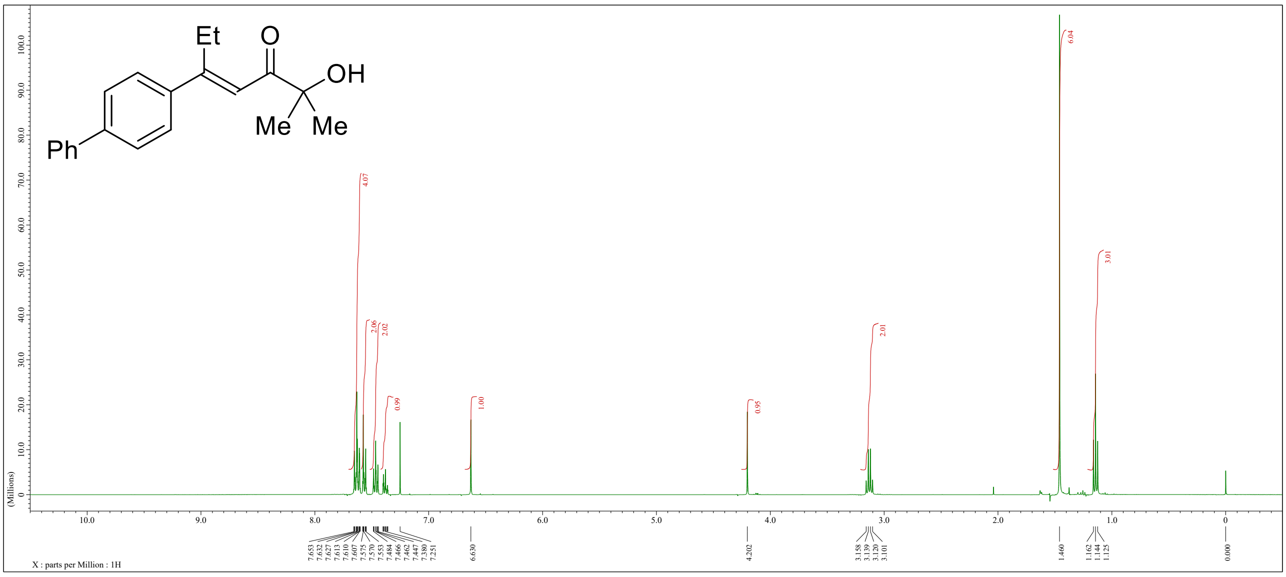

^{13}C{^{1}H} NMR (100 MHz, CDCl$_3$) (*E*)-**1e**

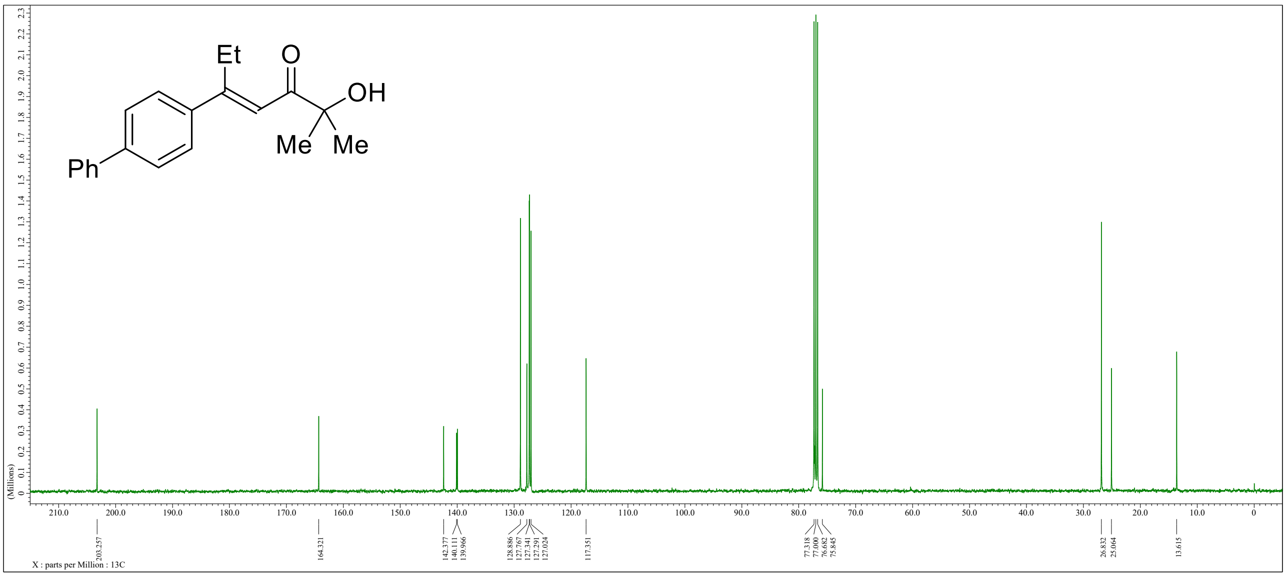

^{1}H NMR (400 MHz, $CDCl_3$) (*E*)-**1f**

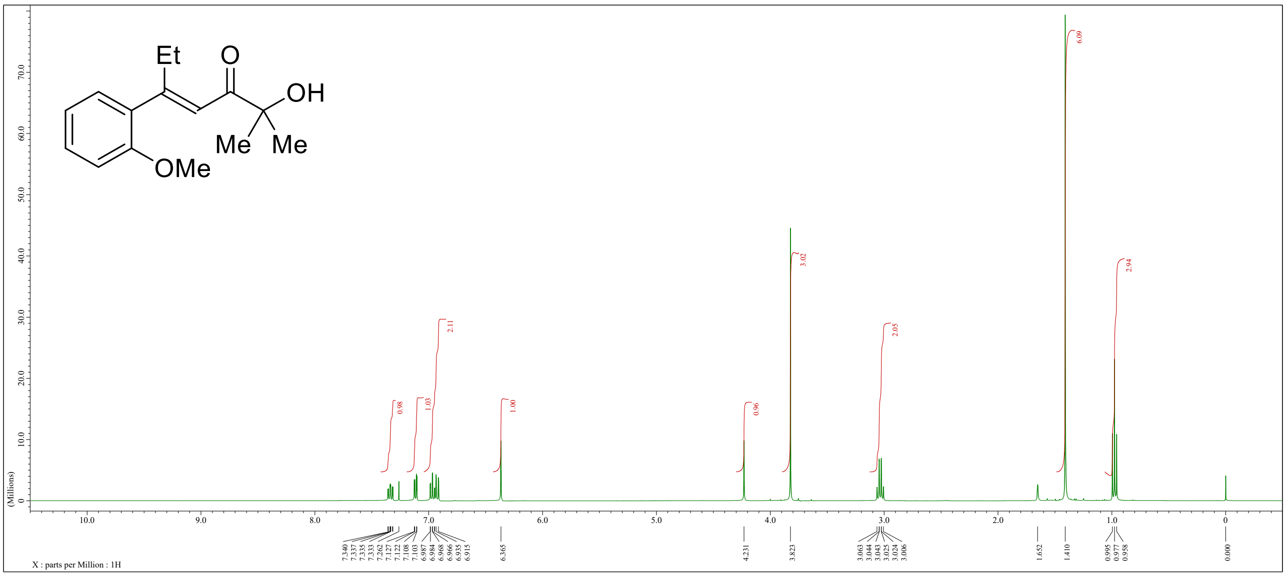

$^{13}C\{^{1}H\}$ NMR (100 MHz, $CDCl_3$) (*E*)-**1f**

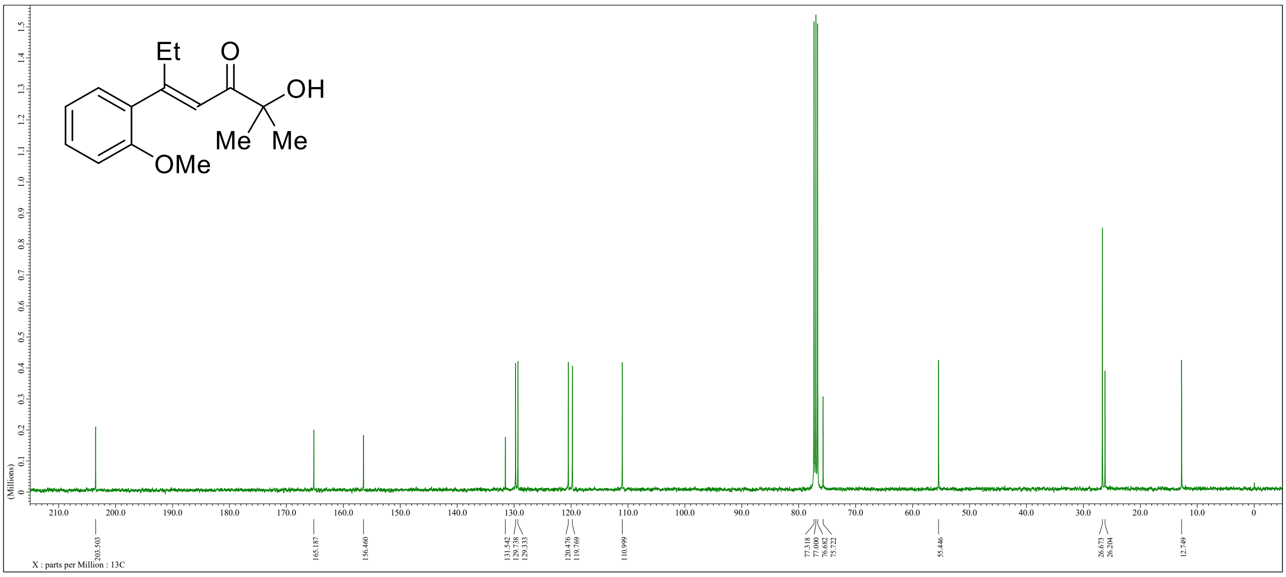

^{1}H NMR (400 MHz, $CDCl_3$) (*E*)-**1g**

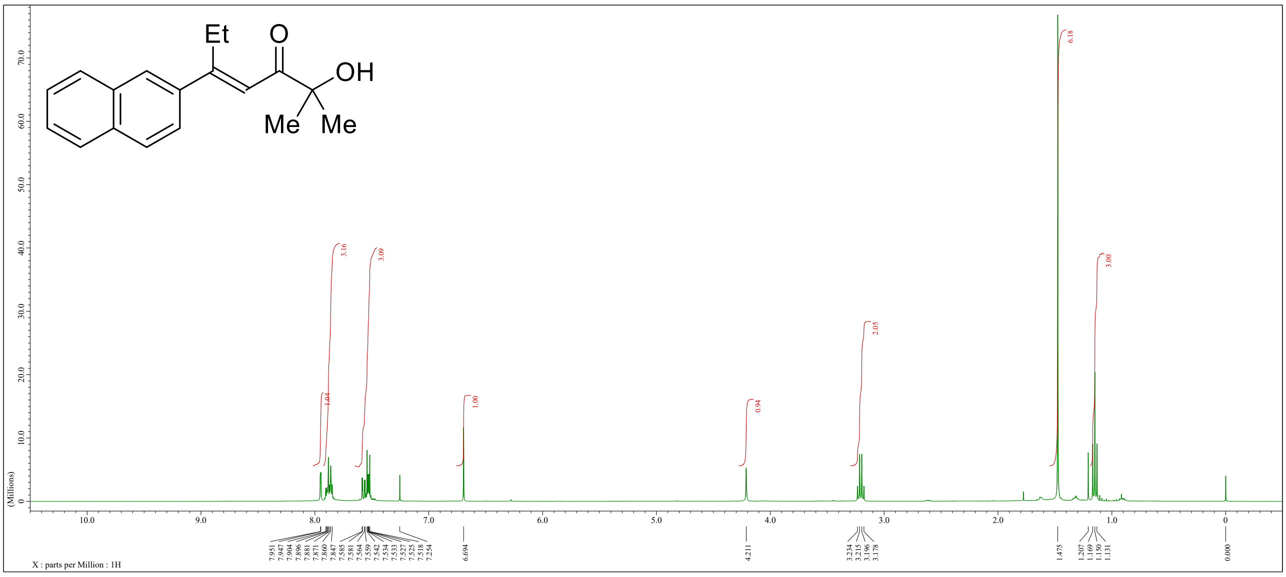

^{13}C{^{1}H} NMR (100 MHz, $CDCl_3$) (*E*)-**1g**

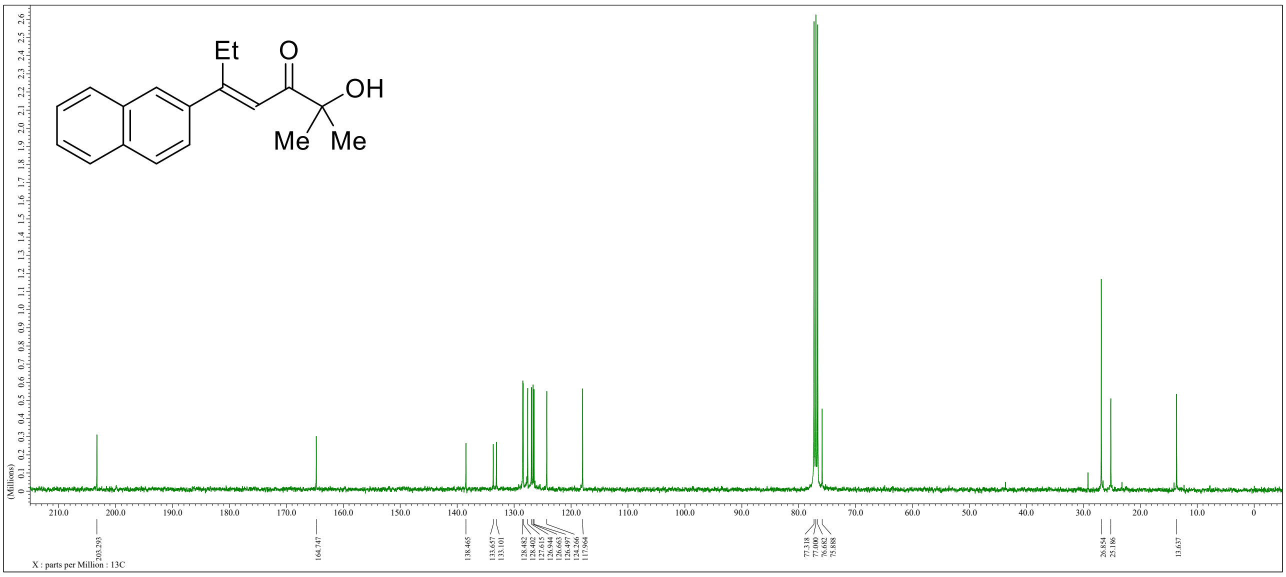

^{1}H NMR (400 MHz, $CDCl_3$) (*E*)-**1h**

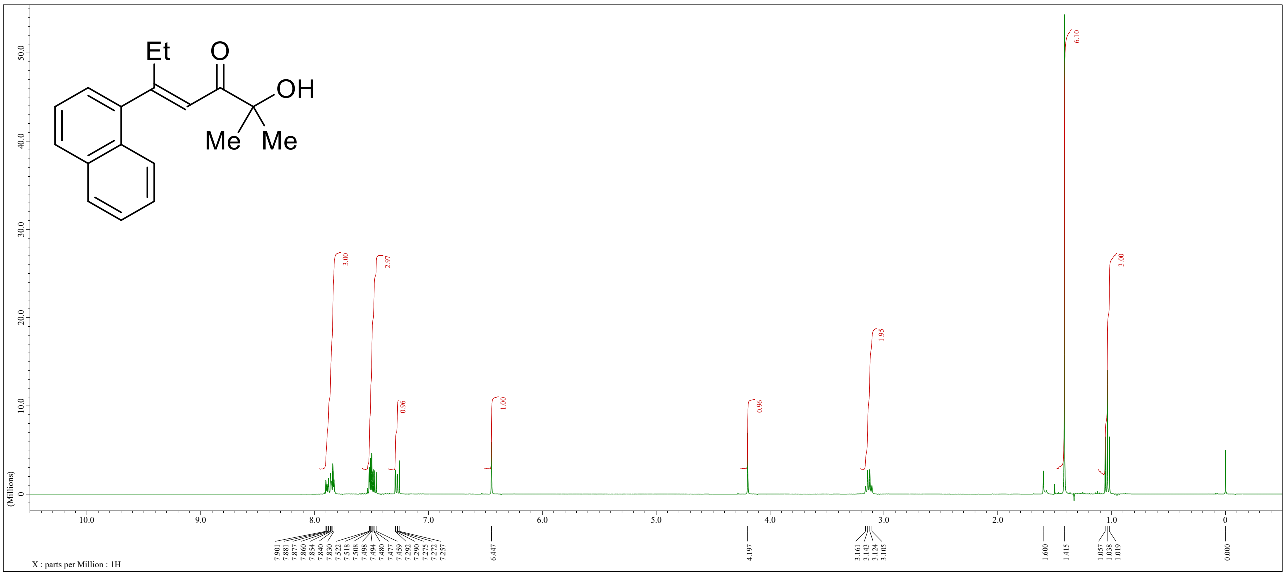

$^{13}C\{^{1}H\}$ NMR (100 MHz, $CDCl_3$) (*E*)-**1h**

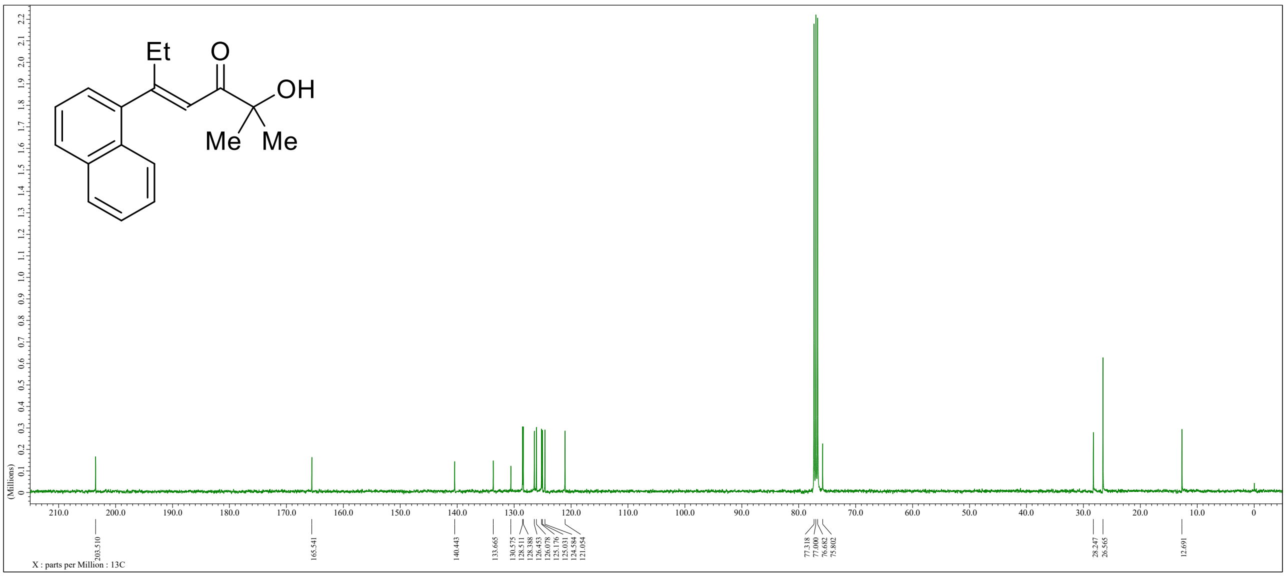

^{1}H NMR (400 MHz, $CDCl_3$) (*E*)-**1i**

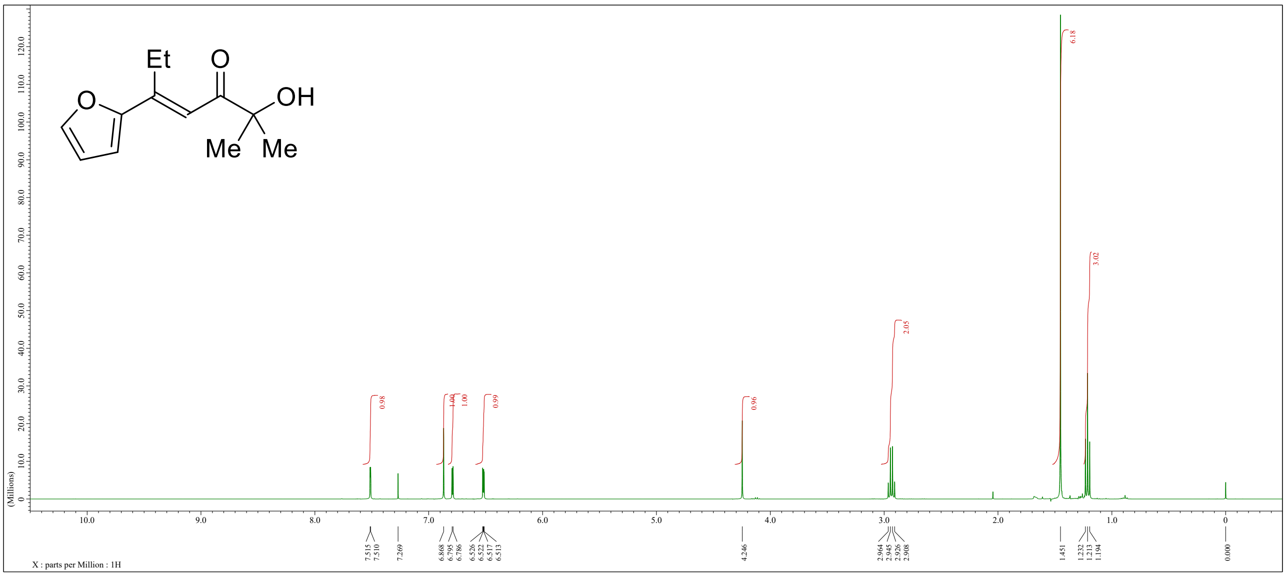

^{13}C{^{1}H} NMR (100 MHz, $CDCl_3$) (*E*)-**1i**

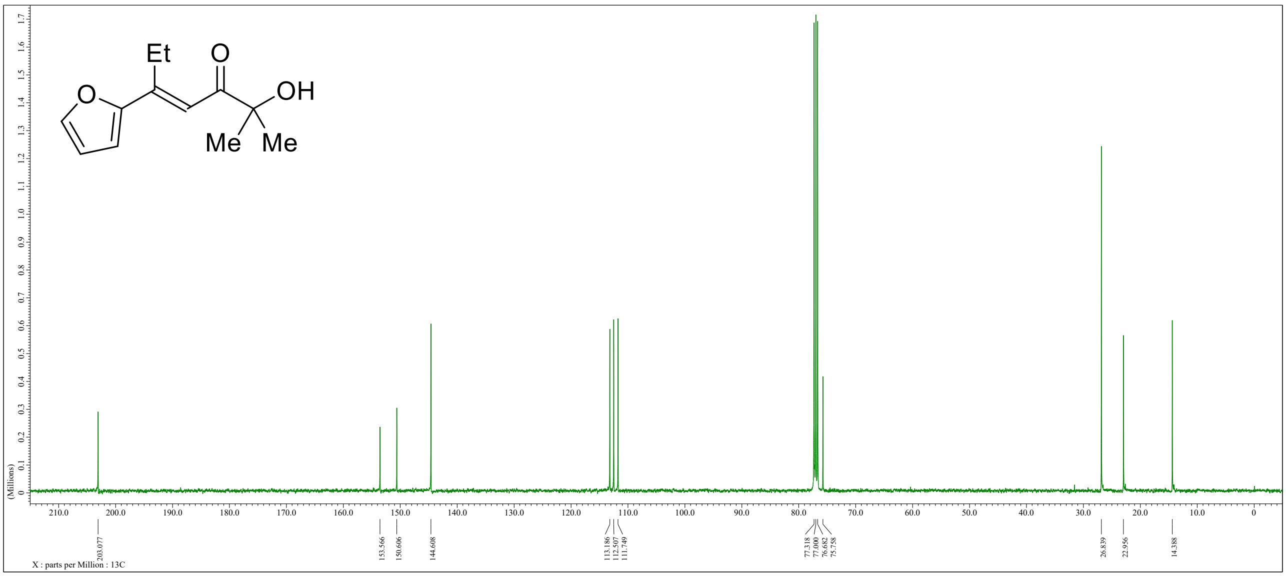

^{1}H NMR (400 MHz, $CDCl_3$) (*E*)-**1j**

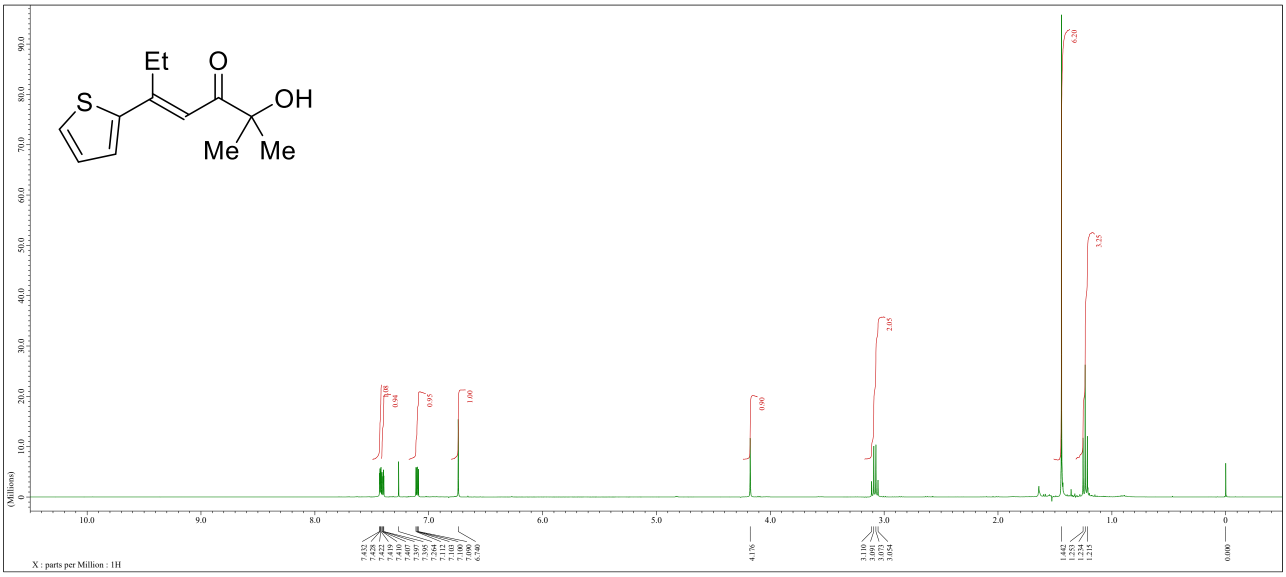

$^{13}C\{^{1}H\}$ NMR (100 MHz, $CDCl_3$) (*E*)-**1j**

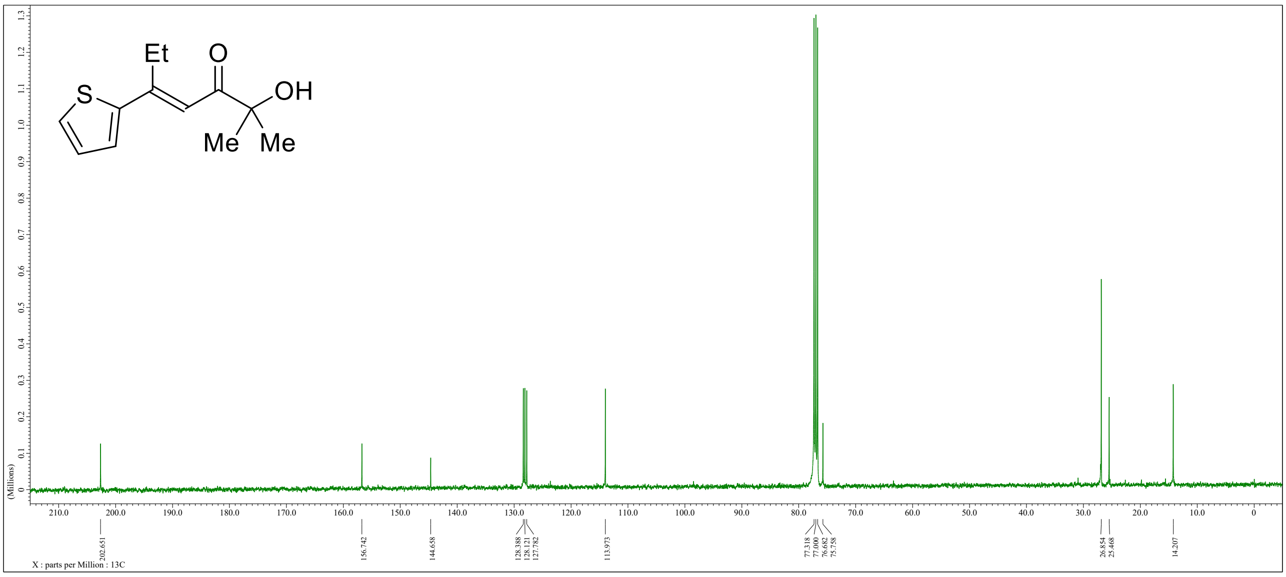

^{1}H NMR (400 MHz, $CDCl_3$) (*E*)-**1k**

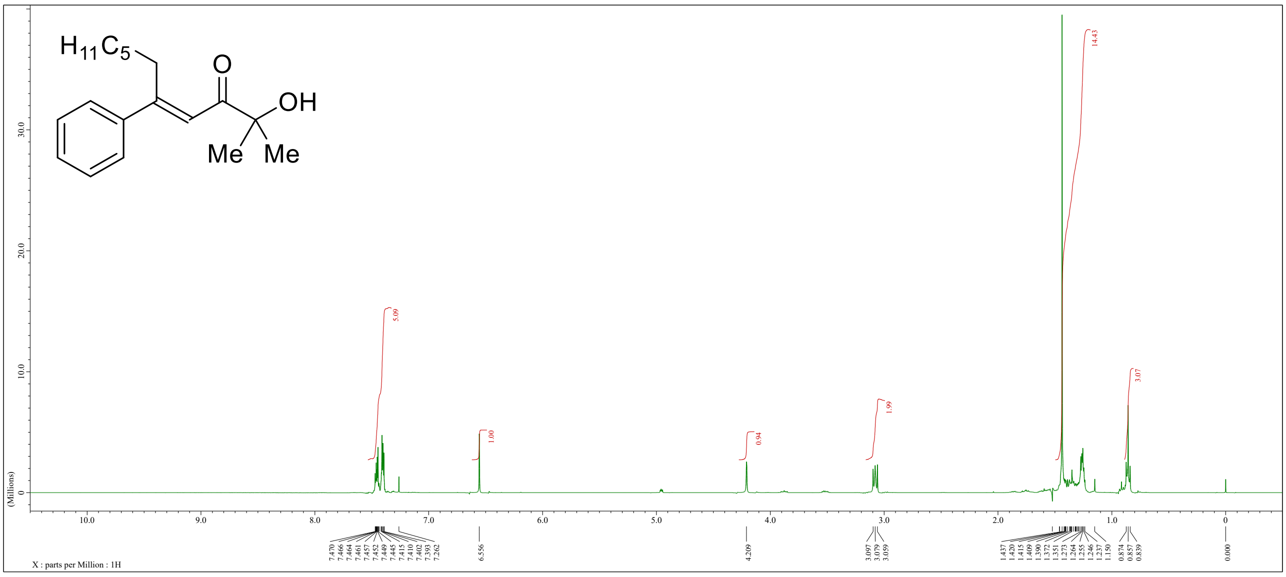

^{13}C{^{1}H} NMR (100 MHz, $CDCl_3$) (*E*)-**1k**

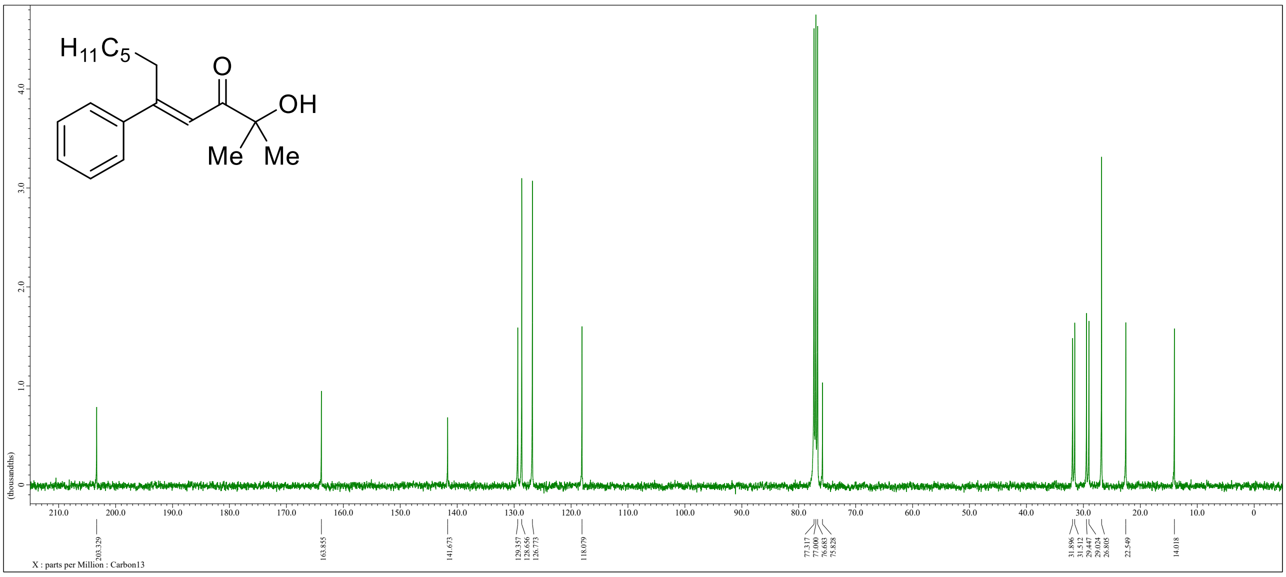

^{1}H NMR (400 MHz, $CDCl_3$) (*E*)-**1l**

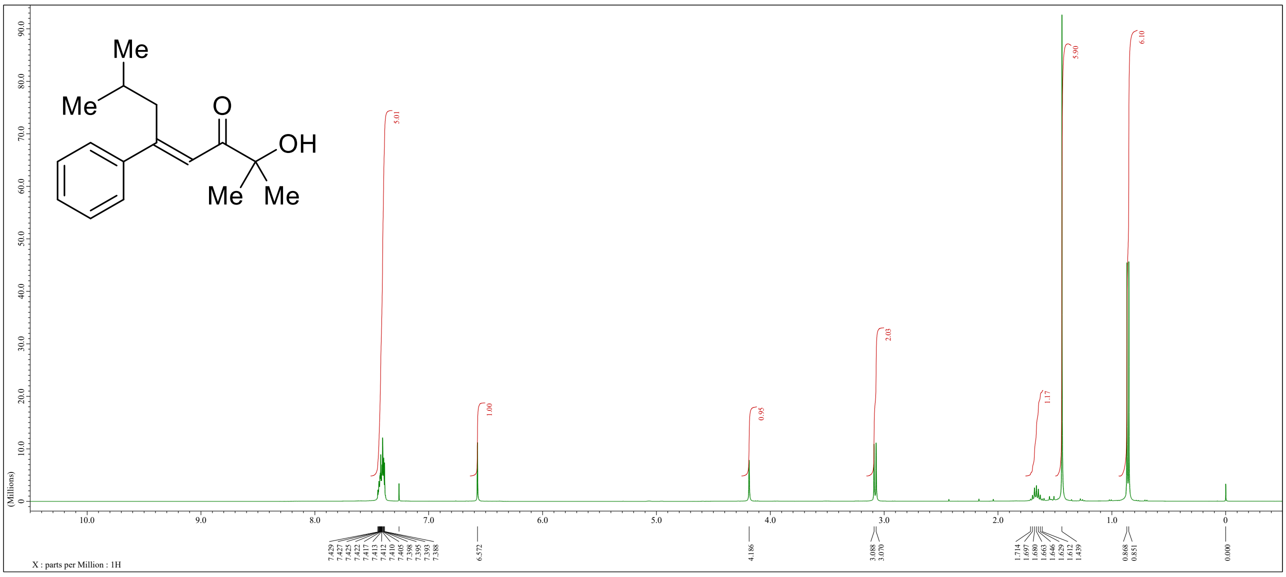

$^{13}C\{^{1}H\}$ NMR (100 MHz, $CDCl_3$) (*E*)-**1l**

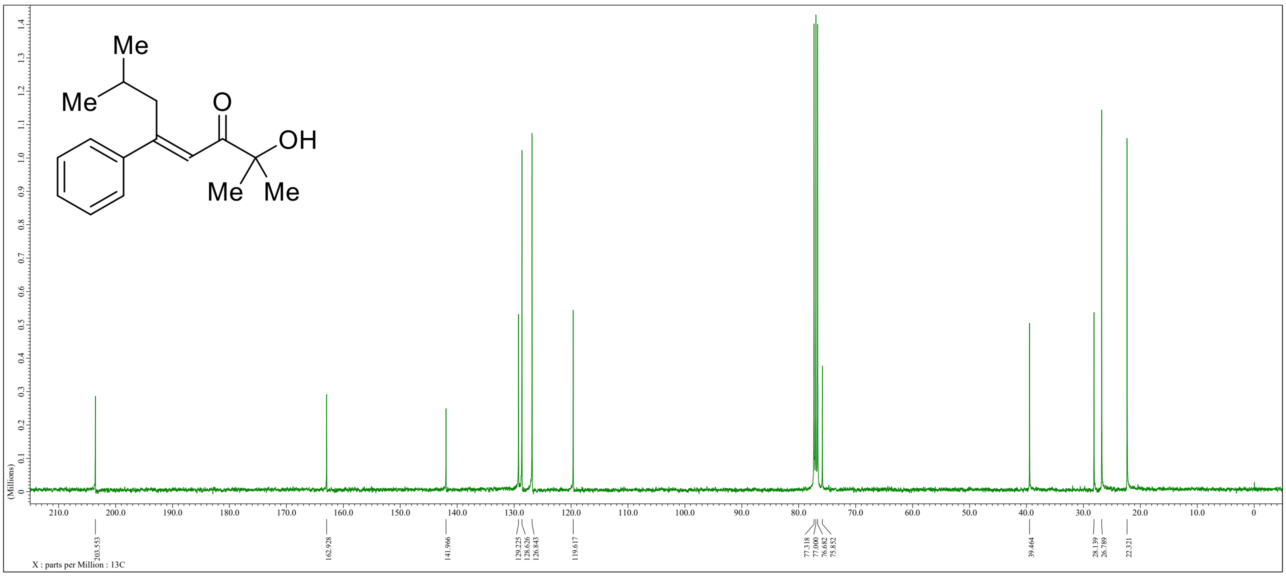

^{1}H NMR (400 MHz, $CDCl_3$) (*E*)-**1m**

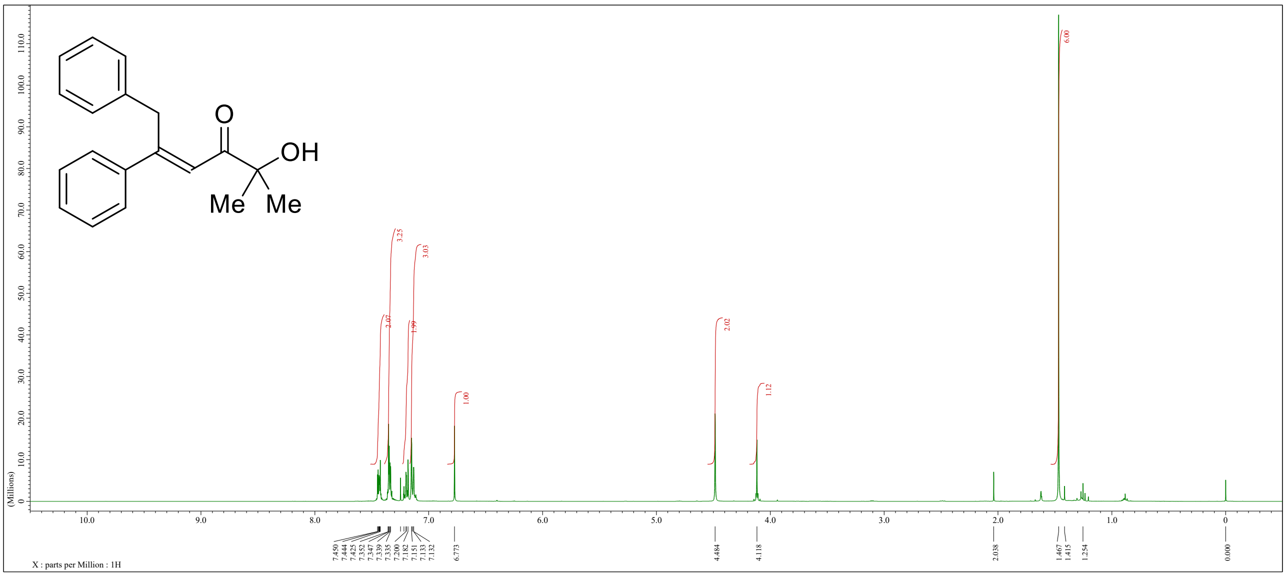

^{13}C{^{1}H} NMR (100 MHz, $CDCl_3$) (*E*)-**1m**

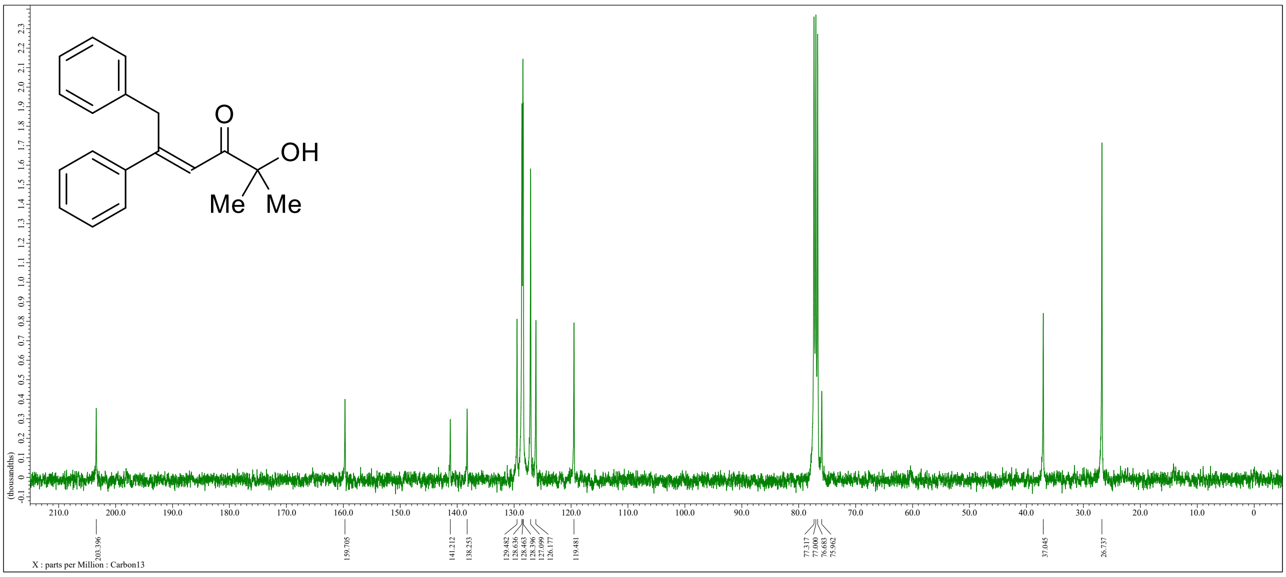

^{1}H NMR (400 MHz, CDCl$_3$) (*E*)-**1n**

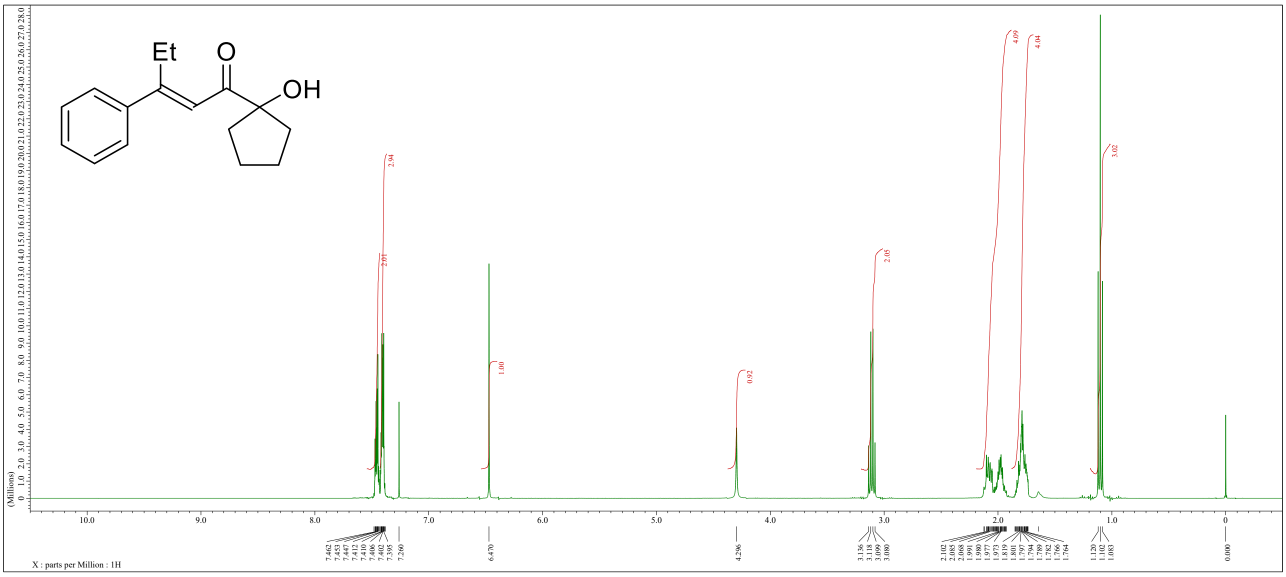

^{13}C{^{1}H} NMR (100 MHz, CDCl$_3$) (*E*)-**1n**

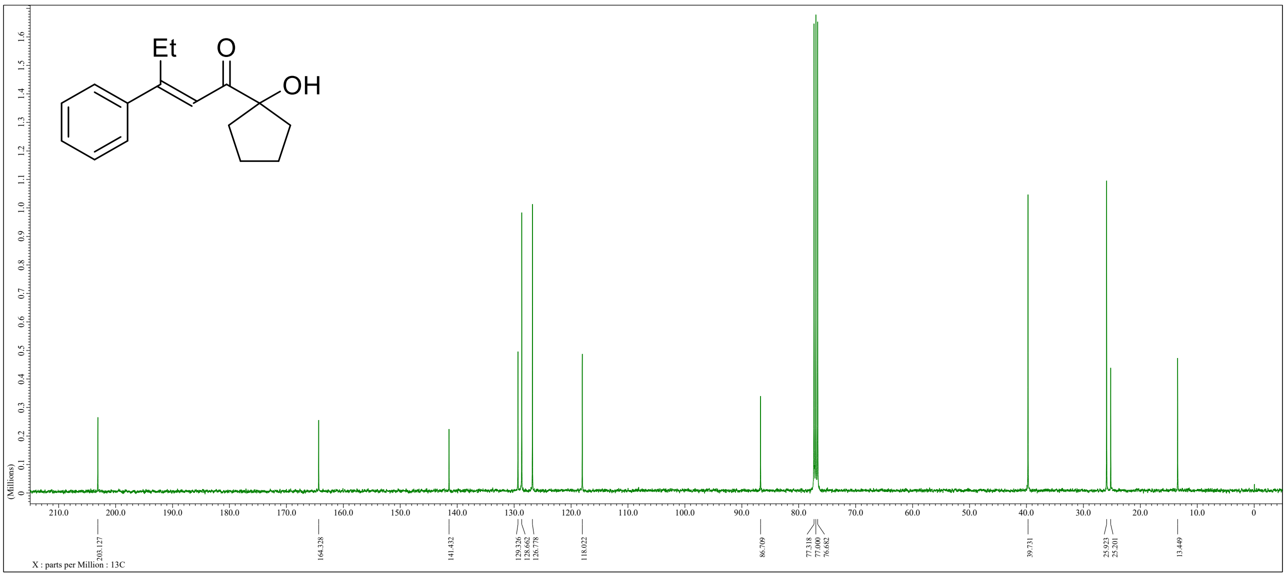

^{1}H NMR (400 MHz, CDCl$_3$) (*E*)-**1o**

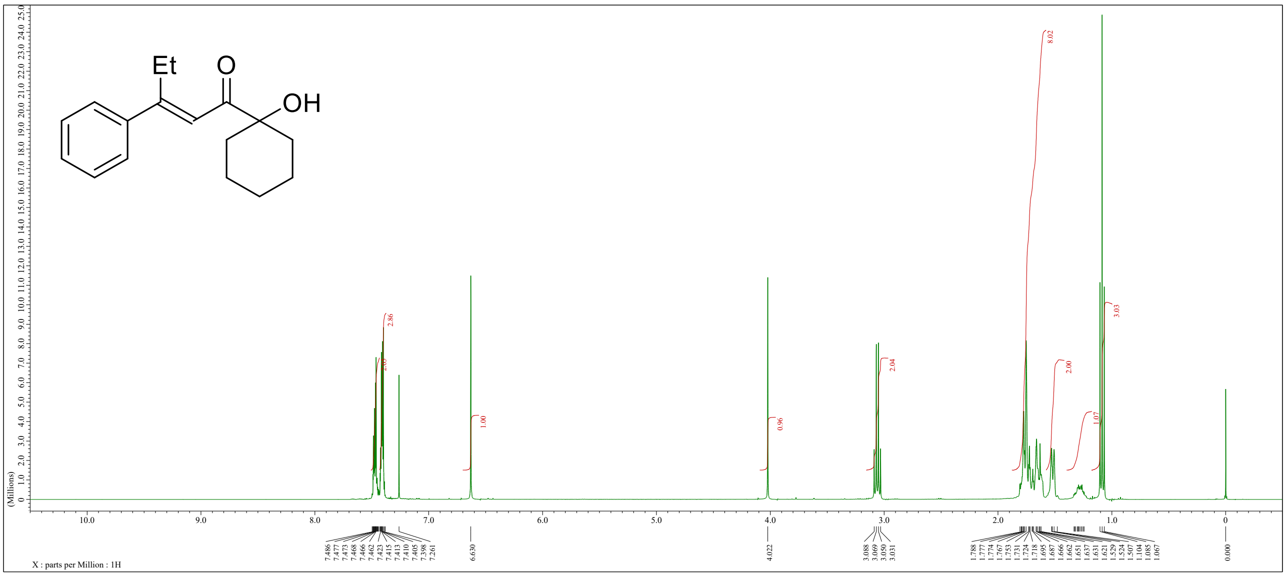

^{13}C{^{1}H} NMR (100 MHz, CDCl$_3$) (*E*)-**1o**

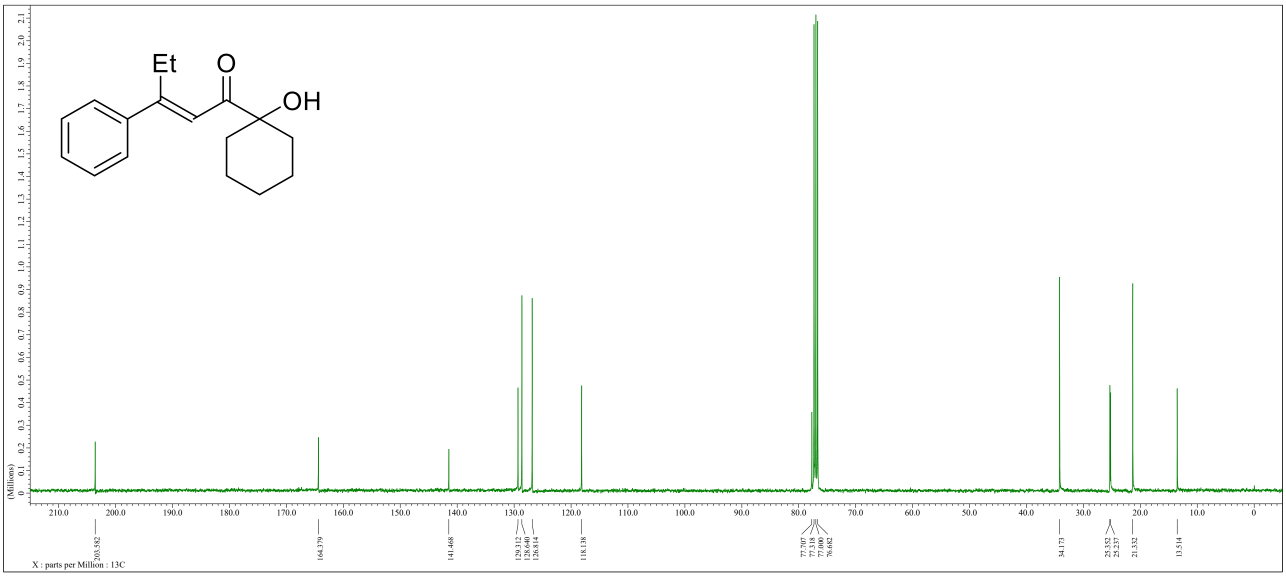

^{1}H NMR (400 MHz, $CDCl_3$) (*E*)-**1p**

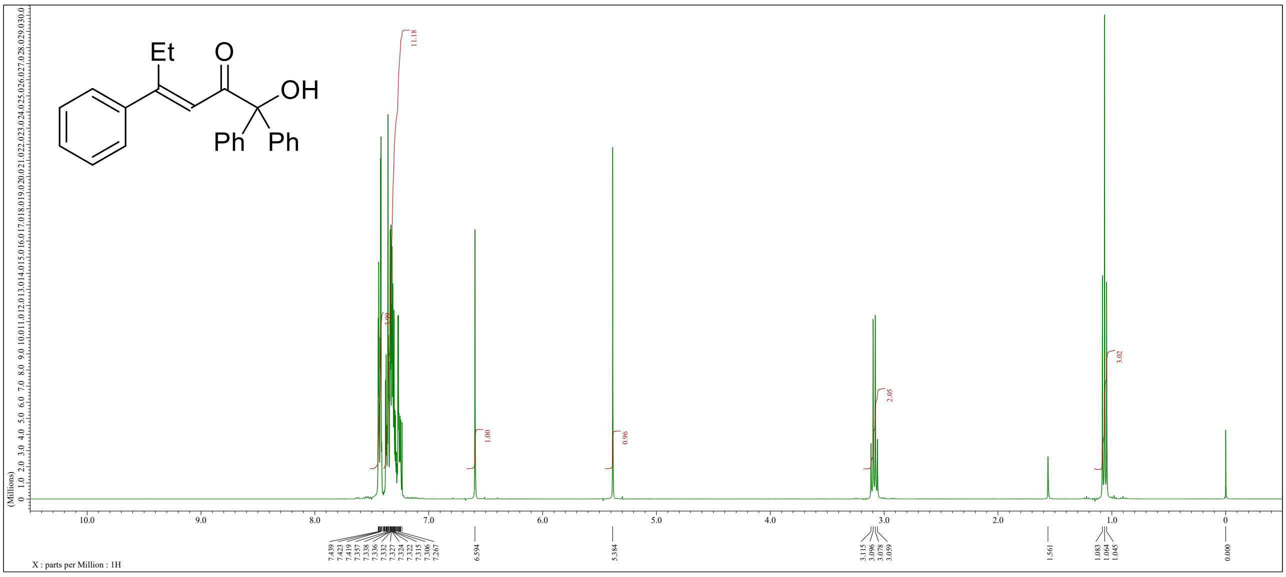

$^{13}C\{^{1}H\}$ NMR (100 MHz, $CDCl_3$) (*E*)-**1p**

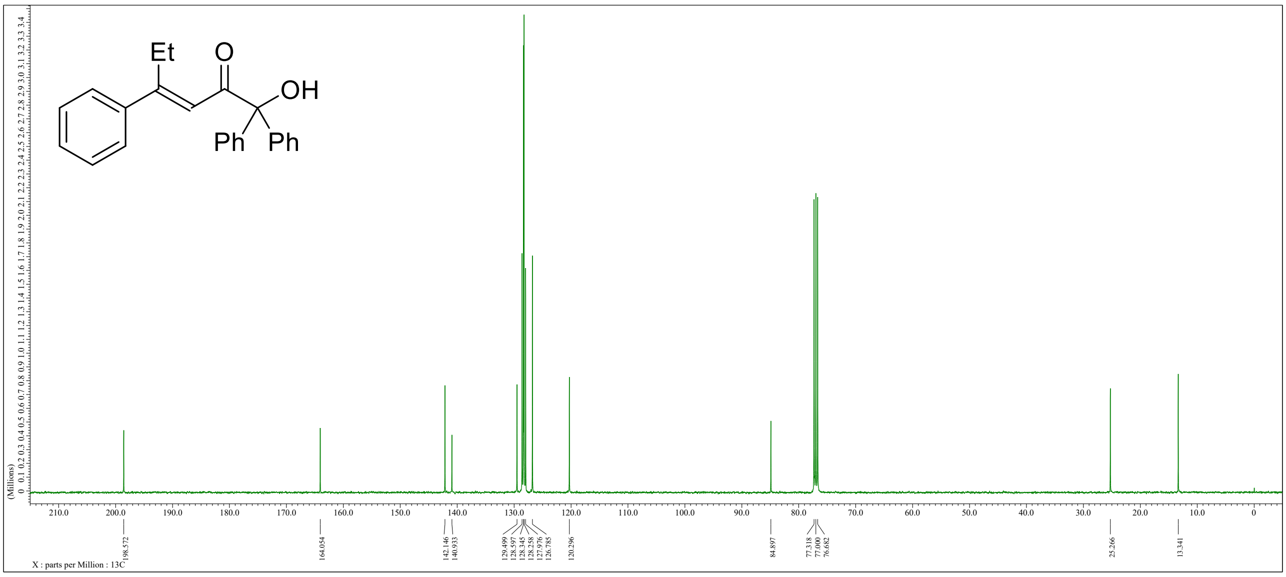

^{1}H NMR (400 MHz, $CDCl_3$) (*E*)-**3**

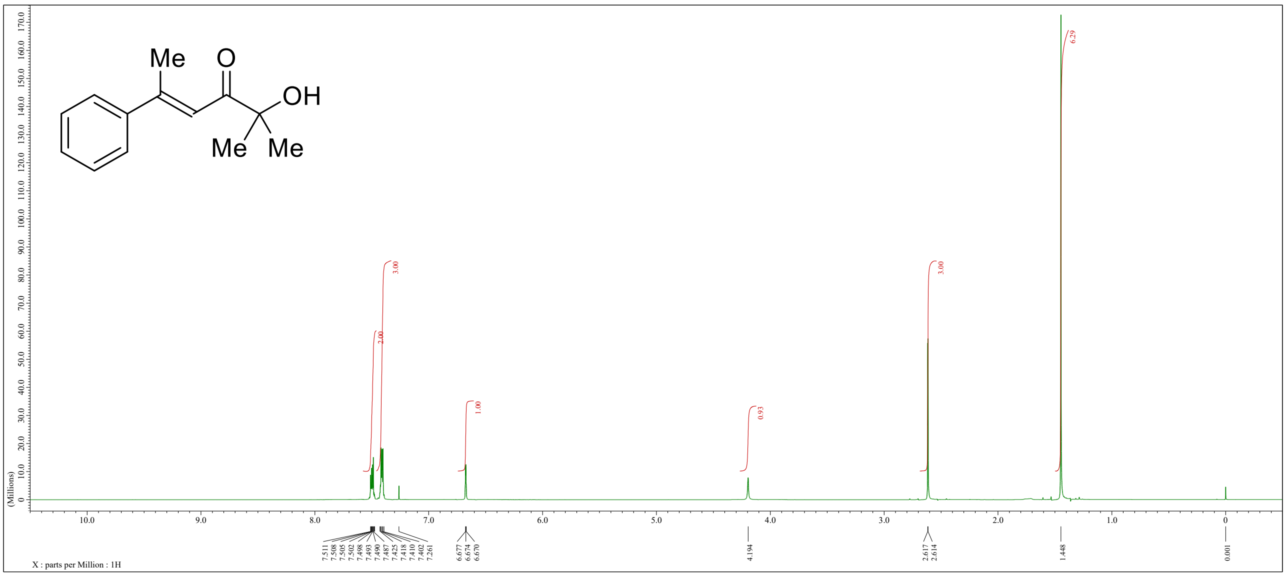

$^{13}C\{^{1}H\}$ NMR (100 MHz, $CDCl_3$) (*E*)-**3**

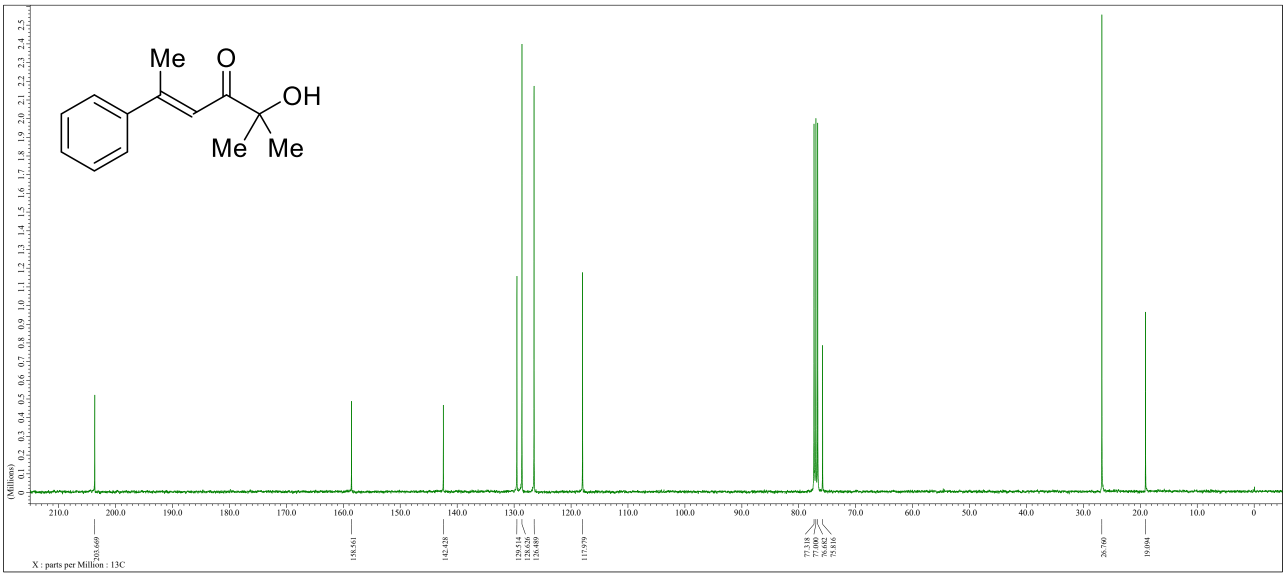

^{1}H NMR (400 MHz, $CDCl_3$) (*E*)-**4**

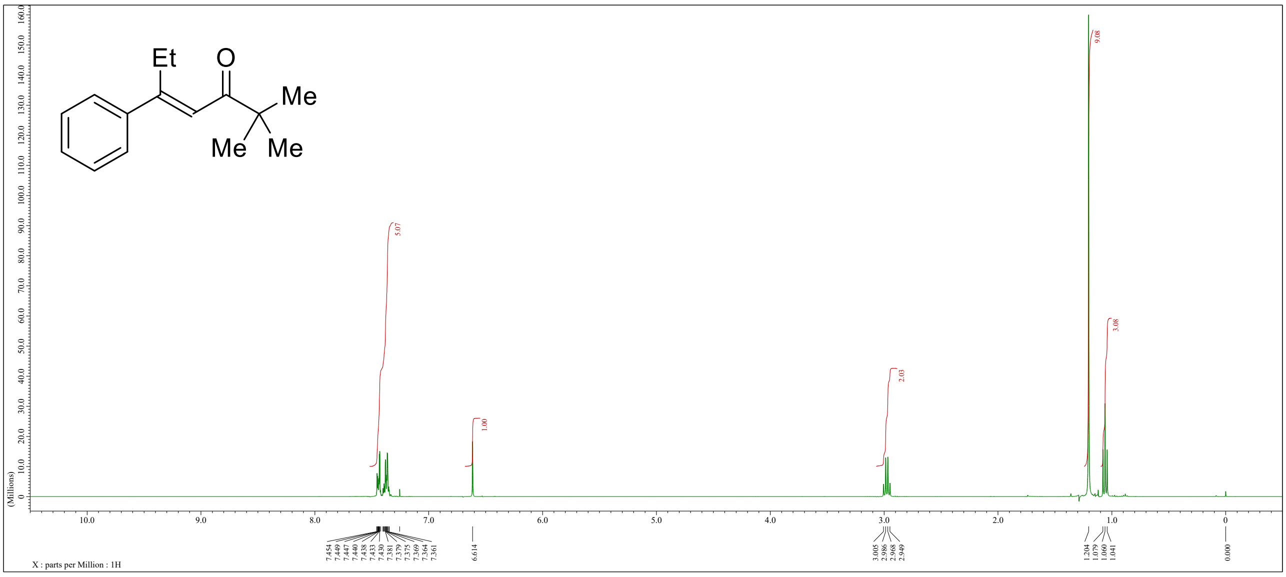

$^{13}C\{^{1}H\}$ NMR (100 MHz, $CDCl_3$) (*E*)-**4**

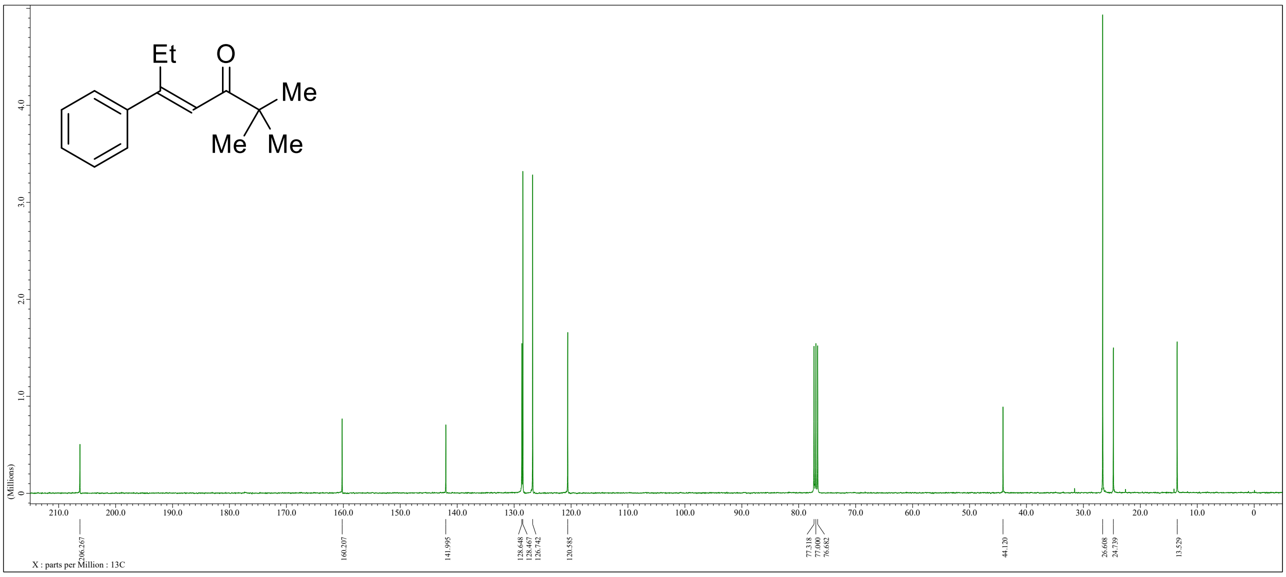

^{1}H NMR (400 MHz, $CDCl_3$) (*E*)-**6**

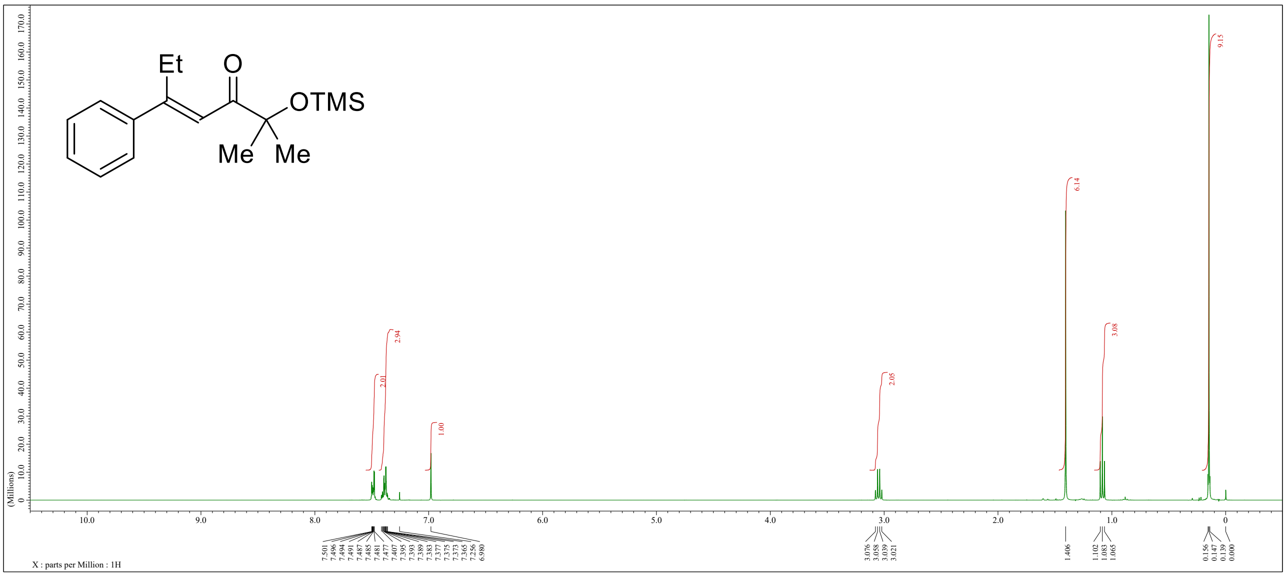

^{13}C{^{1}H} NMR (100 MHz, $CDCl_3$) (*E*)-**6**

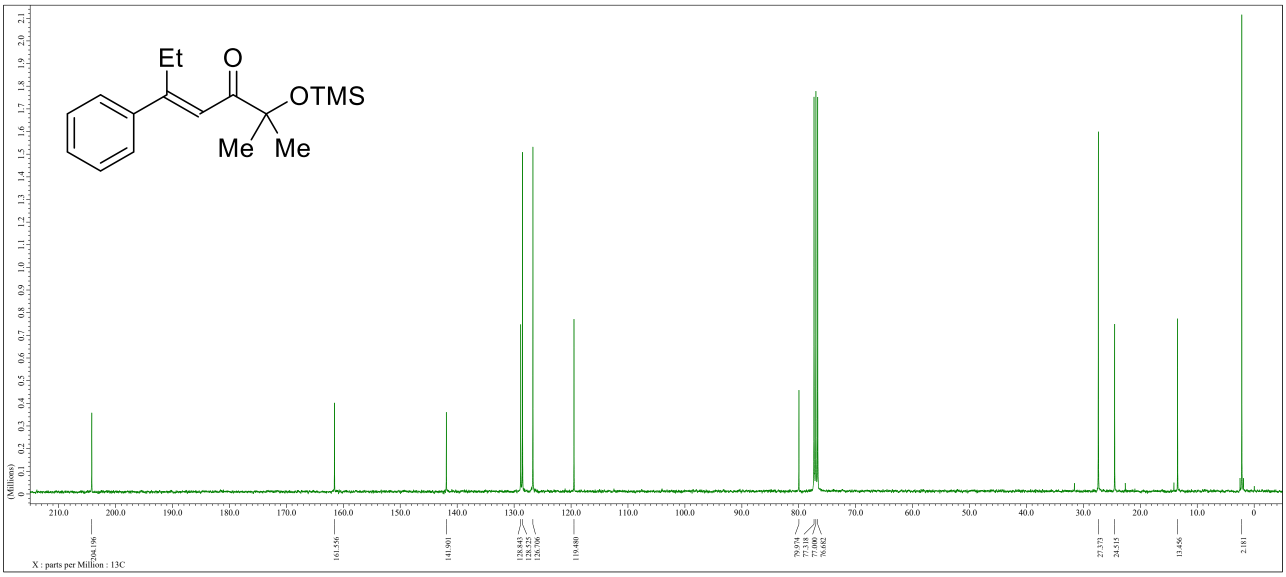

^{1}H NMR (400 MHz, $CDCl_3$) **2a**

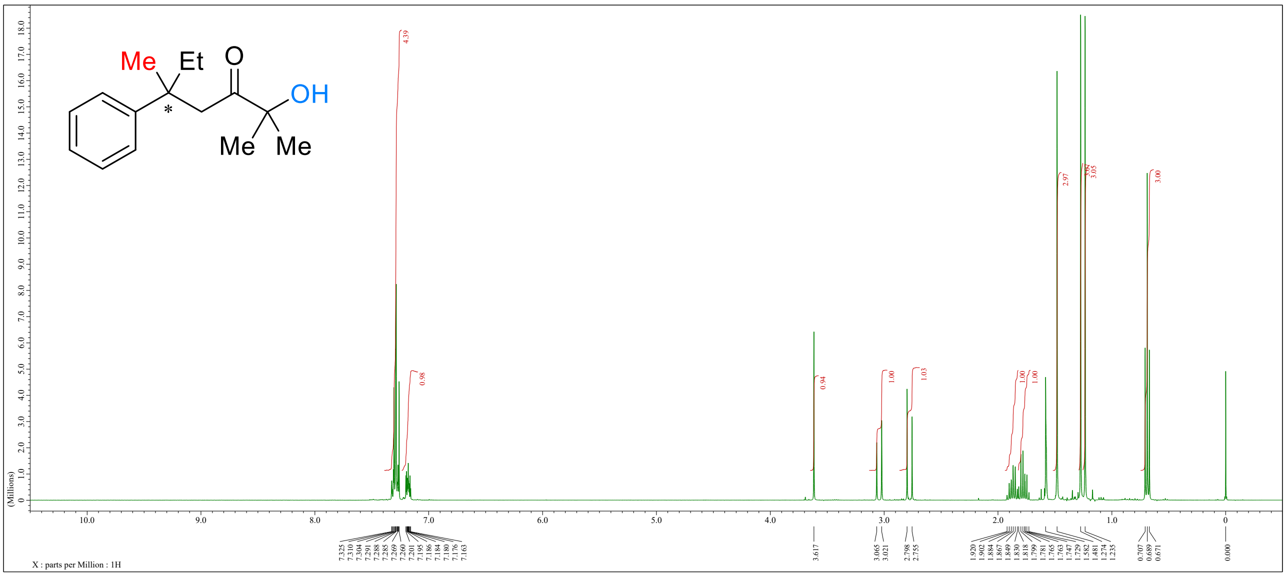

^{13}C{^{1}H} NMR (100 MHz, $CDCl_3$) **2a**

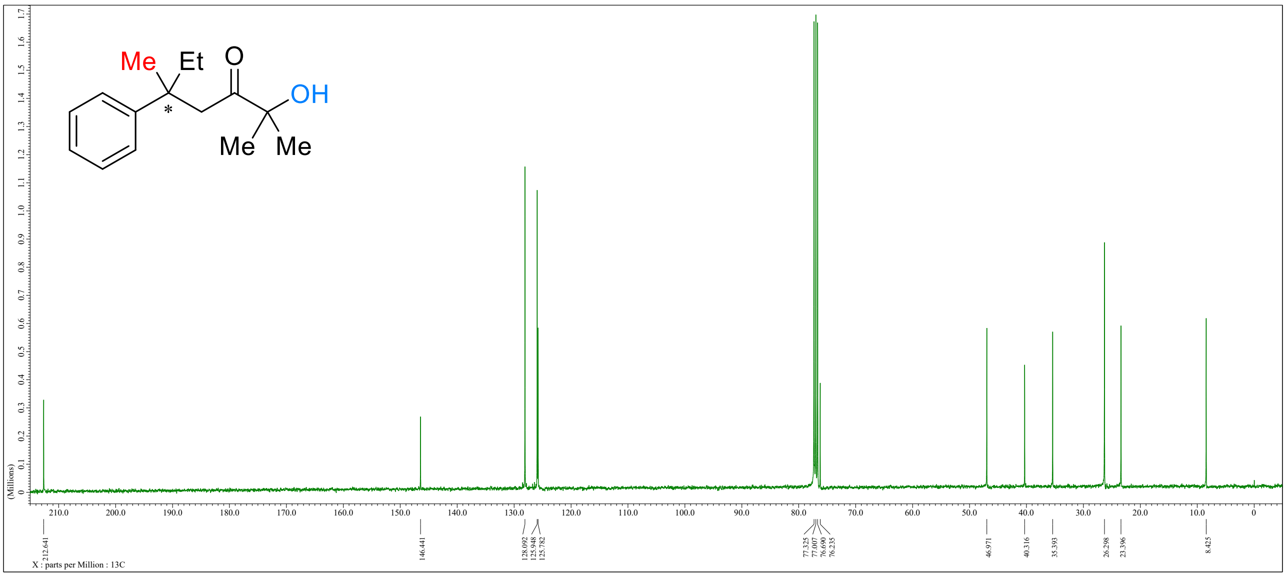

^{1}H NMR (400 MHz, $CDCl_3$) **2b**

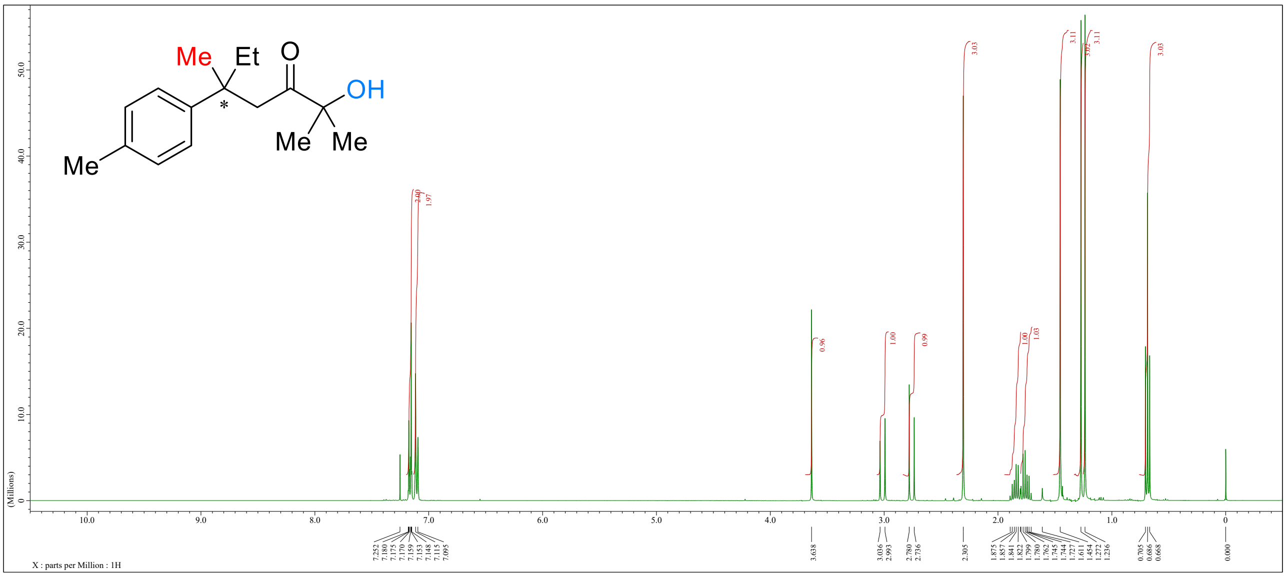

$^{13}C\{^{1}H\}$ NMR (100 MHz, $CDCl_3$) **2b**

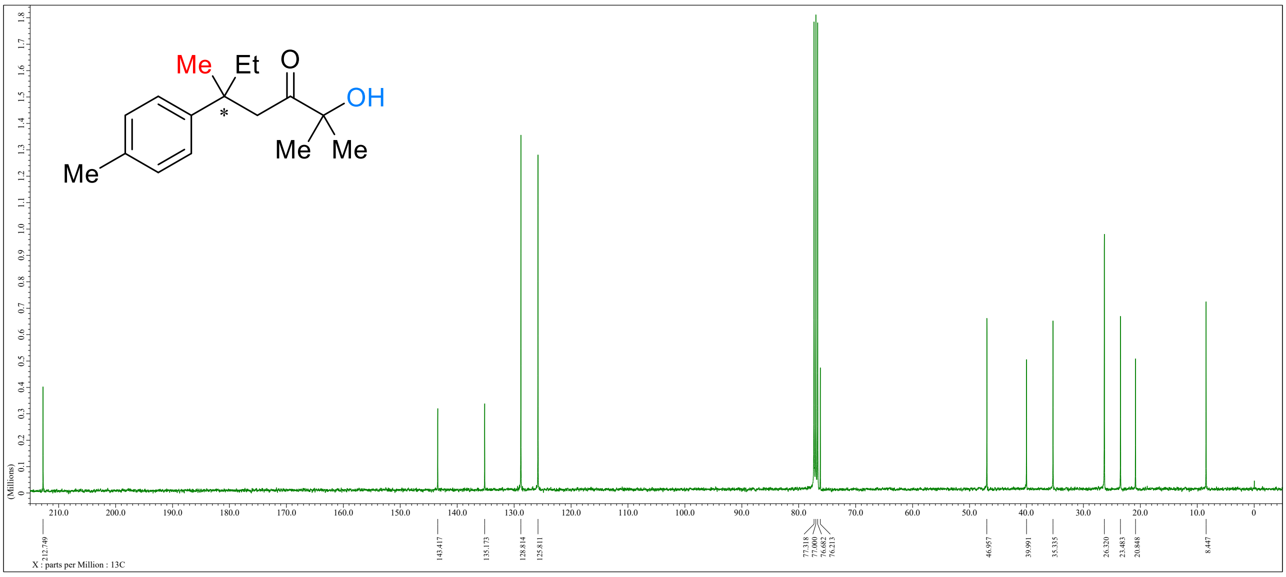

^{1}H NMR (400 MHz, $CDCl_3$) **2c**

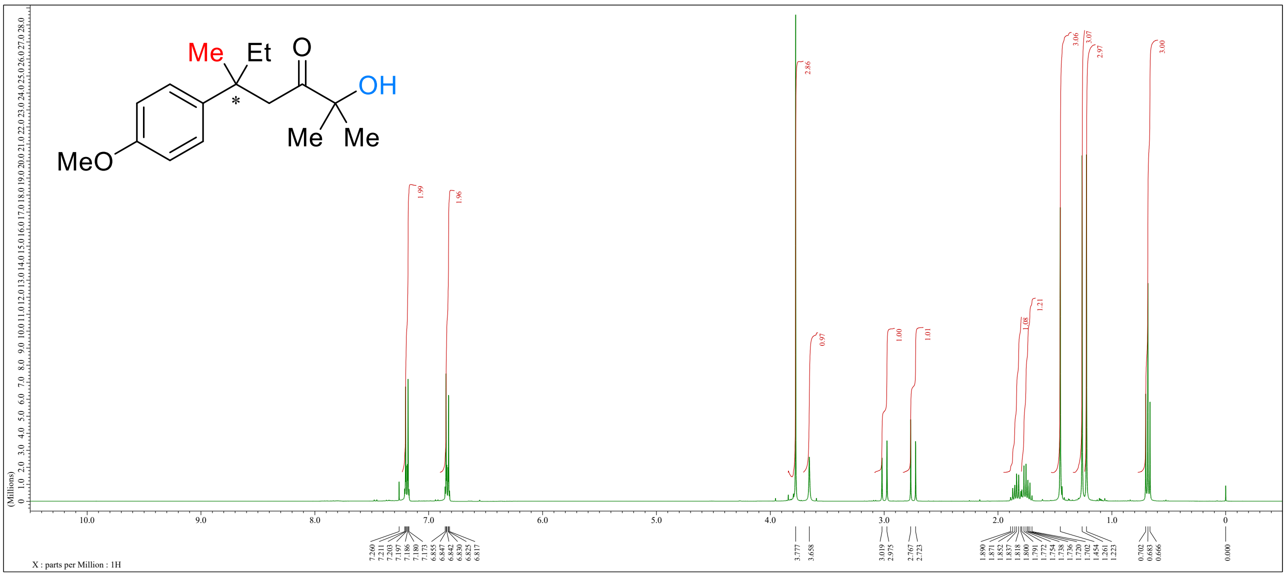

$^{13}C\{^{1}H\}$ NMR (100 MHz, $CDCl_3$) **2c**

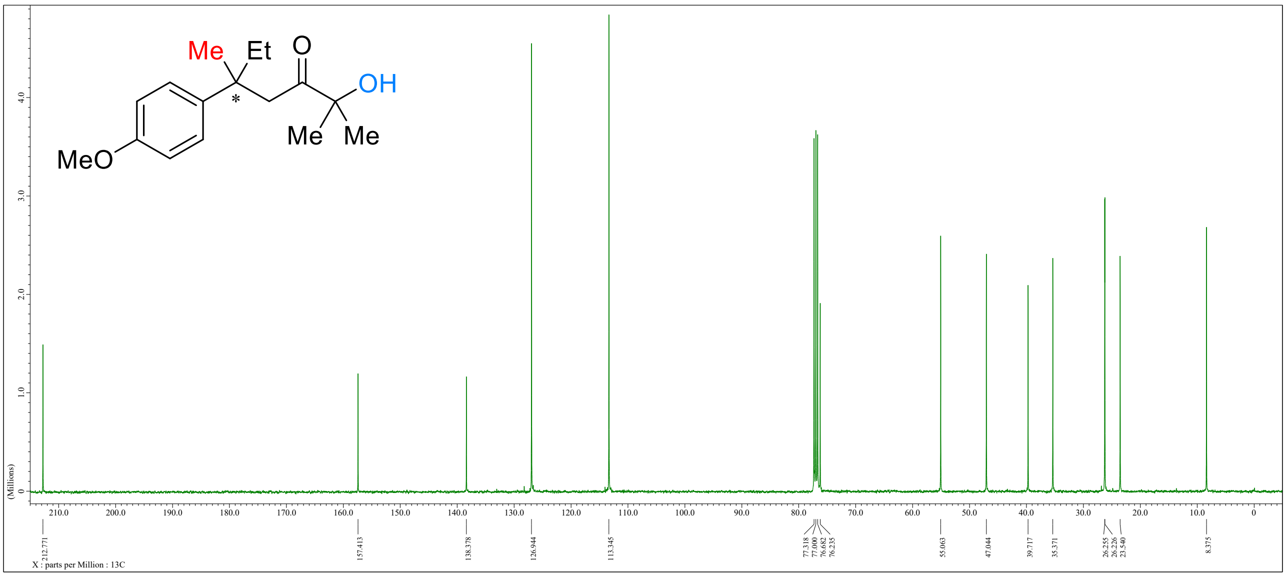

^{1}H NMR (400 MHz, $CDCl_3$) **2d**

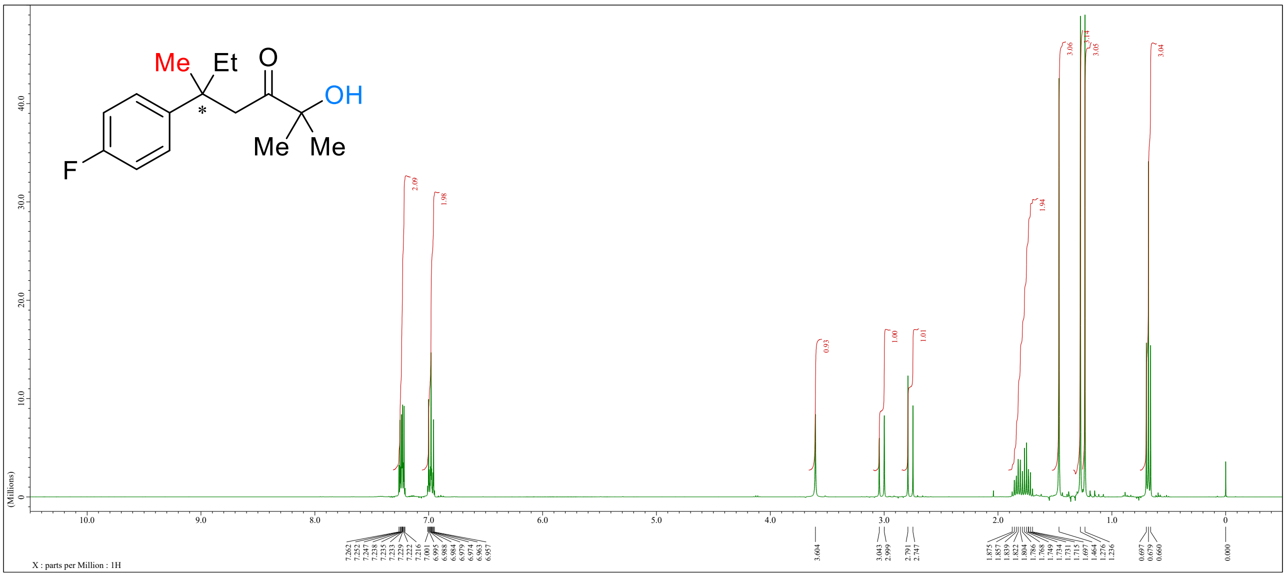

$^{13}C\{^{1}H\}$ NMR (100 MHz, $CDCl_3$) **2d**

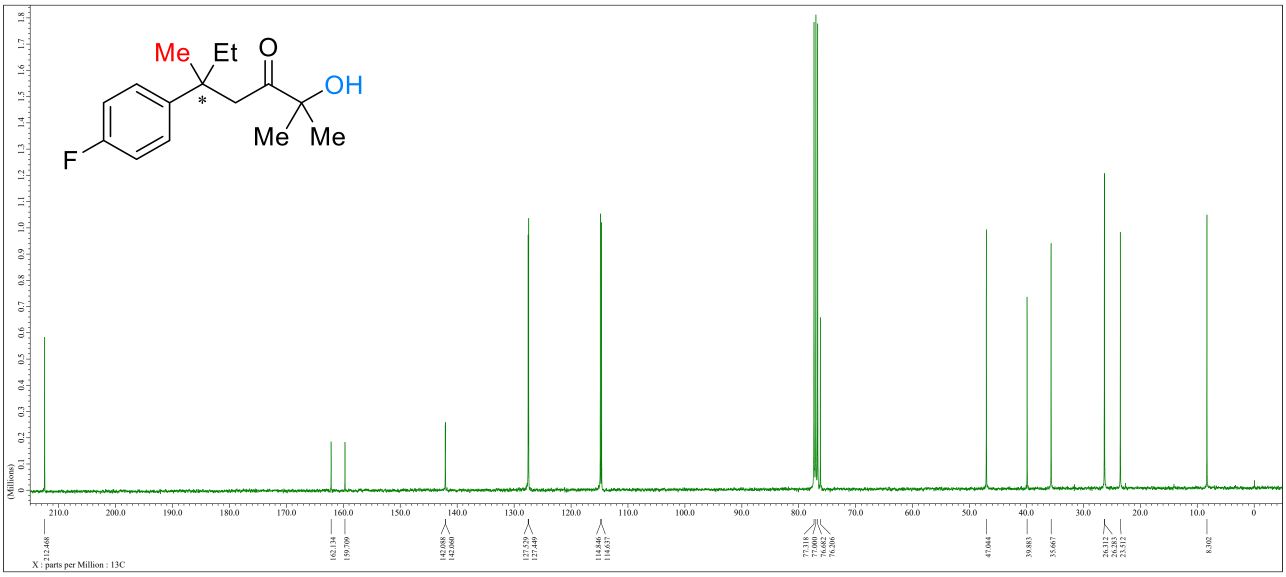

^{19}F NMR (375 MHz, $CDCl_3$) **2d**

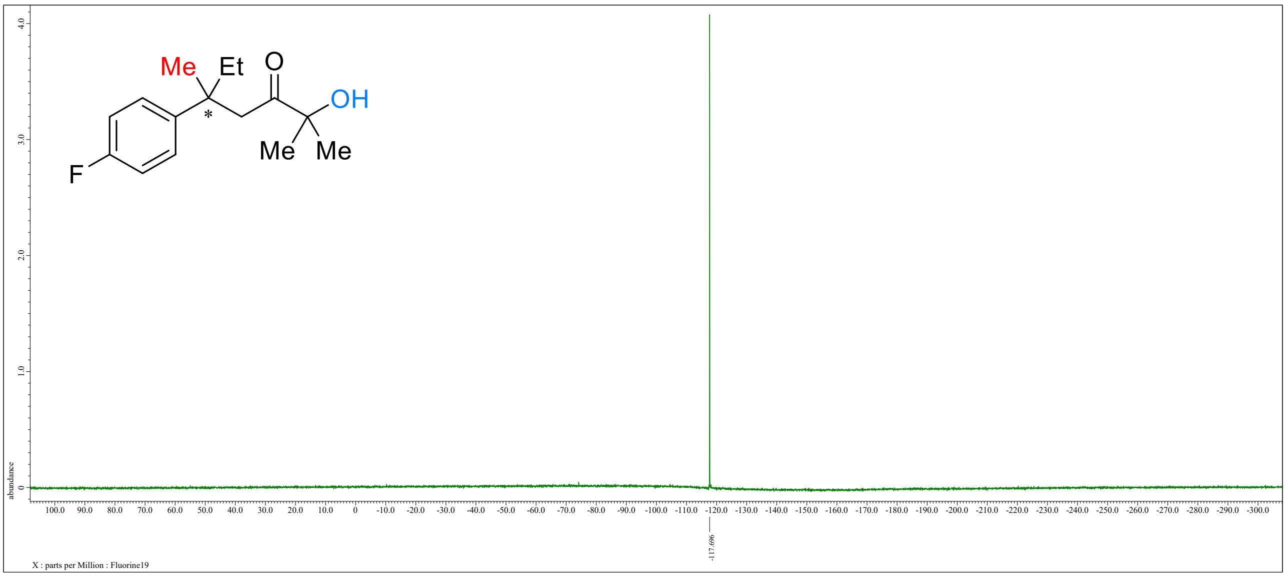

^{1}H NMR (400 MHz, $CDCl_3$) **2e**

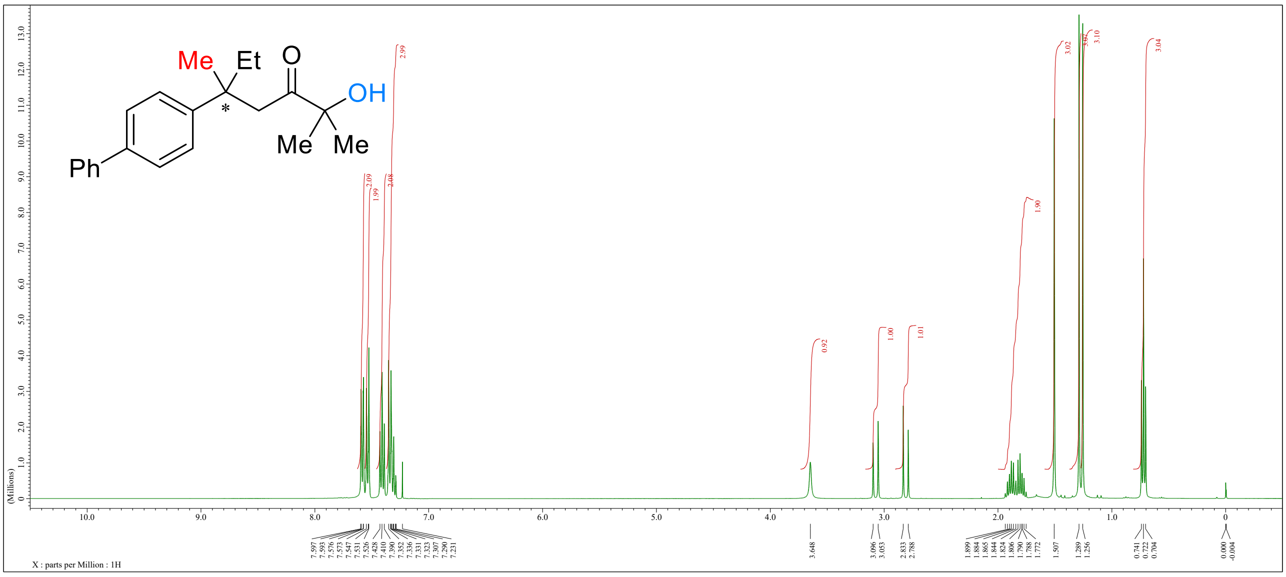

$^{13}C\{^{1}H\}$ NMR (100 MHz, $CDCl_3$) **2e**

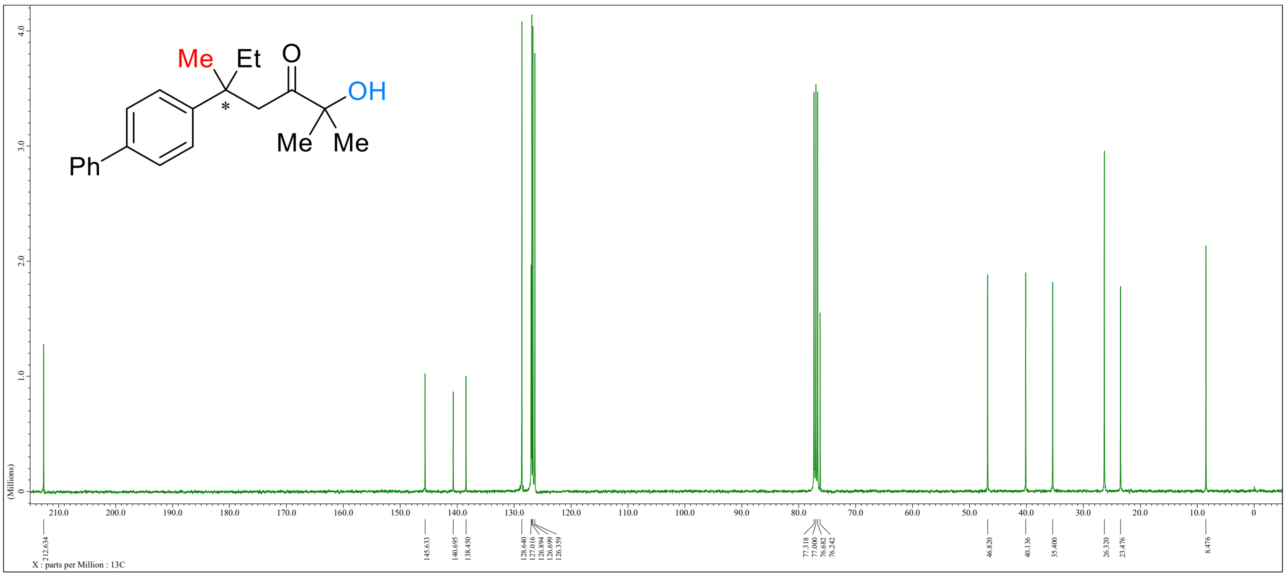

^{1}H NMR (400 MHz, $CDCl_3$) **2f**

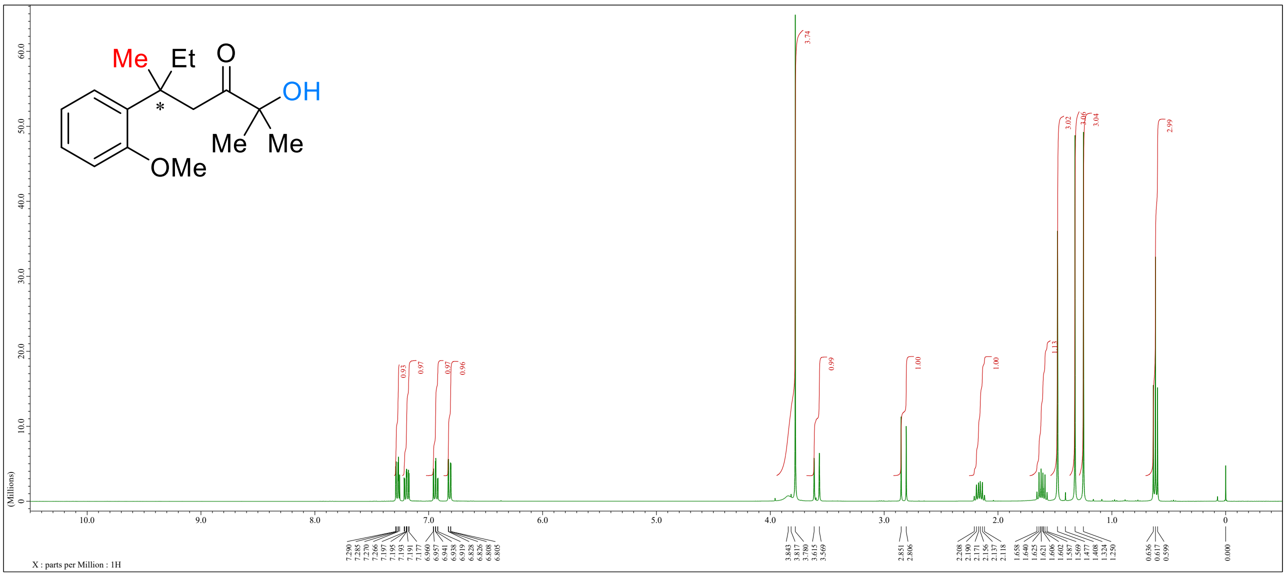

$^{13}C\{^{1}H\}$ NMR (100 MHz, $CDCl_3$) **2f**

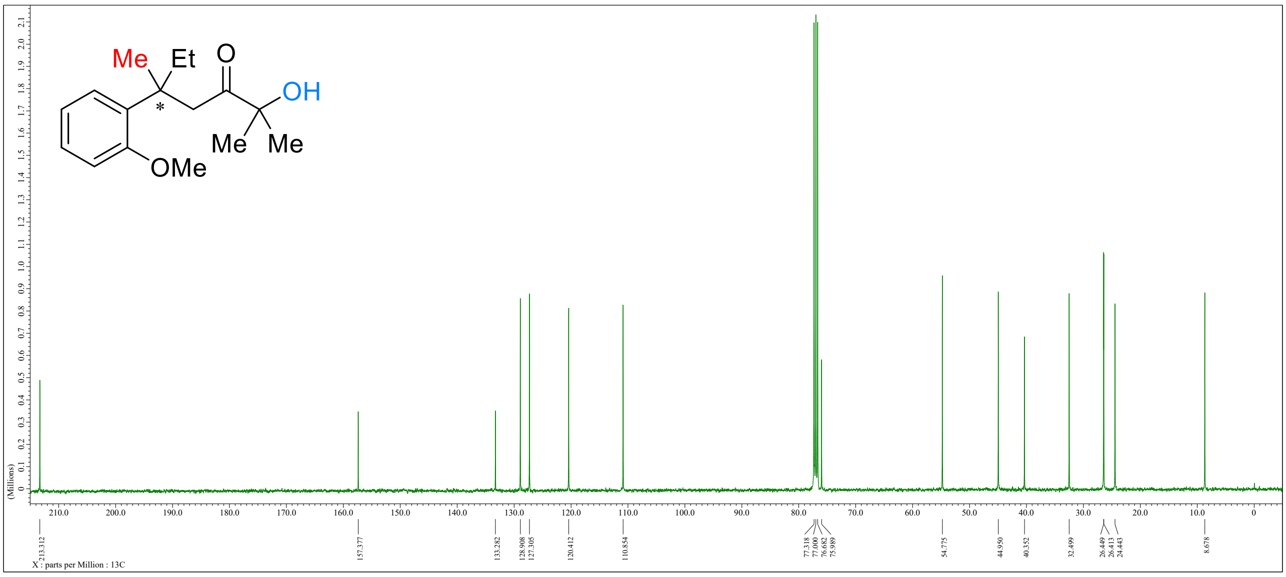

^{1}H NMR (400 MHz, $CDCl_3$) **2g**

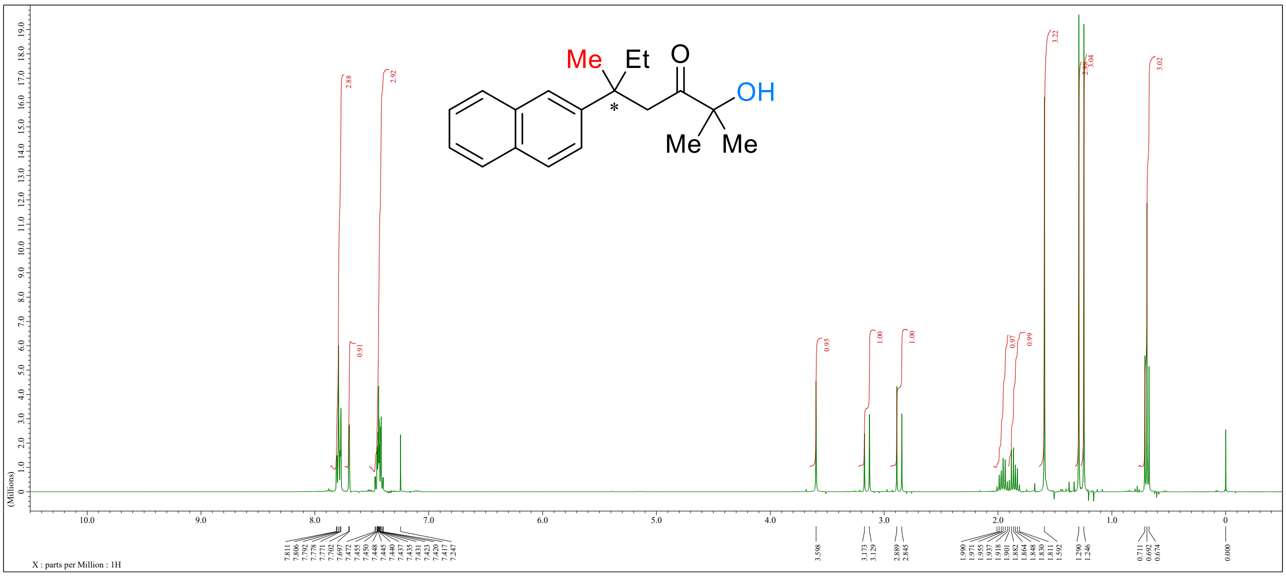

^{13}C{^{1}H} NMR (100 MHz, $CDCl_3$) **2g**

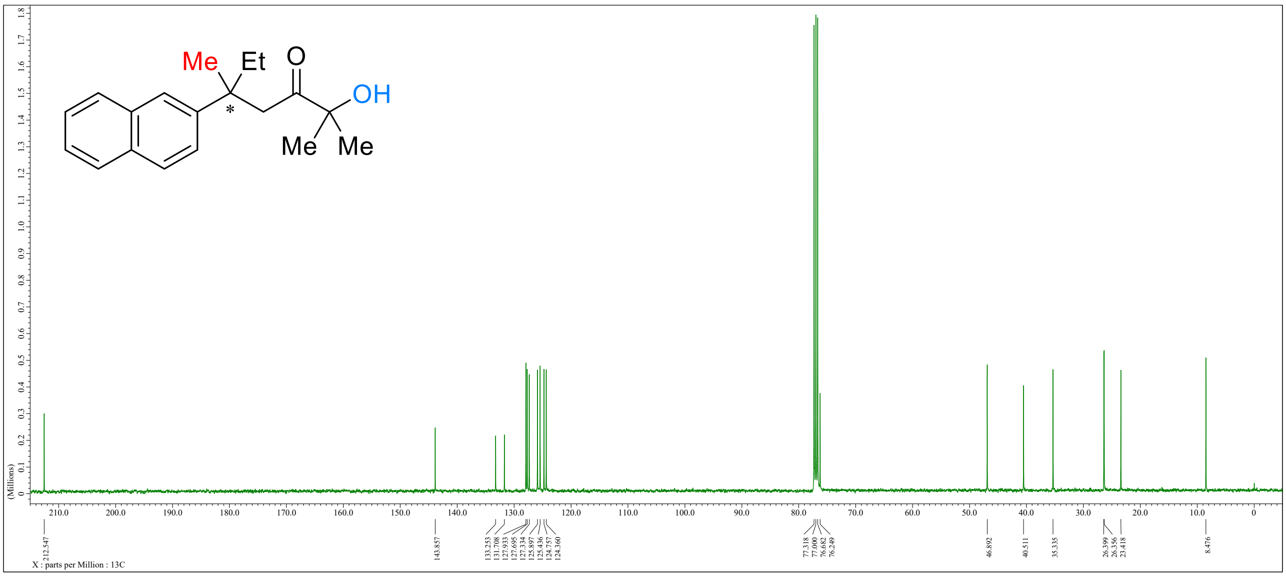

^{1}H NMR (400 MHz, $CDCl_3$) **2h**

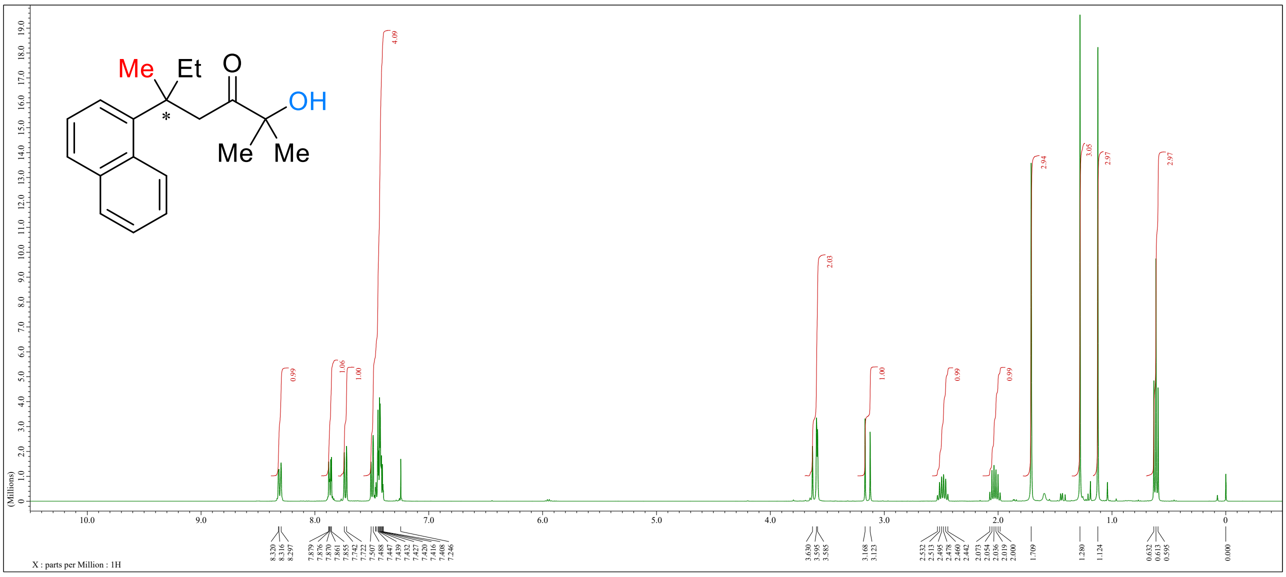

$^{13}C\{^{1}H\}$ NMR (100 MHz, $CDCl_3$) **2h**

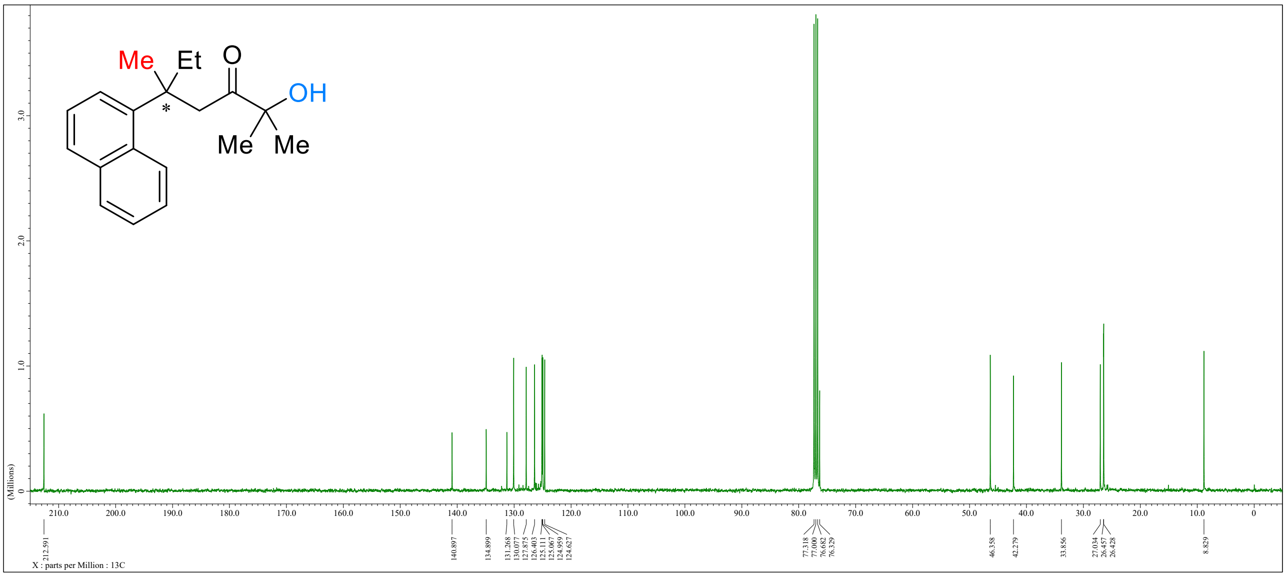

^{1}H NMR (400 MHz, $CDCl_3$) **2i**

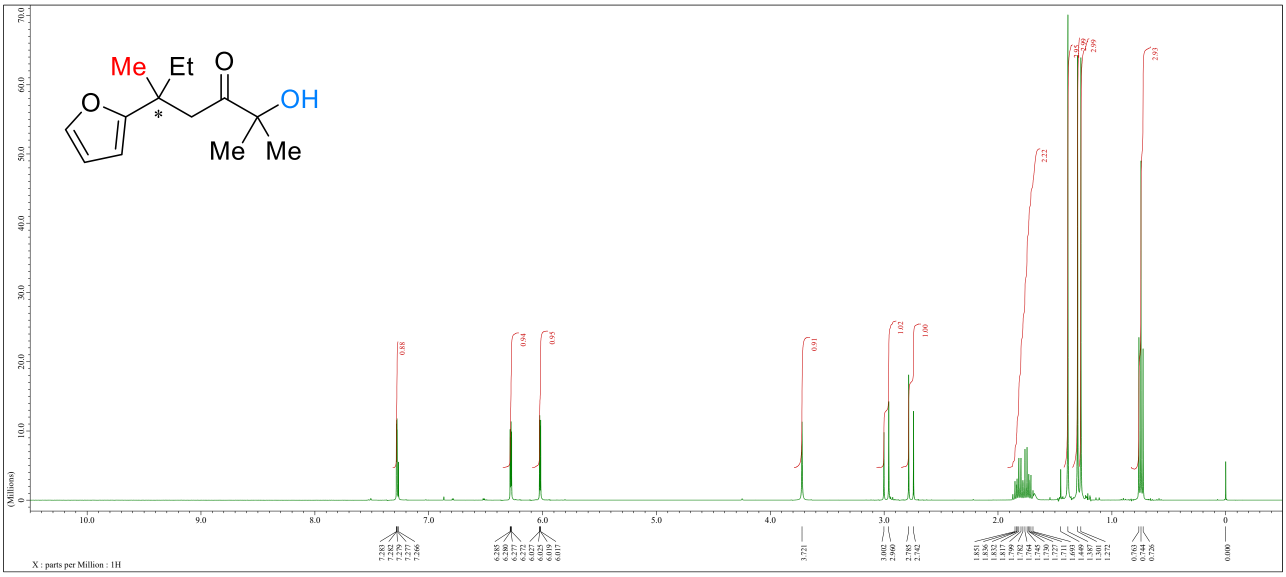

^{13}C{^{1}H} NMR (100 MHz, $CDCl_3$) **2i**

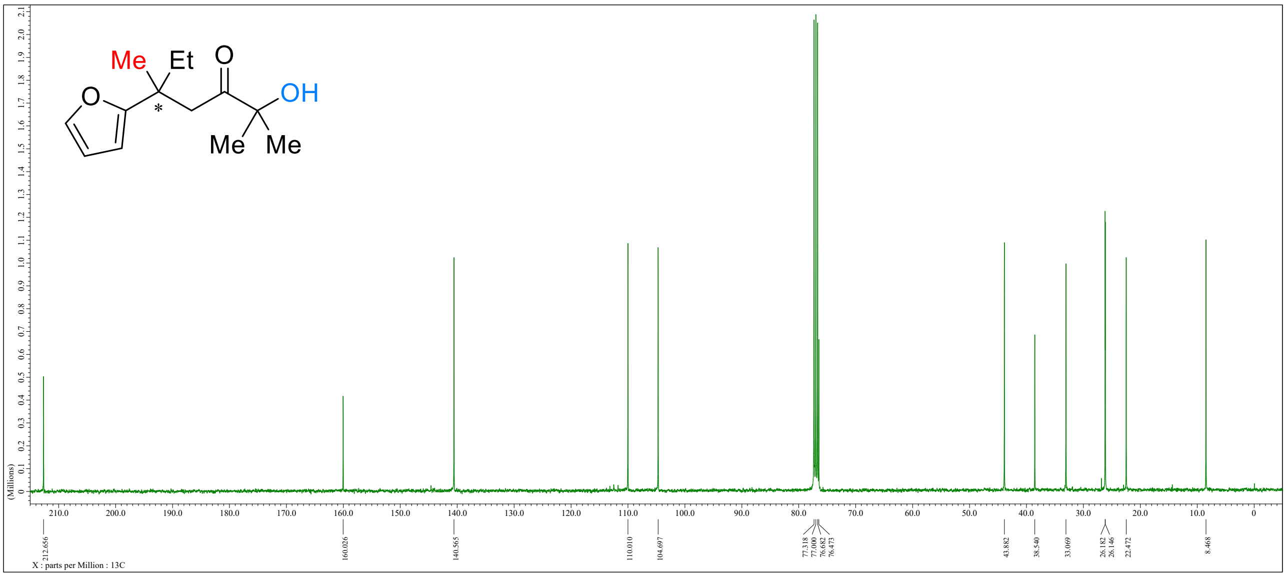

^{1}H NMR (400 MHz, $CDCl_3$) **2j**

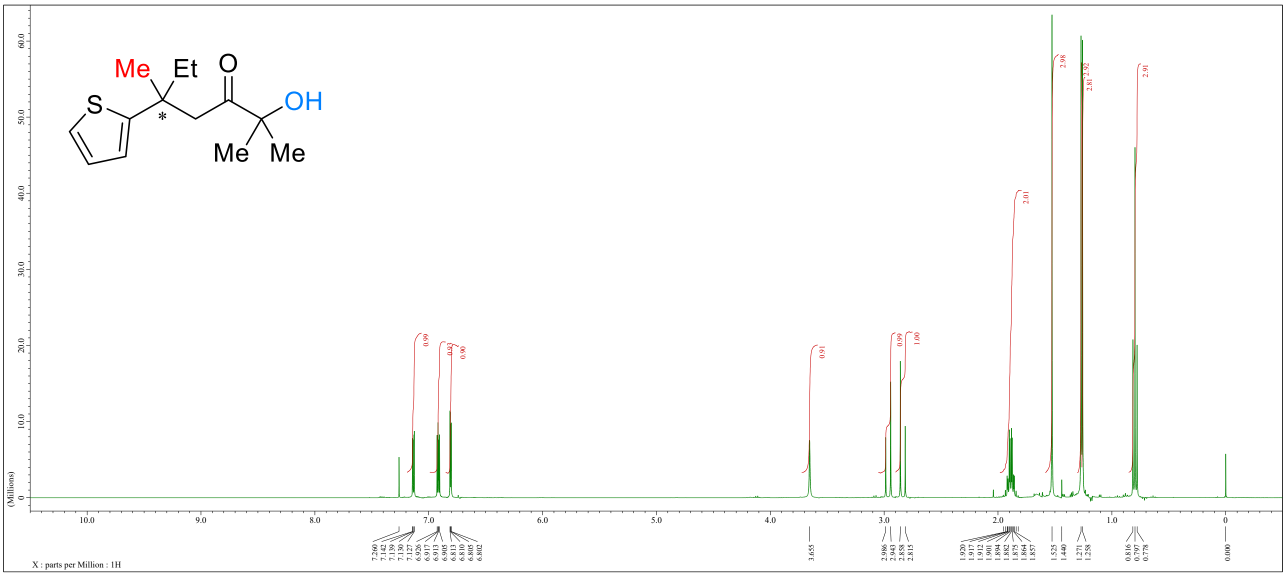

$^{13}C\{^{1}H\}$ NMR (100 MHz, $CDCl_3$) **2j**

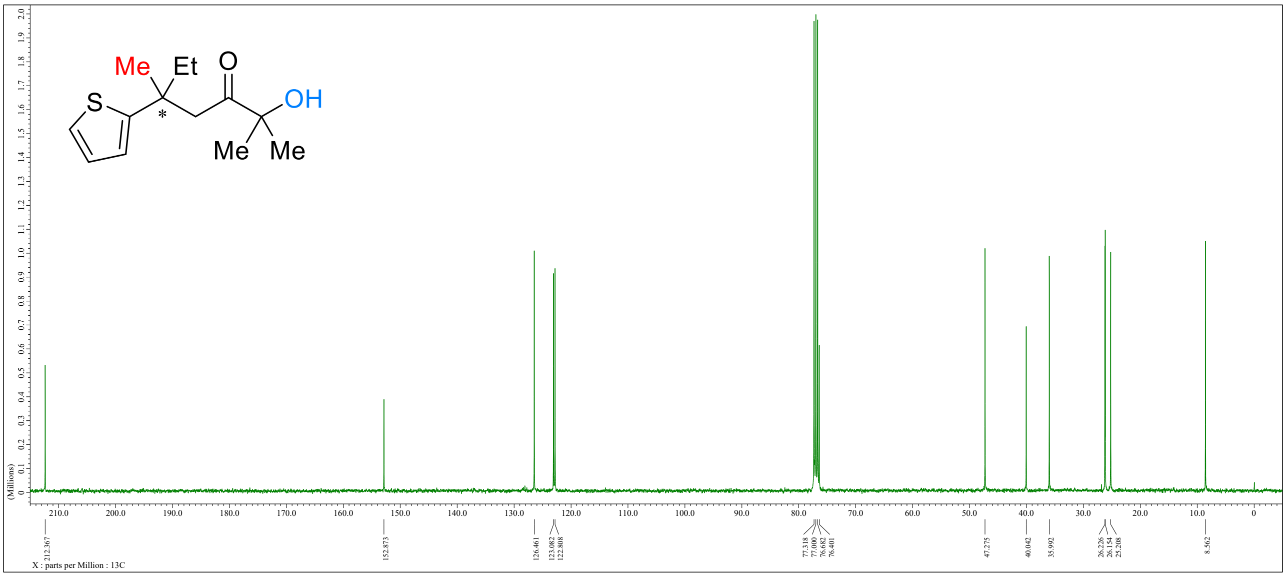

^{1}H NMR (400 MHz, $CDCl_3$) **2k**

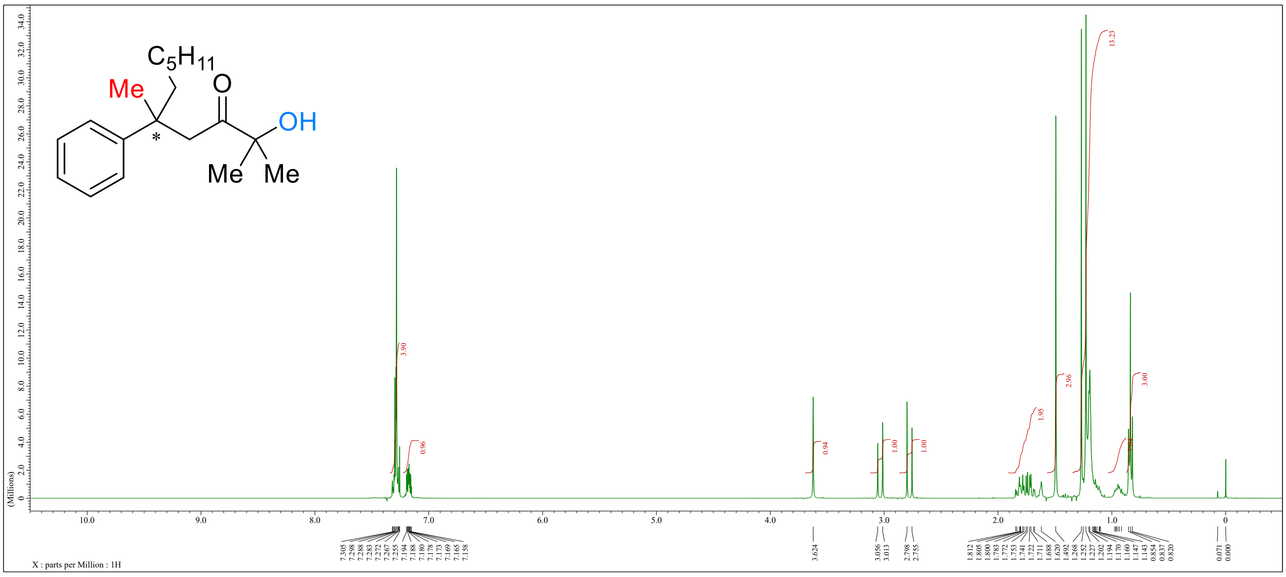

^{13}C{^{1}H} NMR (100 MHz, $CDCl_3$) **2k**

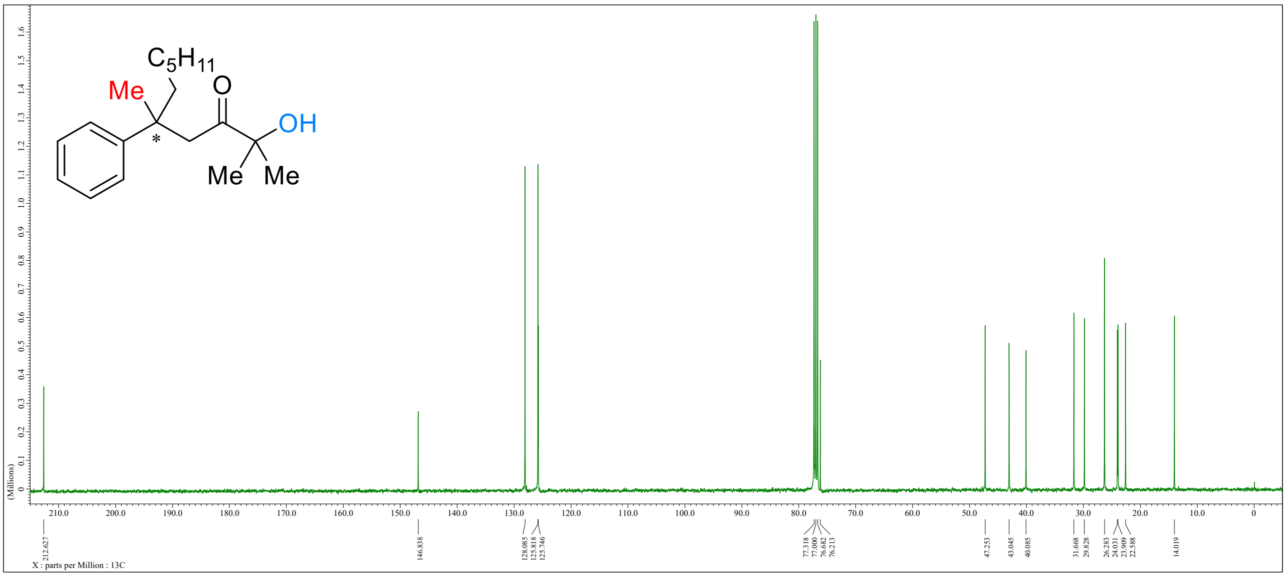

^{1}H NMR (400 MHz, $CDCl_3$) **2l**

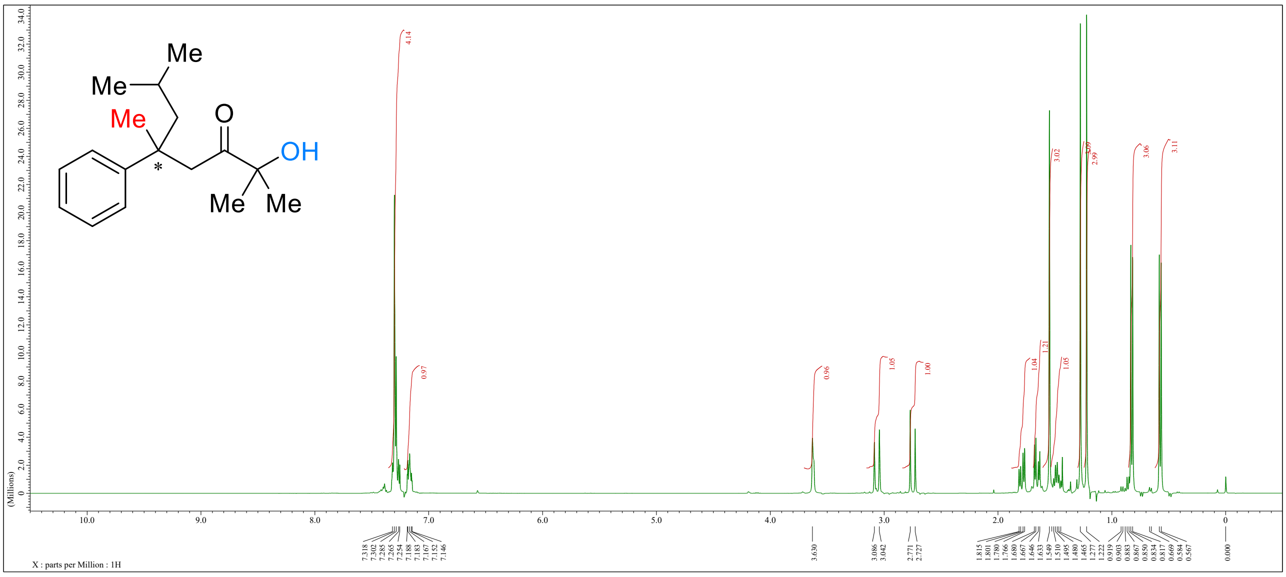

$^{13}C\{^{1}H\}$ NMR (100 MHz, $CDCl_3$) **2l**

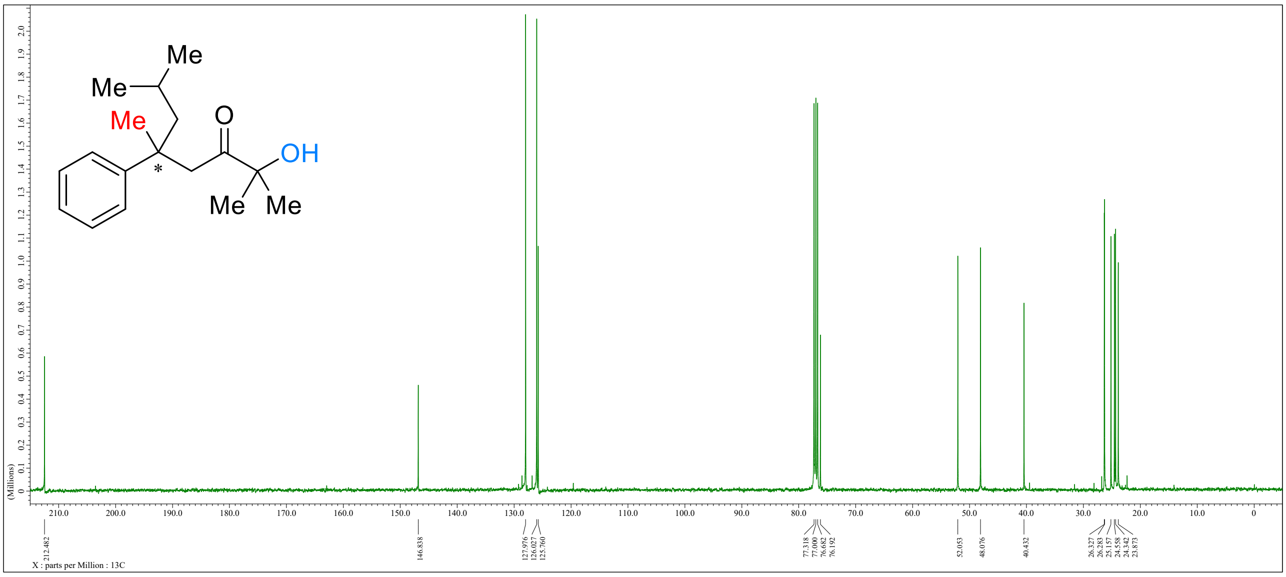

^{1}H NMR (400 MHz, $CDCl_3$) **2m**

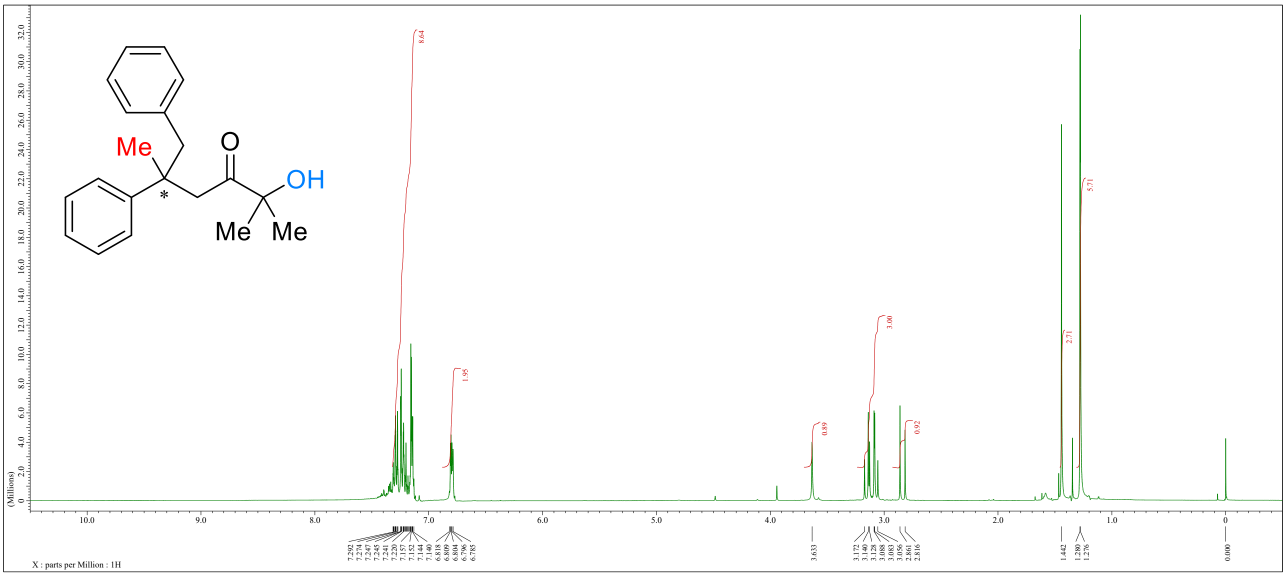

$^{13}C\{^{1}H\}$ NMR (100 MHz, $CDCl_3$) **2m**

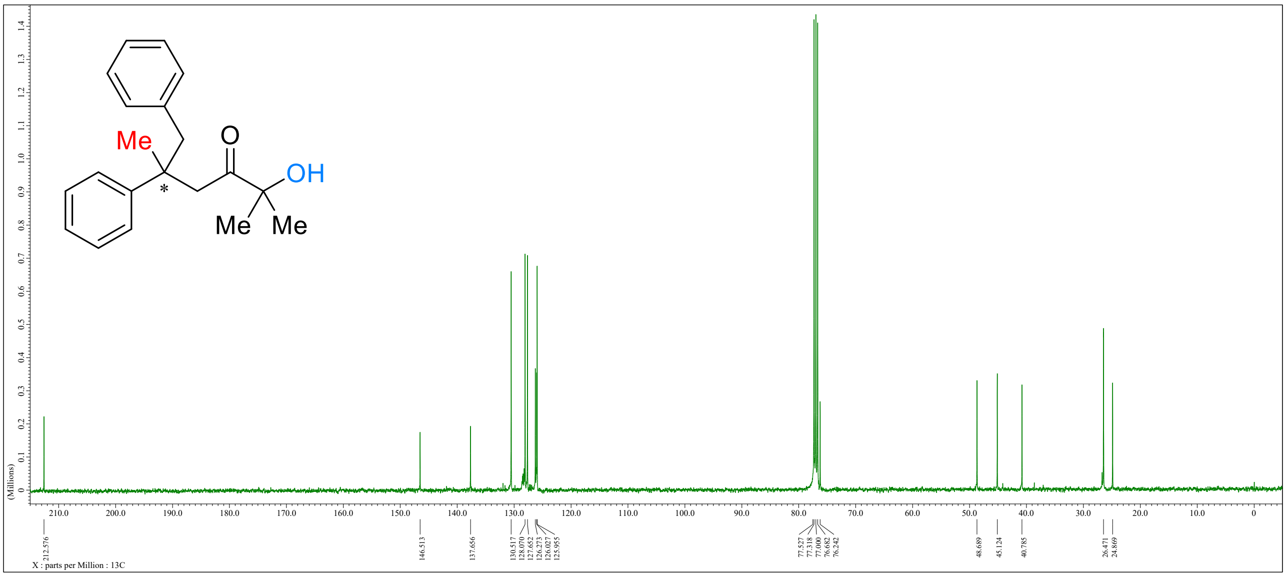

^{1}H NMR (400 MHz, $CDCl_3$) **2n**

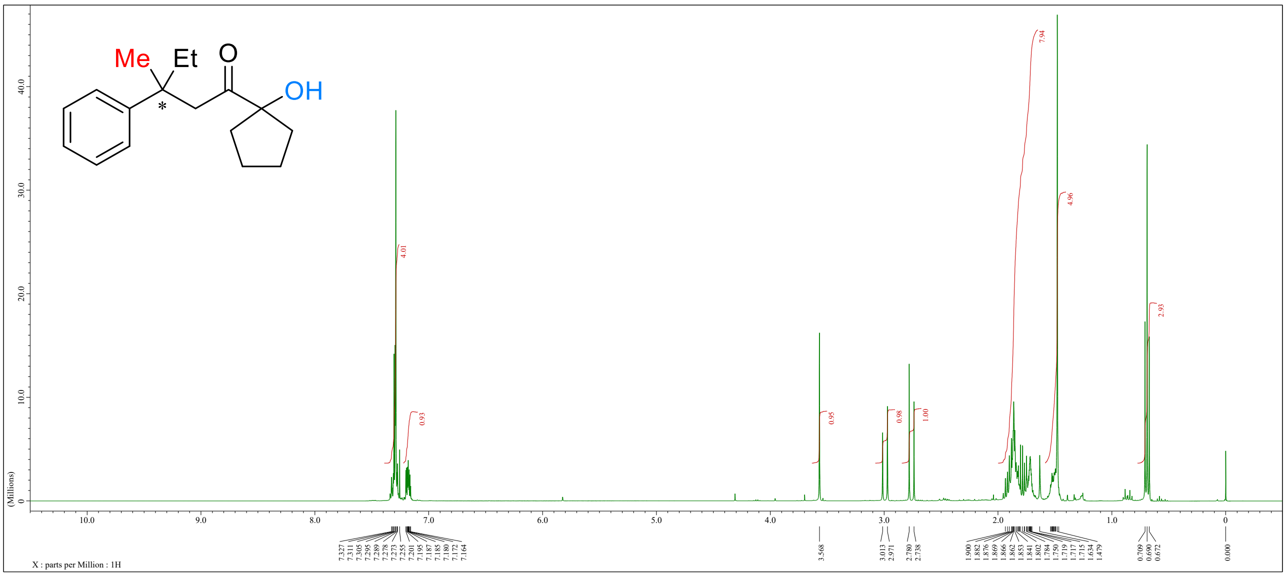

$^{13}C\{^{1}H\}$ NMR (100 MHz, $CDCl_3$) **2n**

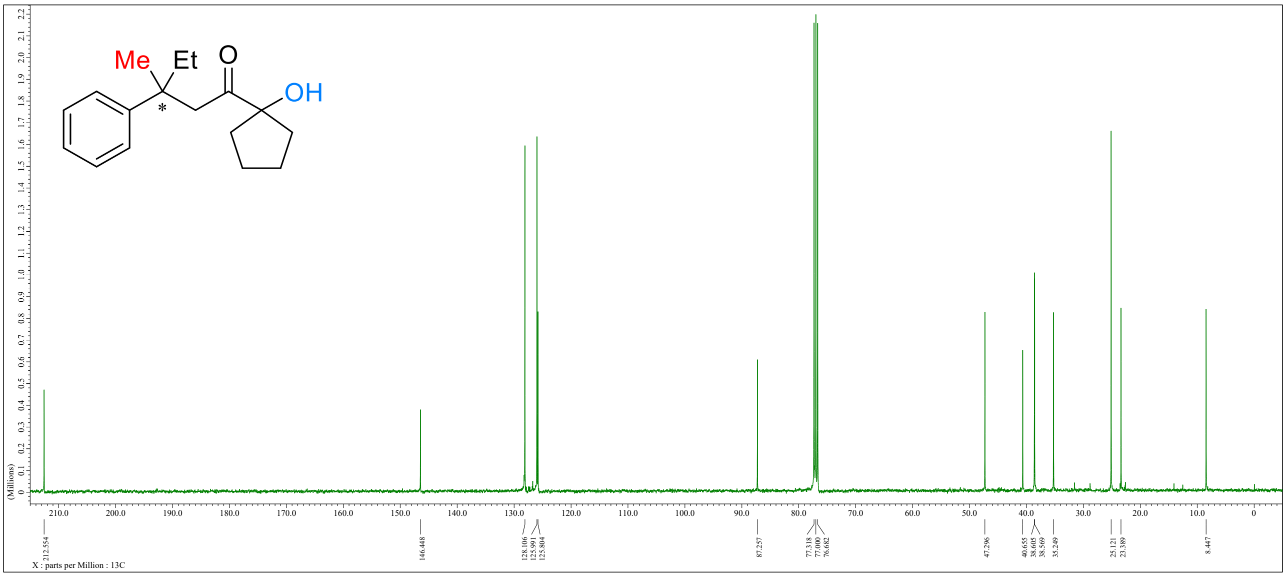

^{1}H NMR (400 MHz, $CDCl_3$) **2o**

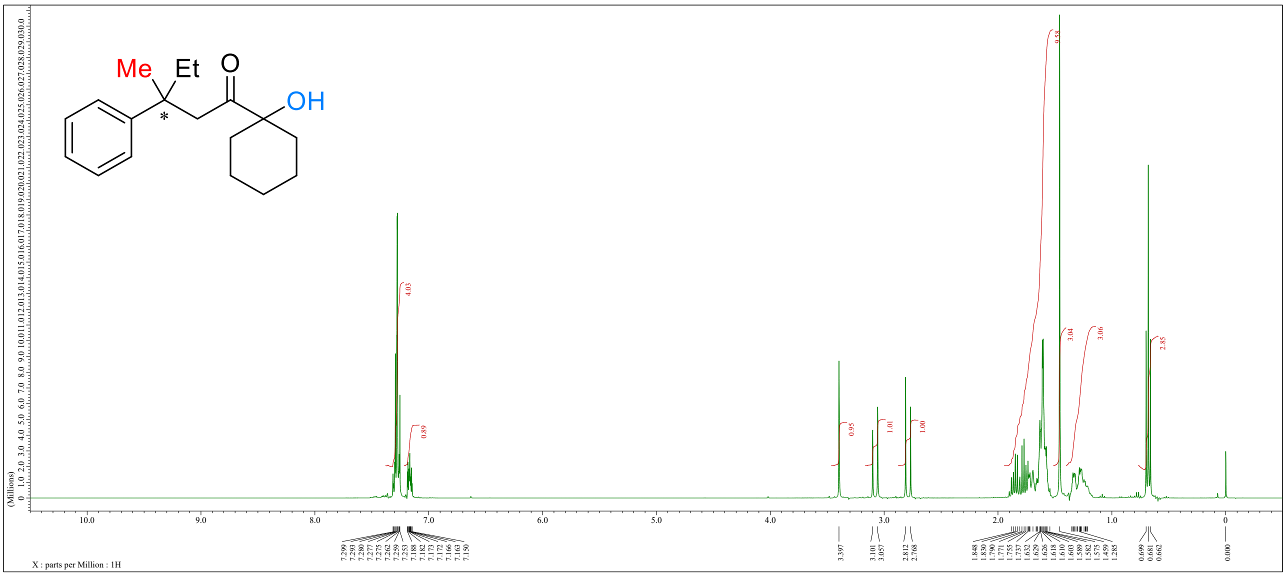

^{13}C{^{1}H} NMR (100 MHz, $CDCl_3$) **2o**

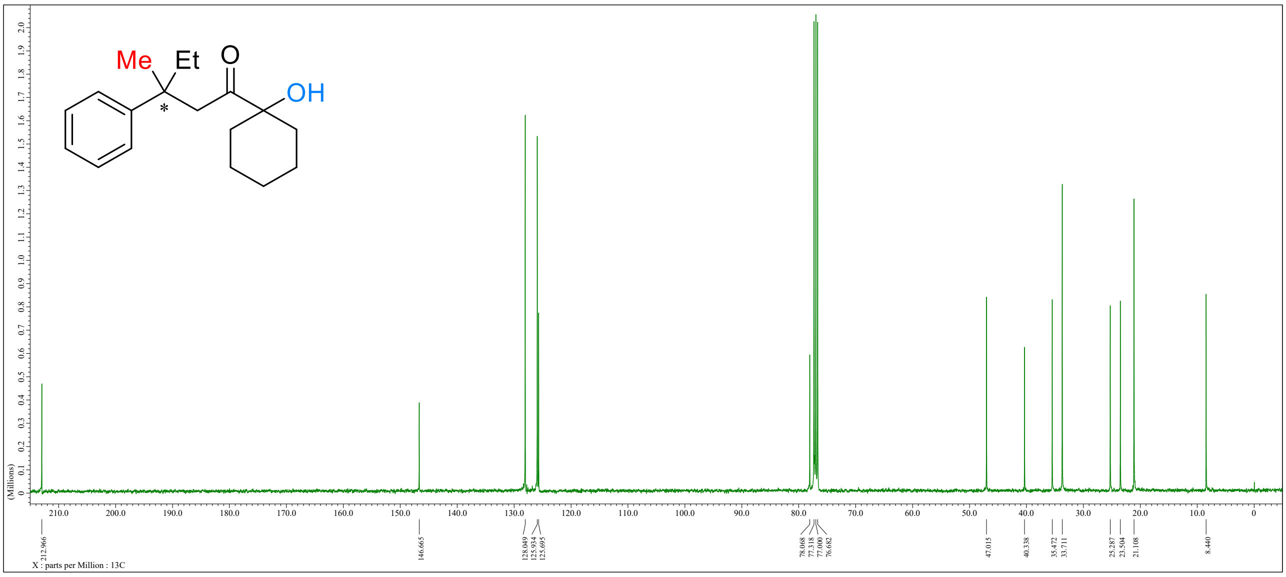

^{1}H NMR (400 MHz, $CDCl_3$) **8**

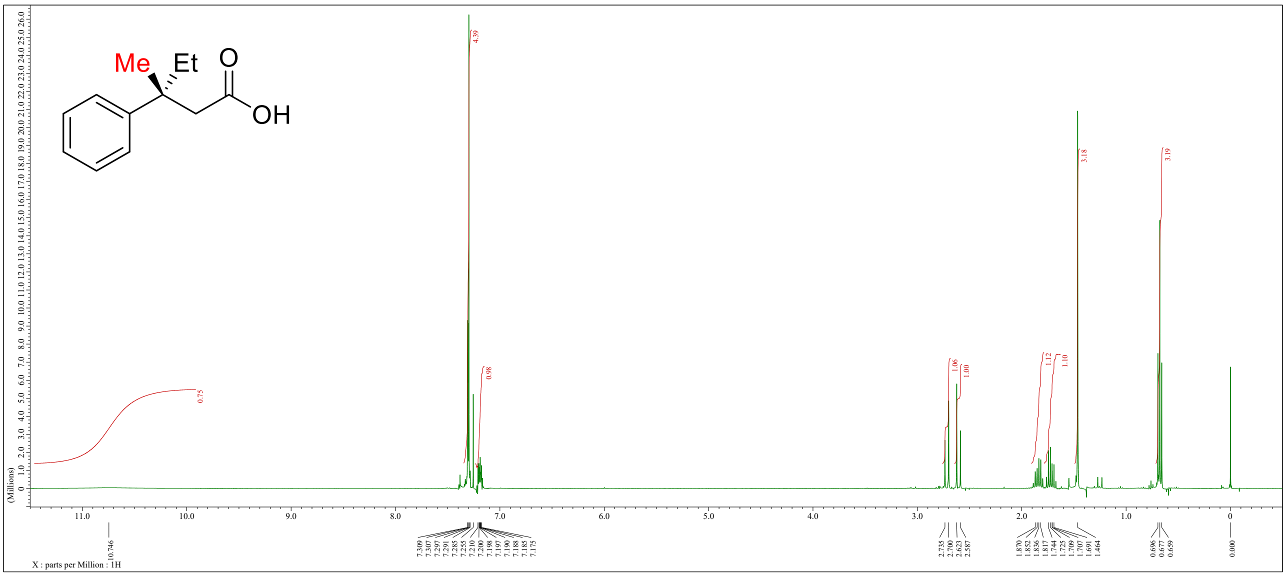

^{1}H NMR (400 MHz, $CDCl_3$) **9**

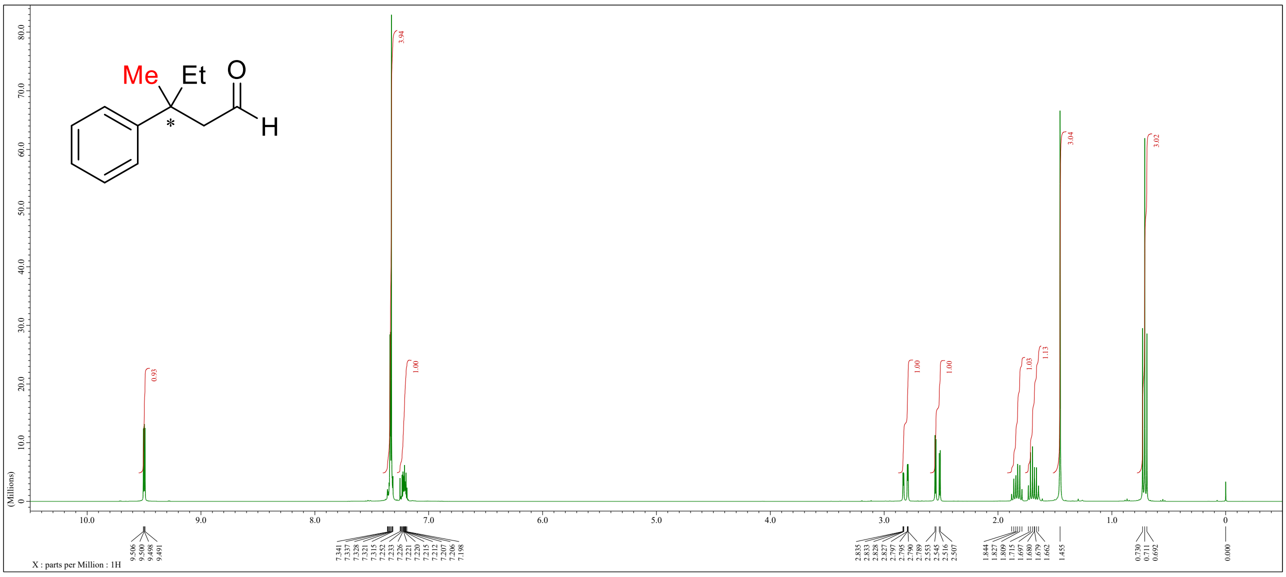

$^{13}C\{^1H\}$ NMR (100 MHz, $CDCl_3$) **9**

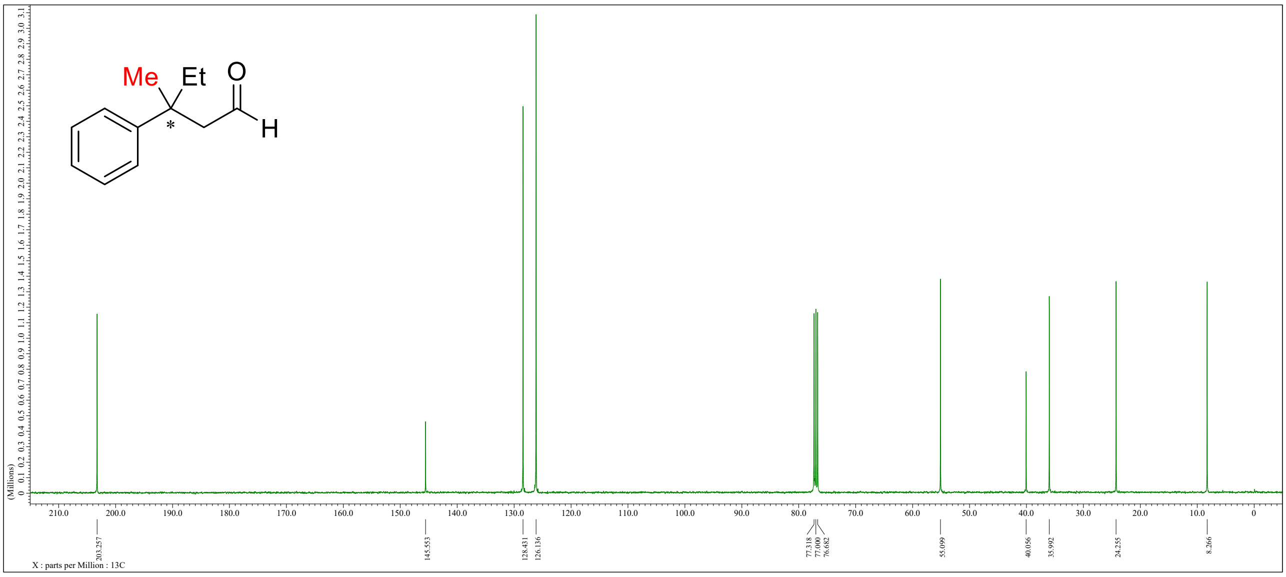

Supplement: Supplementary file 1 [file jo6c00144_si_001.pdf]
